# Smartphone Level Test Measures Disability in Several Neurological Domains for Patients With Multiple Sclerosis

**DOI:** 10.3389/fneur.2019.00358

**Published:** 2019-05-28

**Authors:** Alexandra K. Boukhvalova, Olivia Fan, Ann Marie Weideman, Thomas Harris, Emily Kowalczyk, Linh Pham, Peter Kosa, Bibiana Bielekova

**Affiliations:** ^1^Neuroimmunological Diseases Section, Laboratory of Clinical Immunology and Microbiology, National Institute of Allergy and Infectious Diseases (NIAID), National Institutes of Health (NIH), Bethesda, MD, United States; ^2^Department of Computer Science, University of Maryland, College Park, MD, United States

**Keywords:** multiple sclerosis, smartphone, sensors, mobile device, accelerometer

## Abstract

Our long-term goal is to employ smartphone-embedded sensors to measure various neurological functions in a patient-autonomous manner. The interim goal is to develop simple smartphone tests (apps) and evaluate the clinical utility of these tests by selecting optimal outcomes that correlate well with clinician-measured disability in different neurological domains. In this paper, we used prospectively-acquired data from 112 multiple sclerosis (MS) patients and 15 healthy volunteers (HV) to assess the performance and optimize outcomes of a Level Test. The goal of the test is to tilt the smartphone so that a free-rolling ball travels to and remains in the center of the screen. An accelerometer detects tilting and records the coordinates of the ball at set time intervals. From this data, we derived five features: path length traveled, time spent in the center of the screen, average distance from the center, average speed while in the center, and number of direction changes underwent by the ball. Time in center proved to be the most sensitive feature to differentiate MS patients from HV and had the strongest correlations with clinician-derived scales. Its superiority was validated in an independent validation cohort of 29 MS patients. A linear combination of different Level features failed to outperform time in center in an independent validation cohort. Limited longitudinal data demonstrated that the Level features were relatively stable intra-individually within 4 months, without definitive evidence of learning. In contrast to previously developed smartphone tests that predominantly measure motoric functions, Level features correlated strongly with reaction time and moderately with cerebellar functions and proprioception, validating its complementary clinical value in the MS app suite. The Level Test measures neurological disability in several domains in two independent cross-sectional cohorts (original and validation). An ongoing longitudinal cohort further investigates whether patient-autonomous collection of granular functional data allows measurement of patient-specific trajectories of disability progression to better guide treatment decisions.

## Introduction

There is a need for simple, but reliable, testing of diverse neurological functions to identify neurological disability in situations where a neurologist is absent (e.g., third world countries or rural areas), to track the disability of patients longitudinally, and to produce reliable outcomes for drug development. For the reasons outlined below, our long-term goal is to develop an integrated collection (i.e., Multiple Sclerosis [MS] suite) of simple tests (apps) that reliably measure disability in several/most neurological domains, using smartphone embedded censors in a patient-autonomous manner. This goal is approached via several interim aims: (1) To develop individual simple digital tests and to develop/optimize quantitative outcomes derived from the smartphone sensors/time values; (2) To validate clinical utility of developed outcomes by comparing them to the clinical gold standard represented by clinician-generated disability scores in specific neurological functions in cross-sectional cohort(s); (3) To integrate validated outcomes from multiple apps into a combined clinical score that correlates highly with existing, clinician-derived disability scores and that is also optimized for change in time, measured in longitudinal cohort(s).

Neurological scales currently used for drug development in complex neurological diseases, such as MS, fall into two categories: clinician-derived scales, such as the Expanded Disability Status Scale [EDSS; (1)], and non-clinician collected “functional scales,” such as the timed 25-foot walk (25FW), 9-hole peg test (9HPT), paced auditory serial addition test (PASAT), and symbol digit modalities test [SDMT; (2)]. Because traditional clinician-derived scales are insensitive and prone to bias, and each functional scale measures only selective domain(s) of neurological functions, the most recent trend in clinical trial methodology is to combine clinician-derived and functional scales into more accurate composite scales, such as EDSS-plus (3) or Combinatorial weight-adjusted disability scale [CombiWISE; (4)]. Unfortunately, time and expense constraints preclude collecting these optimized outcomes in routine clinical practices.

Current non-clinician collected functional tests can be replaced by simple tests amenable to autonomous collection by patients via smartphones, such as finger and foot tapping, without significant loss of accuracy (5–8). We recently demonstrated that digitalizing these tests offers additional benefits: not only can the test be performed by patients in frequent intervals outside of clinic (i.e., granular collection), but input from smartphone sensors and internal clocks can be assembled into novel, secondary outcomes. For example, the application can be used to measure motor fatigue based on declining test performance across time (9).

In neurological diseases, where pathological processes are widespread across the central nervous system (CNS), many or potentially all neurological functions are affected. This makes functional tests that utilize several neurological functions simultaneously (e.g., in the case of 9HPT, vision, reaction time, strength, and cerebellar functions) slightly more sensitive in comparison to simpler tests, such as finger tapping (9). The ceiling effect and high variability are natural drawbacks of these more complex functional tests. Additionally, while increased sensitivity is desired when the test is used as an outcome in clinical trials, identification of affected neurological domains becomes impossible for a test that integrates many neurological functions. We hypothesized that disability in specific neurological functions may be decoded from an assembly of multiple simple tests using statistical learning. We tested this hypothesis in the first two simple tests (apps) we implemented in the MS suite, and we observed that, when comparing outcome(s) from a test that focuses exclusively on motoric functions (i.e., finger tapping) with a test that employs also the visual system and reaction time (i.e., balloon popping), we were able to identify patients with cognitive or visual dysfunction exceeding motoric disability (9).

The current paper describes the Level Test, a third, simple, functional test of upper extremities in the MS test suite. Patients were instructed to direct a free-rolling ball to the center of the screen with the shortest possible time/path and keep the ball there for the remainder of the test. The Level Test emphasizes cerebellar and proprioceptive functions as well as cognitive reaction time.

## Methods

### Application Development

**Figure 1:**
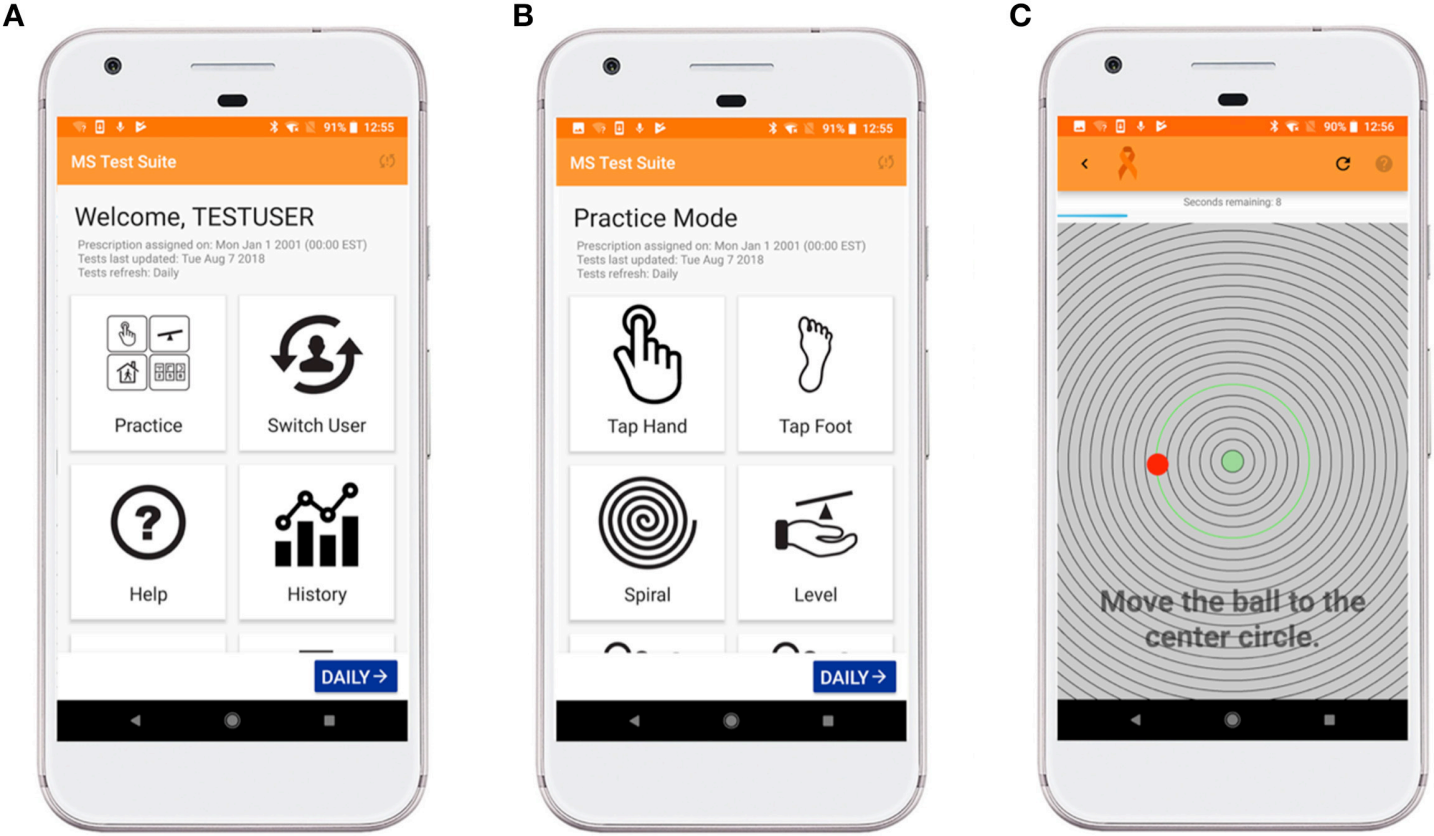
MS test suite user-interface for Level Test. **(A)** This interface allows the operator to enter practice mode, switch users, access help tutorials, visualize a patient's trend with time, and submit feedback regarding application bugs. **(B)** The suite currently contains nine different tests, which can be accessed in Practice Mode (shown here) or Trial Mode. These tests refresh daily, and the clinician can change a patient's prescription to assign difficulty Levels 1 (easiest) −3 (most difficult) or to skip an appendage. **(C)** The Level Test interface displays a countdown and instructions on how to complete the test. The time remaining is displayed in a blue bar across the top of the screen. The user controls the red ball, which is randomly generated at a point 450 pixels (px) from the center of the screen. As the ball moves across the screen, the nearest concentric circle is highlighted in green. The goal of the test is to tilt the phone so that the red ball travels to and remains in the green center of the screen.

The Level Test was written in Java using the Android Studio integrated development environment. It underwent iterative development and optimization following beta testing with developers and then clinical trial testing with patients and healthy volunteers. The test is an Android Package (APK) downloaded by a different APK that served as a front-end (Figure 1), which could be installed over email or a direct USB connection with Android Studio. This front-end collected user information such as their testing ID, birth month and year, gender, and dominant hand. It also read “prescriptions” from a cloud-based spreadsheet, which configured the Level Test for each user, tailored to their disability level. Installation and initial testing were originally completed on a variety of personal Android phones, with no particular specifications. Testing in the clinic with patients was done with Google Pixel XL (2017) phones running the Android 8.1 Oreo operating system that were kept fully up to date.

### Patient Populations

This study was approved by the Combined Neuroscience Institutional Review Board of the National Institutes of Health (NIH), and all participants signed either a paper or digital informed consent.

Patients were enrolled in one of two protocols: Targeting Residual Activity by Precision, Biomarker-Guided Combination Therapies of Multiple Sclerosis (TRAP-MS; clinicaltrials.gov identifier NCT03109288) and Comprehensive Multimodal Analysis of Neuroimmunological Diseases of the Central Nervous System (NCT00794352). Most healthy volunteers (HV) participated in a healthy volunteer sub-study of NCT00794352 to obtain normative data for the smartphone applications. There were no inclusion/exclusion criteria because the sub-study recorded no personally identifiable information (PII), so all participating subjects were self-declared as not having any known neurological deficit.

Data was collected from 112 MS patients and 15 HV, for a total of 494 and 448 individual trials in the left and right hand (prior to outlier removal), respectively, for the full/main cohort. Tables 1, 2 display the demographics and clinical features for data used in the analyses. After outlier removal (see Data Analysis), of the subjects from the main cohort, 15 HV and 32 MS patients satisfied the *longitudinal* criteria (at least 3 sittings) with a mean/median of 9.3/7 sittings (range of 6–23) in the HV cohort and mean/median of 8.1/3.5 sittings (range of 3–25) in the MS cohort. To be classified as a sitting, the trials must have occurred on different days.

**Table 1:** Baseline demographics for the cross-sectional MS participants whose data were used in computing correlations.

**Demographics**	**Cross-sectional MS (*****N*** **=** **93)^a^**		
	**PPMS (*n* = 38)**	**SPMS (*n* = 19)**	**RRMS (*n* = 35)**
Age—year			
Mean ± SD	58.8 ± 7.8	60.0 ± 8.2	50.0 ± 10.2
Median	58.8	60.7	49.5
Range	28.2–70.8	41.9–71.9	30.2–77.2
Gender—no. of patients (%)			
Female	52.6	57.9	54.3
Male	47.3	42.1	45.7
**Other clinical characteristics**			
Disease duration—year			
Mean ± SD	15.7 ± 8.3	26.8 ± 11.2	12.4 ± 10.1
Median	14.6	29.4	9.0
Range	4.2–44.2	5.0–48.9	1.1–43.4
NeurEx^b^			
Mean ± SD	94.0 ± 61.8	119.0 ± 68.9	35.1 ± 35.6
Median	79.9	102.4	24.2
Range	10.7–308.4	24.4–263.1	2.5–171.7
EDSS^b^			
Mean ± SD	5.7 ± 1.3	6.0 ± 1.1	3.3 ± 1.6
Median	6.0	6.5	3.0
Range	2.5–8.0	3.5–7.5	1.0–6.5
SNRS^b^			
Mean ± SD	56.5 ± 11.2	54.1 ± 10.2	74.4 ± 12.3
Median	59.0	54.0	74.0
Range	29.0–85.0	39.0–74.0	41.0–98.0
CombiWISE^b^			
Mean ± SD	48.1 ± 13.4	50.5 ± 12.1	27.1 ± 11.5
Median	46.6	53.6	26.0
Range	18.6–80.6	27.7–73.8	8.4–55.9
SDMT^b^			
Mean ± SD	39.7 ± 11.9	39.5 ± 12.3	49.5 ± 14.7
Median	42.0	42.0	49.0
Range	17.0–61.0	9.0–60.0	23.0–84.0
MSFC^b^			
Mean ± SD	−1.2 ± 2.2	−0.9 ± 1.6	0.2 ± 0.6
Median	−0.2	−0.5	0.3
Range	−6.8–0.9	−0.63–0.9	−1.1–1.0
9HPT^b^			
Mean ± SD	49.1 ± 59.0	44.4 ± 44.3	22.9 ± 6.4
Median	26.8	26.7	21.6
Range	18.6–274.2	18.2–166.2	15.8–52.2
25FW^b^			
Mean ± SD	38.7 ± 59.5	30.4 ± 40.9	5.7 ± 2.7
Median	8.5	15.1	4.8
Range	4.0–179.9	3.7–179.9	3.6–15.6
PASAT^b^			
Mean ± SD	44.5 ± 13.7	47.7 ± 12.4	46.7 ± 12.3
Median	48.5	51.0	50.0
Range	6.0–60.0	14.0–60.0	22.0–60.0

*Patients were split by MS disease subtype into primary progressive MS (PPMS), secondary progressive MS (SPMS), and relapsing remitting MS (RRMS). Data are represented as sample size (%) or mean ± standard deviation, median and range. Percentages do not always sum to 100 due to rounding error. NeurEx is obtained from an app-based documentation of the neurological exam (10). EDSS (1) denotes Expanded Disability Status Scale. SNRS (11) denotes Scripps Neurologic Rating Scale. CombiWISE (4) denotes Combinatorial Weight-Adjusted Disability Score. SDMT (2) denotes Symbol Digit Modalities Test. MSFC (12) denotes Multiple Sclerosis Functional Composite. 9HPT denotes 9-Hole Peg Test. 25FW denotes 25-Foot Walk Test. PASAT denotes Paced Auditory Serial Addition Test. Only demographics for patients with valid clinical scores at the first test sitting were summarized in this table (thus, N = 93 instead of N = 112), as patients could only be used in computing correlations if these clinical scores were available*.

a *MS patients without valid clinical scores were excluded from the tabulation. Cross-sectional cohort includes one additional patient with unknown MS subtype not summarized in table*.

b *Descriptive statistics were calculated by excluding missing data*.

**Table 2:** Demographics for full cohort.

**Cohort**	***N***	**Female (%)**	**Average disease duration (year)**	**Average NeurEx^a^**	**Average EDSS^a^**
**HEALTHY VOLUNTEERS**					
18–29	5	60.0	−	−	−
30–39	2	0.0	−	−	−
40–49	2	50.0	−	12.9	1.5
50–59	3	100.0	−	18.0	1.5
>60	3	33.3	−	32.2	2.0
**MS PATIENTS**					
18–29	2	50.0	11.1	251.5	5.5
30–39	7	85.7	5.7	28.6	1.9
40–49	25	64.0	10.2	80.3	3.6
50–59	40	50.0	15.0	123.9	4.5
>60	38	60.5	20.7	160.4	5.5

*Patients are divided into healthy and MS cohorts stratified by age. Average disease duration and clinical scales are calculated where applicable. Percentages do not always sum to 100 due to rounding error. NeurEx (10) is obtained from an app-based documentation of the neurological exam. EDSS (1) denotes Expanded Disability Status Scale*.

a *Descriptive statistics were calculated by excluding missing data*.

Data was collected from 29 MS patients, for a total of 186 and 185 individual trials the left and right hands (prior to outlier removal), respectively, in an independent validation cohort. Supplementary Table 1 displays the demographics and clinical features for data used in the validation.

### Patient Instructions

Trained laboratory personnel administered the test to patients during their first session (i.e., cross-sectional cohort). Interested MS patients were able to take a phone home (i.e., longitudinal cohort) to complete the tests at least once per week. Patients with phones at home were instructed to bring the phones to another in-clinic visit for necessary updates after 6 months.

Patients were given the following set of instructions when they were first administered the Level Test (see Supplementary Video). As these instructions were explained, laboratory personnel demonstrated how to correctly perform the test. These instructions were also displayed on the screen at the beginning of testing.

1. You will complete two trials with your left hand and two trials with your right hand.
2. Hold the phone steady through the 3-s countdown. The test will begin after the countdown.
3. The goal of this test is to tilt the phone so that the red ball rolls to the green circle in the center of the screen.
4. Once the ball is in the green circle, keep the ball there for as long as possible until the 10-s timer is finished.
5. You will be given the option to cancel and retake the trial if you are not satisfied with your results. Otherwise, you may submit your results for the clinician to review.

Patients were allowed to complete the tests on practice mode before completing the recorded exam.

### Derivation of Level Test Features

**Figure 2:**
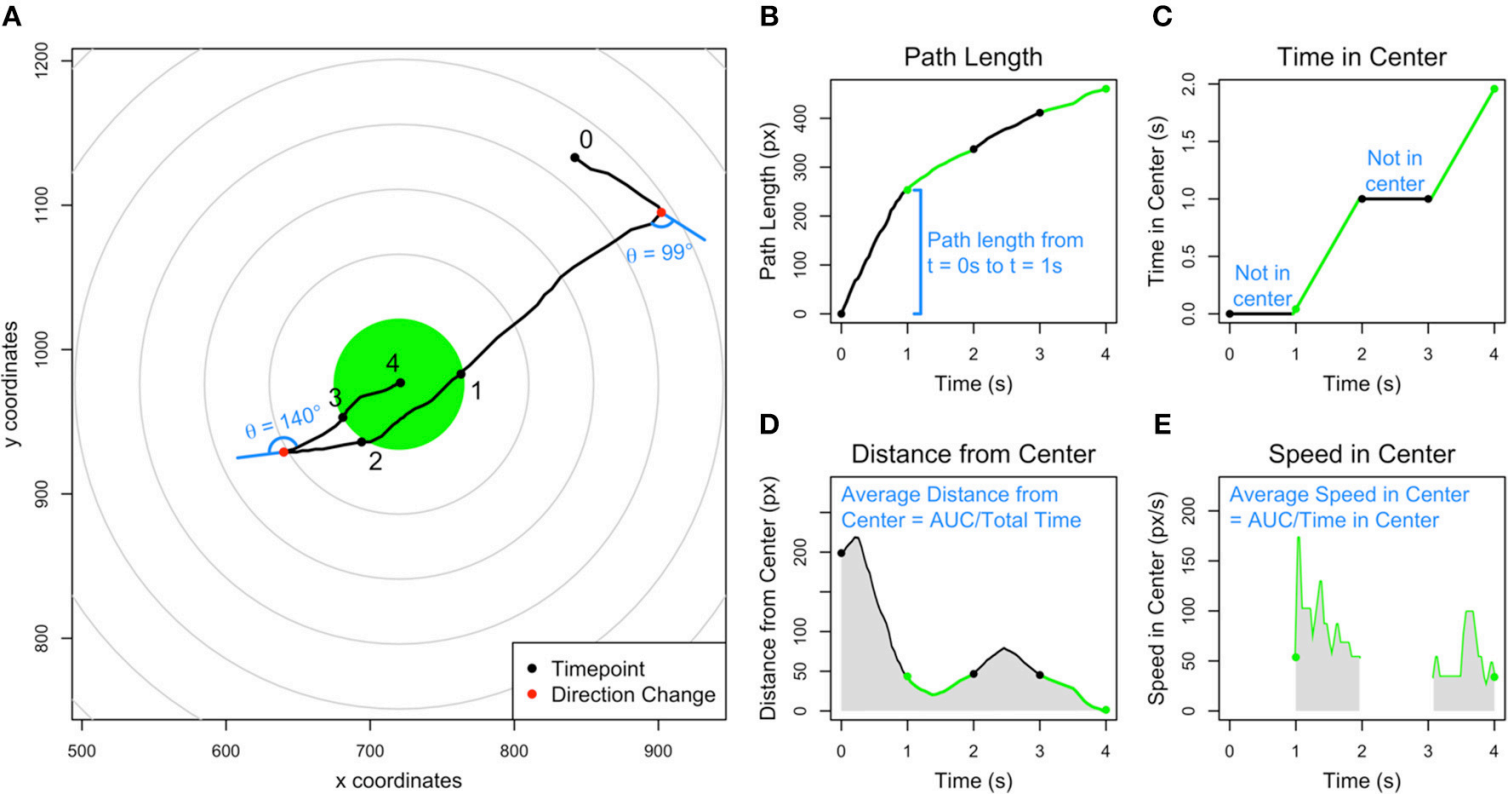
Explanation of the five Level Test features. **(A)** Simplified example of a Level Test trial over 4 s, with each second labeled in a black point. The ball crosses into the center, highlighted in green, at *t* = 1 s and 3 s, and crosses out of the center at *t* = 2 s. The center is defined as a circle of radius 45 px. Two direction changes are labeled in red points, and the angle changes are denoted in blue. The angle changes are measured as the deviation from the original path along a straight line. **(B)** Graph of the path length of the ball corresponding to the trial in **(A)**. The sections where the ball is outside of and inside the center are labeled in black and green, respectively. **(C)** Graph of the time in center of the ball corresponding to the trial in **(A)**. When the ball is not in the center, the time in center is unchanging (indicated by horizontal black lines), and when the ball is outside of center, the time in center increases (indicated by green lines). **(D)** Graph of the distance from center of the ball corresponding to the trial in **(A)**. The average distance from center is calculated by dividing the area under the curve (gray shaded region) by the total time of the trial. **(E)** Graph of the speed of the ball while in the center corresponding to the trial in **(A)**. The average speed from center is calculated by dividing the area under the curve by the time in center of the ball. The ball was outside of the center between 0–1 and 2–3 s (white space) and inside of the center between 1–2 and 3–4 s (gray shaded regions).

A simplified example of a single trial of the Level Test is given in Figure 2A. Point 0 represents the starting position and point 4 the final position of the ball. The smartphone app recorded two precalculated features: the path length traveled (Figure 2B) and the amount of time spent within 45 px of the center of the screen (Figure 2C). The test also recorded the Euclidean coordinates of the ball's position on the screen at intervals of 16 ms. Using these coordinates, we derived three new features: average distance from the center, average speed while within 45 px of the center, and number of directional changes undergone throughout the trial. The average distance from center (Figure 2D) was calculated by plotting distance from the center at each time point and dividing the area under curve (AUC) by the total time of the trial. The average speed in center (Figure 2E) was calculated by plotting the speed of the ball if it was within 45 px of the center at each time point and dividing the AUC by the amount of time the ball spent within 45 px of the center. The number of directional changes (see legend in Figure 2A) was calculated by first “smoothing” the path of the ball by only taking time points at intervals of n/200 (rounded to the nearest integer), where n represents the original number of time points. In doing so, we retained approximately 200 time points while using a constant interval. From the modified set of time points, we considered each consecutive triple of coordinates and calculated the angle change from 180 degrees (i.e., a straight line). If the angle change was >45 degrees, and the ball was not within 90 px of the center at all three coordinates, we counted the instance as a direction change. We chose these criteria after attempting combinations of 25/100/200 time points, 30/45 degrees, and 45/90 pixels and then optimizing for correlations with established clinical scales and specific neurological functions.

### Clinical Data

Traditional clinical scales, such as EDSS (1), Scripps Neurological Rating Scale [SNRS; (11)], 25FW, 9HPT, and the cognitive scales Symbol Digit Modalities Scale [SDMT; (2)] and Paced Auditory Serial Addition Test (PASAT) were collected at each clinic visit by MS-trained clinicians and laboratory investigators and inputted in real-time to the Neuroimmunological Diseases section (NDS) research database. The composite scales, MS Functional Composite [MSFC; (12); combines 25FW, 9HPT, and PASAT] and Combinatorial Weight-Adjusted Disability Scale [CombiWISE; (4); combines EDSS, SNRS, 25FW and non-dominant hand 9HPT], were computed automatically by the database from inputted data.

NeurEx scores (10) were computed from an app that divides the standard neurological examination into 17 functions systems, each one displayed on a single iPad page, where a clinician conveniently documents his/her findings using touch gestures on human body diagrams that display features in real spatial distribution. NeurEx integrates this spatial information with fully quantitative (e.g., vibration sense) or semi-quantitative (i.e., mild, moderate, and severe) grading of observed neurological deficit to derive a final score for each subsystem. Because all aspects of the examination are digitized in NeurEx, it is easy to derive side or limb-specific subscores for each neurological function.

### Data Analysis

We partitioned the data into two cohorts: cross-sectional (*N* = 127, 112 MS + 15 HV; Table 1) and longitudinal (*N* = 47, 32 MS + 15 HV; Table 2). The cross-sectional cohort consisted of trials completed by all patients during their baseline visits, while the longitudinal cohort consisted of all trials completed by patients with trials over at least three distinct dates. An independent cross-sectional cohort consisting of subsequently acquired 29 MS patients was used for validation (Supplementary Table 1), and again, consisted of trials completed only during the baseline visit. We removed outliers intra-individually (within patients) in a two-step process. First, we removed all trials with path length > Q3 + 1.5IQR (where Q3 is quartile three and IQR is the interquartile range) or time in center < Q1 – 1.5IQR (where Q1 is quartile one). This removed outliers from the longitudinal dataset. Then, for cross-sectional data (comprised of two trials per patient from the baseline visit), we excluded all initial trials that were at least two times worse than the second trial based on path length or time in center. Specifically, we excluded one of the two trials if the path length of the first trial was more than twice as long as the second trial or if the time in center was less than half as long as the second trial.

Correlation coefficients presented in the text were calculated using Spearman's rank correlation and denoted by *r*_*s*_. The *p*-values associated with these correlations were adjusted column-wise for multiple comparisons using false discovery rate (FDR). Differences between the groups for the cross-sectional data were determined using a Wilcoxon rank-sum test, a non-parametric test which counts the number of times an individual HV score is greater/less than each MS score. Differences between the groups for the longitudinal data were determined by performing analysis of variance on a mixed-effects model. The latter controls for repeated measures by using the following:

(1) $$\begin{array}{l} Feature=Group+Days+\left(Days|Patient\;ID\right) \end{array}$$

where feature represents one of the five Level Test features, group is MS or HV, and days is the number of days since the first test sitting. The second fixed-effects term (Days) controls for any potential learning that could occur during the initial test sittings. The random-effects term (Days|Patient ID) allows for a distinct slope and intercept across time for each patient.

To create a linear combination of the best features, we employed a genetic algorithm (GA) using the GA package (13) in the statistical software R (14). This search algorithm uses the principles of natural selection to select the fittest features over the weakest features, and then each of these features are assigned a weight according to their relative importance to the model. We ran the GA in parallel utilizing the computational resources of the NIH HPC Biowulf Cluster (http://hpc.nih.gov).

## Results

### Correlations Between Level Test Features and Clinical Scales

**Figure 3:**
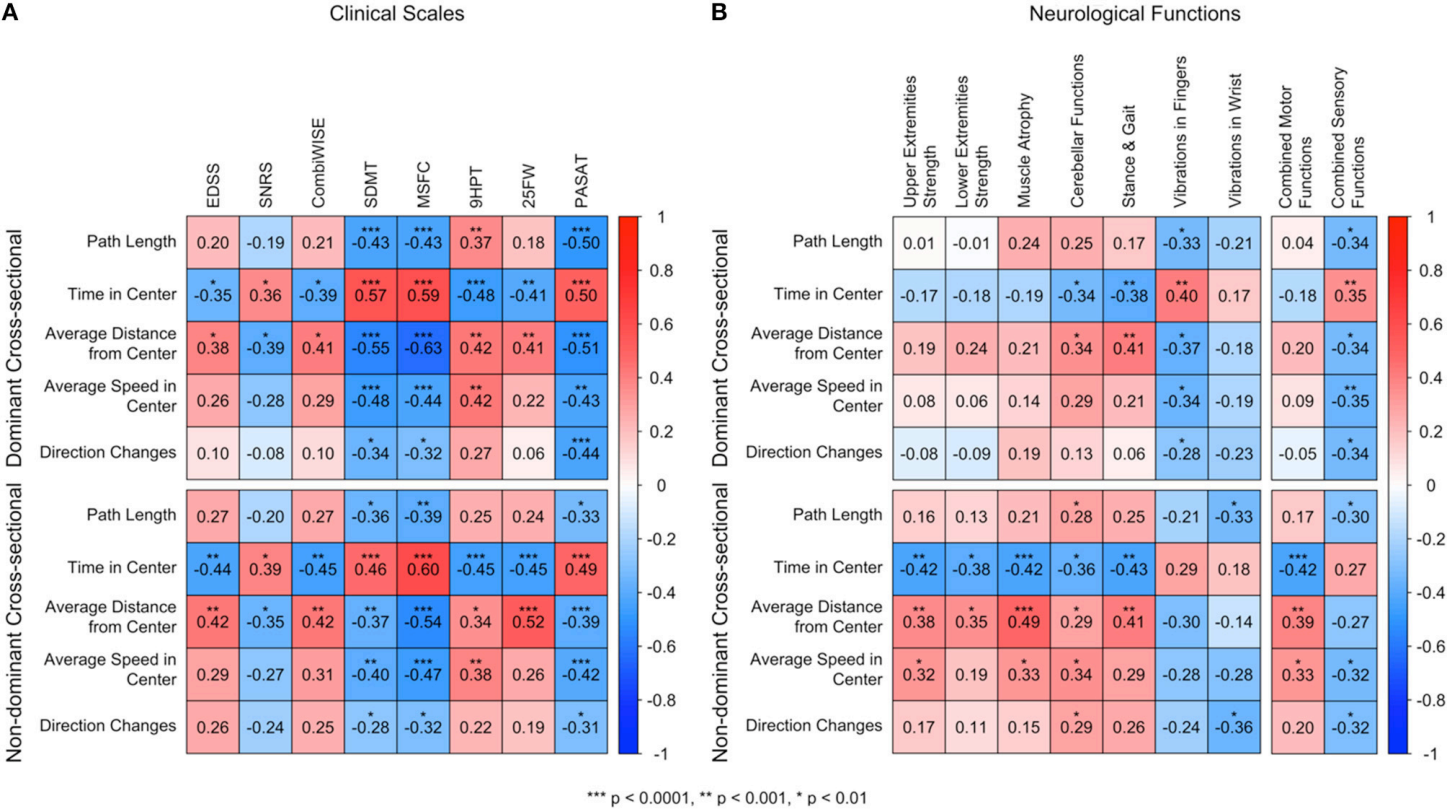
Correlation matrices against clinical scales and neurological functions. **(A)** Spearman correlations of the Level features against eight clinical scales: Expanded Disability Status Scale [EDSS (1)], Scripps Neurological Rating Scale [SNRS; (11)], Combinatorial Weight-Adjusted Disability Score [CombiWISE; (4)], Symbol Digit Modalities Test [SDMT; (2)], Multiple Sclerosis Functional Composite [MSFC; (12)], 9-Hole Peg Test (9HPT), 25-Foot Walk (25FW), Paced Auditory Serial Addition Test (PASAT). The 9HPT scores use the dominant/non-dominant hand score. **(B)** Spearman correlations of the Level features against neurological features. The Stance and Gait scores use the entire body score. The following functions use the dominant/non-dominant side score: Upper Extremities Strength, Lower Extremities Strength, Muscle Atrophy, Cerebellar Functions, Vibration in Fingers, and Vibration in Wrist. For all correlations red colors correspond to positive correlations, and blue colors correspond to negative correlations. All p-values are adjusted column-wise using false discovery rate and are categorized as ^***^p < 0.0001, ^**^p < 0.001, ^*^p < 0.01. The time in center and average distance from center, for both dominant and non-dominant hands, had strong, significant correlations with all 8 analyzed clinical scales. The strongest correlations were observed for cognitive scales SDMT and PASAT and the composite scale MSFC. The remaining Level features also correlated with cognitive scales and MSFC, even though the strengths were overall lower. The correlations with clinical scales were comparable for dominant and non-dominant hands.

In the cross-sectional data, we analyzed spearman correlations between clinical scales and the five Level Test features for the dominant and non-dominant hands (Figure 3A). Those features that correlate positively with clinical scales are colored in red, and those that correlate negatively with clinical scales are colored in blue. Overall, we observed the strongest correlations with traditional disability scales for two of the Level Test features: time in center and average distance from the center. The strongest correlations were observed for tests of cognitive disability (i.e., SDMT but also PASAT) and for a composite disability scale, MSFC. Time in center also correlated with traditional clinical scales that are biased toward motoric functions and gait, such as EDSS, 9HPT, and 25FW, as well as with the statistical-learning-optimized Combinatorial Weight-adjusted clinical scale, CombiWISE (4). These correlations were generally stronger for the non-dominant hand.

These observations were verified in a smaller independent validation cohort (Supplementary Figure 1), attesting to their robustness and reproducibility.

### Correlations Between Level Test Features and Components of the Neurological Exam

Specific neurological systems, derived from the digitalized documentation of neurological examination [i.e., NeurEx App; (10)] performed by MS-trained clinicians, were examined against all Level Test features (Figure 3B, Supplementary Figure 2).

Again, we observed the strongest correlations for time in center and average distance from center Specifically, time in center and average distance from center were moderately correlated with cerebellar dysfunction (Figure 3B, column 4) in the dominant hand (*r*_*s*_ = −0.34, p = 0.003 and *r*_*s*_ = 0.34, p = 0.003, respectively) and all features were moderately correlated with cerebellar dysfunction in the non-dominant hand. We also observed correlations between time in center and the stance and gait subdomain and average distance from center and the stance and gait subdomain of the neurological examination (Figure 3B, Column 5). It is likely that correlations with stance and gait resulted from cerebellar dysfunction and proprioceptive loss in both upper and lower extremities in the most disabled MS patients.

With the remaining relevant functions, we observed a clear dichotomy between dominant and non-dominant hands (Figure 3B). For the dominant hand, we observed moderate correlations between all Level features and the quantitative vibration sense in corresponding hands, indicating that, for the dominant hand, proprioception plays an important role in Level Test performance. Time in center had the strongest and most significant positive correlation with vibration in the fingers (*r*_*s*_ = 0.40, *p* = 0.00052). In contrast, for the non-dominant hand, we observed moderate correlations between motoric domains of the corresponding hand, such as muscle strength and muscle atrophy, for most of Level Test features. Specifically, the features time in center, average distance from center, and average speed in center were moderately correlated with the non-dominant upper extremity strength, lower extremity strength, and muscle atrophy (Figure 3B, columns 1–3), with the highest correlations between time in center (*r*_*s*_ = −0.42, *p* = 0.00022 and *r*_*s*_ = −0.42, *p* = 9.8*e*−5, respectively) and average distance from center (*r*_*s*_ = 0.38, *p* = 0.00067 and *r*_*s*_ = 0.49, *p* = 7.4*e*−6, respectively). This dichotomy is obvious when one compares correlations of global motor and sensory functions with Level Test features (Figure 3B, last columns).

### Level Features Discriminate Between HV and MS Patients Cross-Sectionally

**Figure 4:**
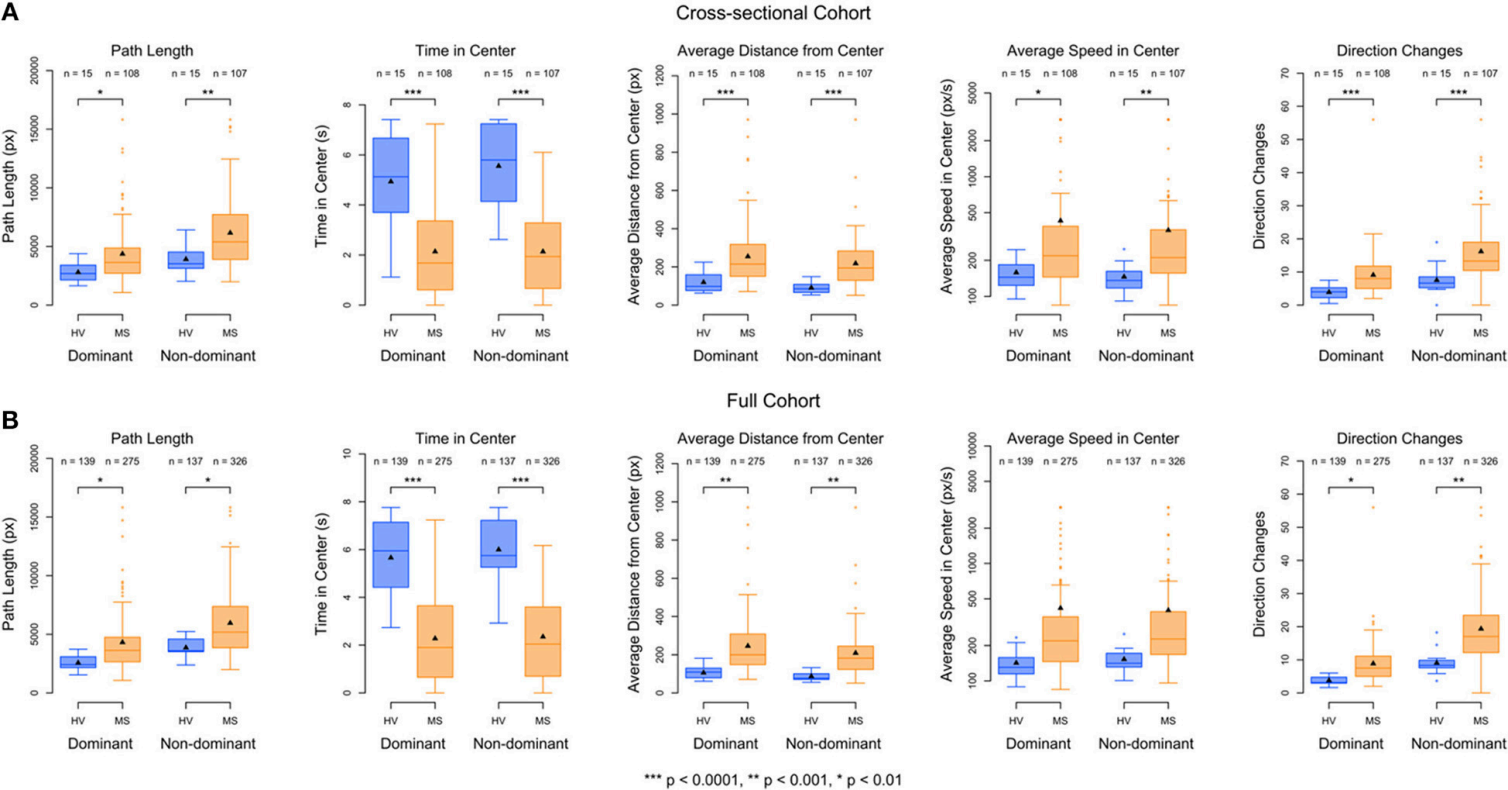
Boxplots of the Level features, separated by dominant/non-dominant hand and HV/MS cohort. **(A)** Cross-sectional data shown consists of the average of the two trials from the baseline visit. Differences between the groups were determined using a Wilcoxon rank-sum test. **(B)** Longitudinal data shown consists of the average of all scores within each patient. Differences between the groups for the longitudinal data are determined by performing analysis of variance on a mixed-effects model (see Equation 1 in Methods) using all scores for each patient. The number of trials for each group is denoted above each boxplot. Differences between the sample sizes of the groups across dominant/non-dominant hands are due to both inconsistent completion of the Level Test as well as outlier removal. The significance of the ability of each feature to discriminate between the HV (blue) and MS (orange) patients are calculated with a mixed-effects model with ^***^p < 0.0001, ^**^p < 0.001, ^*^p < 0.01. Lower scores for path length, average distance from center, average speed in center, and direction changes correspond to better performance, while higher scores for time in center correspond to better performance.

The best performing Level Test features, listed in order, were time in center, average distance from center, and direction changes. Non-dominant hand time in center most significantly differentiated MS patients from HV (*p* = 2.1e−7, Wilcoxon rank−sum test), followed closely by average distance from center for the non-dominant hand (*p* = 8.6*e*−7, Wilcoxon rank−sum test, Figure 4A). Dominant time in center and non-dominant direction changes had similar discriminatory power (*p* = 1.7*e*−5 and *p* = 1.1*e*−5, Wilcoxon rank-sum test, Figure 4A). When examining each feature individually, the non-dominant hand always outperformed the dominant hand.

Again, this hierarchy of outcomes was preserved in the small, independent validation cohort, affirming the robustness of our conclusions (Supplementary Table 3, Supplementary Figure 3).

To explore whether we could derive a composite feature (based on an optimized linear combination of features) from Level Test features with enhanced clinical utility, we performed a genetic algorithm (GA) by optimizing the difference between the MS and HV cohorts (see Methods). The GA determined that the relative weight of non-dominant time in center was approximately 1,000-fold higher than the relative weights of all remaining Level Test features (Supplementary Table 2). Thus, inclusion of the remaining nine features in a model to differentiate between the groups would only add variance without enhancing performance.

### Level Test Features Discriminate Between HV and MS Patients Longitudinally

Ultimately, as described in the introduction, our goal is to optimize app-generated outcomes for measuring progression of neurological disability in time, ideally by integrating validated outcomes from several/all developed apps. We do not have sufficient longitudinal data for this aim collected thus far. Instead, we used limited longitudinal data available to: (1) Validate the clinical utility of the most discriminatory features identified in the cross-sectional cohort in the setting of granular collection of data in patient-autonomous manner (i.e., outside of the clinic, in patients' homes); and (2) Assess the level of intra-individual variability of the best-performing Level Test features in short-term granular acquisition. This short-term data could also demonstrate the presence of a “learning” effect.

As was seen in the cross-sectional data, the best performing features for the full cohort, in order, were time in center, average distance from center, and direction changes. However, non-dominant time in center far out-performed any other feature (*F* = 71, *p* = 5.8*e*−13, Figure 4B) in differentiating MS from HV across time.

Once we have gathered enough longitudinal data, we would like to use Level features (likely in combination with other App-based outcomes as a single composite scale) to track disability changes with time. In the absence of disease progression, the features useful for accurate tracking of disability progression must have relatively low variance, i.e., the values shouldn't widely vary from one testing session to another. The test should not be constructed in a way that requires the patient to practice more to perform better; otherwise, the points that fall on this learning curve would have to be removed before analyzing the data.

**Figure 5:**
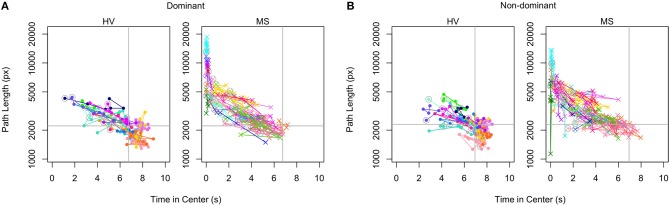
Intra-individual stability plots of Level scores. The path length scores are plotted against the time in center scores for dominant **(A)** and non-dominant **(B)** hands in the longitudinal cohort. Each patient is assigned a distinct color within the HV and MS cohorts. The initial scores of each patient are circled in gray. The median path length and time in center scores of the HV are indicated with gray lines (100% marks) to divide the plots into quadrants. The best scores are in the lower right quadrant and the worst are in the upper left.

To investigate intra-individual stability, we chose to examine path length against time in center data, as these were primary features derived by the application (Figure 5). Each color/shape combination corresponds to a specific patient and is matched across dominant and non-dominant hands. The 100% marks denoted in gray lines indicate the HV median values of both features; values that fall in the upper left quadrant are considered below normal. Within the time-interval available (up to 4 months) we observed no evidence of improvement with time on a group level. While severely-disabled patients had higher variance between individual time-points, the overall performance of the Level Test was intra-individually stable.

## Discussion

In this study, we developed and tested the clinical utility of another simple test of upper extremities neurological functions in our smartphone MS suite, the Level Test, from which we derived five features of putative clinical relevance. By using a digitalized neurological examination, we were able to determine that the Level Test is quite different from previously developed/published smartphone tests of finger tapping (8) and balloon popping (9) because its performance is less dependent on motoric dysfunction (i.e., pyramidal signs and motor fatigue, upper extremities strength and muscle atrophy) and more dependent on cerebellar functions (both hands), proprioception (i.e., vibration in upper extremities for dominant hand) and especially reaction time. Thus, the Level Test fulfilled our goal of diversifying neurological functions necessary for successful performance of individual smartphone tests. This diversification will hopefully allow us to recreate the neurological examination in its entirety by integrating features derived from multiple smartphone tests.

Out of the five Level Test-derived features, time in center and average distance from center outperformed all others in both cross-sectional cohorts (original and validation). However, high correlations between all Level Test features (Supplementary Figure 4), and the inability to develop a composite score that would outperform single best performing feature (i.e., time in center for the non-dominant hand) using statistical learning, indicate that the features we derived measure mostly overlapping neurological functions. Therefore, their integration does not seem to provide additive (or synergistic) value, at least in the cross-sectional comparison of MS patients and HV. We will explore additive value of these features in the longitudinal paradigm once we acquire sufficient longitudinal cohort (i.e., at least 1 year of follow-up).

The observed, reproducible dichotomy between dominant and non-dominant hands in the neurological functions involved in test performance is reminiscent of what was observed previously for simpler smartphone tests such as finger tapping and balloon popping (9) and 9HPT. These cumulative results indicate that the performance of precision movements by the non-dominant hand are more strongly dependent on motoric functions than analogous performance by the dominant hand, where cerebellar and proprioceptive systems may compensate for the motoric dysfunction. This is probably a consequence of compensatory mechanisms evoked by daily usage of the dominant hand irrespective of underlying motoric disability. Consequently, the disability in complex functional tests becomes apparent in the dominant hand only if multiple neurological systems (i.e., motoric, cerebellar, and proprioceptive) are affected. Because this compensatory mechanism lacks on the non-dominant hand side, the majority of functional tests are more sensitive on the non-dominant hand to detect subtle neurological deficits and maximize differences between MS and HV cohorts (8).

The observed strong correlations with cognitive scales SDMT and PASAT emphasize the importance of the reaction time for the successful completion of the Level Test. This interpretation is supported by low correlations between Level Test features and cognitive subdomains of NeurEx (10), which focuses on executive functions and memory rather than the reaction time, which is a strong component of SDMT and PASAT.

We will continue development of additional simple tests of neurological functions, including testing of lower extremities with apps such as foot tapping (8) and walking, to derive an integrated view of neurological disability in individual patients, necessary to achieve our long-term goal of an optimized, data-driven, global outcome that can measure progression of neurological disability in time. Such an outcome will be useful, not only in drug development, but also in monitoring the efficacy of administered treatments in broad clinical practice.

In conclusion, the Level Test is a simple, easily-performed smartphone test that correlates strongly with all traditional MS disability scales, is sensitive to measure differences in disability between MS and HV cohorts, and measures partially distinct neurological domains in comparison to motoric smartphone tests such as finger and foot tapping (8). Only by employing longitudinal data that spans at least 1 year will we be able to determine if the Level Test is sensitive for measuring intra-individual disability progression and whether its integration with other smartphone tests will yield a composite feature(s) with higher clinical value.

## Data Availability

The Level Test datasets for the main cohort and validation cohort with associated data dictionary can be found in Supplementary Files.

## Ethics Statement

This study was carried out in accordance with the recommendations of the Combined Neuroscience Institutional Review Board of the National Institutes of Health (NIH). All subjects gave a written or digital informed consent in accordance with the Declaration of Helsinki. The protocol was approved by the Combined Neuroscience Institutional Review Board.

## Author Contributions

AB executed tests with patients in the clinic and contributed to initial app development and creation of app features. OF contributed to creation of app features and analyzed the features' clinical utility. AW executed tests with patients in the clinic, contributed to creation of app features, and analyzed the features' clinical utility. TH was responsible for app updates and maintenance of the software and cloud-based databases. EK was responsible for the initial prototypes of app development and maintenance of the cloud-based databases. LP executed tests with patients in the clinic and contributed to validation cohort analyses. PK exported and calculated the clinical scores that were used for correlations with Level test scores. BB developed the initial concept of the smartphone test suite containing the Level test in addition to guiding all data analysis and feature development.

### Conflict of Interest Statement

The authors declare that the research was conducted in the absence of any commercial or financial relationships that could be construed as a potential conflict of interest.

## Supplementary Material

Supplementary Video 1 Instructional video for Level Test. This video is available to patients through the testing suite.

Data Sheet 1 Supplementary Tables, Figures and R Scripts.

Data Sheet 2 Level Test datasets for the main cohort and validation cohort with associated data dictionary.
