# Supplementary material for: Smartphone Level Test Measures Disability in Several Neurological Domains for Patients With Multiple Sclerosis
Source: Front Neurol. 2019 May 28;10:358. doi: 10.3389/fneur.2019.00358 (PMC6546929; doi:10.3389/fneur.2019.00358)
Supplement: Data Sheet 1 — Supplementary Tables, Figures and R Scripts. [file Data_Sheet_1.PDF]

Supplementary Material

1. Supplementary Tables

Supplementary Table 1: Demographics for cross-sectional participants in the validation cohort. Patients were split by MS disease subtype into primary progressive MS (PPMS), secondary progressive MS (SPMS), and relapsing remitting MS (RRMS). Data are represented as sample size (%) or mean ± standard deviation, median and range. Percentages do not always sum to 100 due to rounding error. NeurEx is obtained from an app-based documentation of the neurological exam. EDSS denotes Expanded Disability Status Scale. SNRS denotes Scripps Neurologic Rating Scale. CombiWISE denotes Combinatorial Weight-Adjusted Disability Score. SDMT denotes Symbol Digit Modalities Test. MSFC denotes Multiple Sclerosis Functional Composite. 9HPT denotes 9-Hole Peg Test. 25FW denotes 25-Foot Walk Test. PASAT denotes Paced Auditory Serial Addition Test.

	**Cross-sectional MS Validation (N = 29)**			
**Demographics**	**PPMS (n = 15)**	**SPMS (n = 5)**	**RRMS (n = 9)**	**Total (n=29)**
Age - yr				
Mean ± SD	60.1 ± 5.9	61.6 ± 6.6	47.4 ± 10.1	56.4 ± 9.5
Median	60.8	63.7	47.5	58.2
Range	51.2 - 69.2	51.8 - 67.8	31.8 - 67.3	31.8 - 69.2
Gender - no. of patients (%)				
Female	60.0	60.0	66.7	62.1
Male	40.0	40.0	33.3	37.9
**Other clinical characteristics**				
Disease Duration - yr				
Mean ± SD	13.7 ± 8.6	32.5 ± 9.9	5.7 ± 3.7	14.5 ± 11.7
Median	9.3	30.6	4.3	9.2
Range	4.2 - 34.2	23.0 - 49.0	1.6 - 13.5	1.6 - 49.0
NeurEx				
Mean ± SD	165.9 ± 71.5	185.2 ± 101	57.3 ± 21.4	135.5 ± 83.9
Median	174.0	161.0	48.3	115.0
Range	34.2 - 312.0	67.1 - 334	29.8 - 97.2	29.8 - 334.0
EDSS				
Mean ± SD	5.1 ± 1.2	5.5 ± 1.5	3.1 ± 0.9	4.8 ± 1.6
Median	6.0	6.0	3.0	5.0
Range	2.5 - 7	3 - 6.5	2 - 4.5	2.0 - 7.0

	**Cross-sectional MS Validation (N = 29)**			
SNRS				
Mean ± SD	57.3 ± 11.8	55.4 ± 14.7	74.4 ± 4.3	62.3 ± 13.2
Median	59.0	53.0	75.0	63.0
Range	34 - 85	39 - 75	69 - 80	34.0 - 85.0
CombiWISE				
Mean ± SD	45.7 ± 13	45.5 ± 14.4	25.7 ± 4.8	39.5 ± 14.4
Median	44.2	46.6	24.9	39.2
Range	18.6 - 69.1	25.2 - 59.8	18.7 - 33.6	18.6 - 69.1
SDMT[1]				
Mean ± SD	42.9 ± 12.4	38.8 ± 8.1	51.5 ± 13.0	44.6 ± 12.5
Median	46.0	42.0	54.0	46.0
Range	22.0 - 61.0	26.0 - 46.0	27.0 - 68.0	22.0 - 68.0
MSFC				
Mean ± SD	-0.8 ± 2.2	-0.2 ± 0.7	0.2 ± 0.6	-0.3 ± 1.7
Median	0.0	0.3	0.4	0.2
Range	-6.2 - 0.9	-1.1 - 0.4	-0.9 - 0.9	-6.2 - 0.9
9HPT				
Mean ± SD	55.0 ± 101.9	103.8 ± 168.3	21.1 ± 3.4	52.9 ± 100.1
Median	23.2	24.7	20.3	23.6
Range	18.6 - 416.3	23.6 - 404.6	15.8 - 26.2	15.8 - 416.3
25FW				
Mean ± SD	31.8 ± 60.6	8.5 ± 5.3	4.9 ± 1.5	19.4 ± 44.9
Median	6.7	5.7	4.5	5.7
Range	4.0 - 179.9	3.6 - 15.1	3.5 - 8.3	3.5 - 179.9
PASAT[1]				
Mean ± SD	47.3 ± 10.3	53. 8 ± 5	49.1 ± 10.6	49.0 ± 9.7
Median	49.0	54.0	51.0	51.0
Range	24.0 - 60.0	47.0 - 59.0	31.0 - 60.0	24.0 - 60.0

[1]Descriptive statistics were calculated by excluding missing data.

Supplementary Table 2: Coefficients for each Level test feature in the dominant and non-dominant hands. Final coefficients of each Level test feature after averaging the results of 200 simulations of the genetic algorithm (see Methods).

	Path Length	Time in Center	Average Distance from Center	Average Speed in Center	Direction Changes
Dominant	0.003	-0.007	0.003	0.004	0.005
Non-dominant	0.002	-1.946	0.003	0.003	0.003

Supplementary Table 3: Comparison of discriminatory ability for the original (n=93) and validation (n=29) cohorts. Both the original and validation cohorts were able to discriminate between MS patients and healthy volunteers. Within each feature, differences between the scores for MS patients and healthy volunteers were evaluated using a Wilcoxon rank-sum test.

Level Test Feature	Original Cohort Difference in Median Scores (MS – HV)	Validation Cohort Difference in Median Scores (MS – HV)	Original Cohort p-values	Validation Cohort p-values
Dom. Path Length	945 px	446 px	3.89E-03	1.75E-01
Non-Dom. Path Length	1240 px	814 px	8.96E-04	8.31E-02
Dom. Time in Center	-3.45 s	-2.74 s	1.76E-05	3.99E-03
Non-Dom. Time in Center	-3.88 s	-1.85 s	2.16E-07	5.71E-04
Dom. Average Distance from Center	117 px	59.8 px	2.09E-05	4.36E-03
Non-Dom. Average Distance from Center	126 px	75.9 px	8.67E-05	4.13E-04
Dom. Average Speed in Center	73.9 px/s	47.2 px/s	5.33E-03	7.64E-02
Non-Dom. Average Speed in Center	78.2 px/s	9.96 px/s	2.41E-04	1.53E-01
Dom. Direction Changes	4.00	1.75	4.26E-05	4.98E-03
Non-Dom. Direction Changes	3.50	2.50	1.16E-05	2.21E-02

2. Supplementary Figures

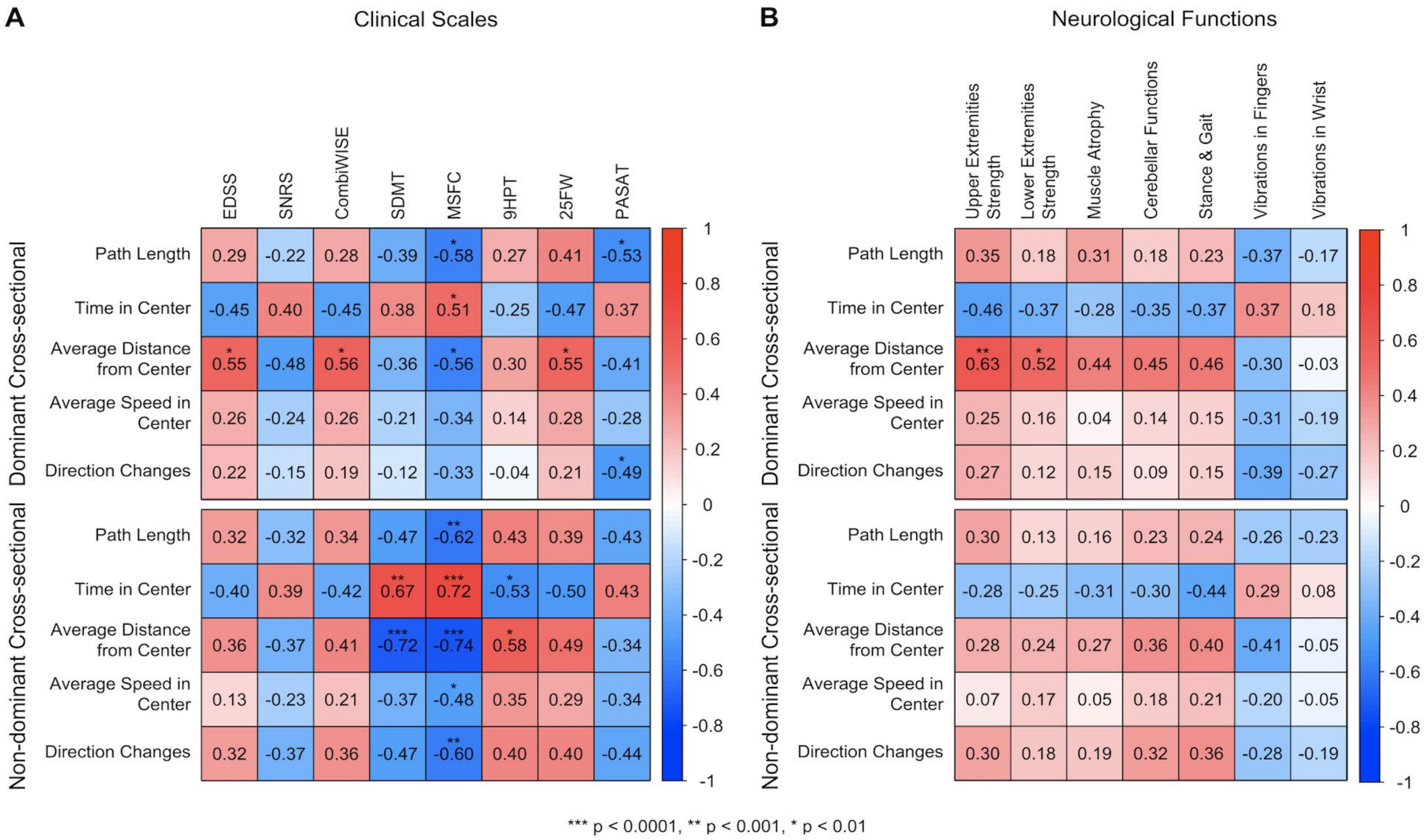

Supplementary Figure 1: (Validation Cohort) Correlations of the Level test features between both clinical scales and neurological functions. **(A)** Spearman correlations between Level test features and eight clinical scales: Expanded Disability Status Scale (EDSS), Scripps Neurological Rating Scale (SNRS), Combinatorial Weight-Adjusted Disability Score (CombiWISE), Symbol Digit Modalities Test (SDMT), Multiple Sclerosis Functional Composite (MSFC), 9-Hole Peg Test (9HPT), 25-Foot Walk (25FW), Paced Auditory Serial Addition Test (PASAT). The 9HPT scores use the dominant/non-dominant hand score. **(B)** Spearman correlations between Level test features and neurological features. The Stance & Gait scores use the entire body score. The following functions use the dominant/non-dominant side score: Upper Extremities Strength, Lower Extremities Strength, Muscle Atrophy, Cerebellar Functions, Vibration in Fingers, and Vibration in Wrist. Red colors correspond to positive correlations, and blue colors correspond to negative correlations. All p-values are adjusted column-wise using false discovery rate and are categorized as *** p < 0.0001, ** p < 0.001, * p < 0.01.

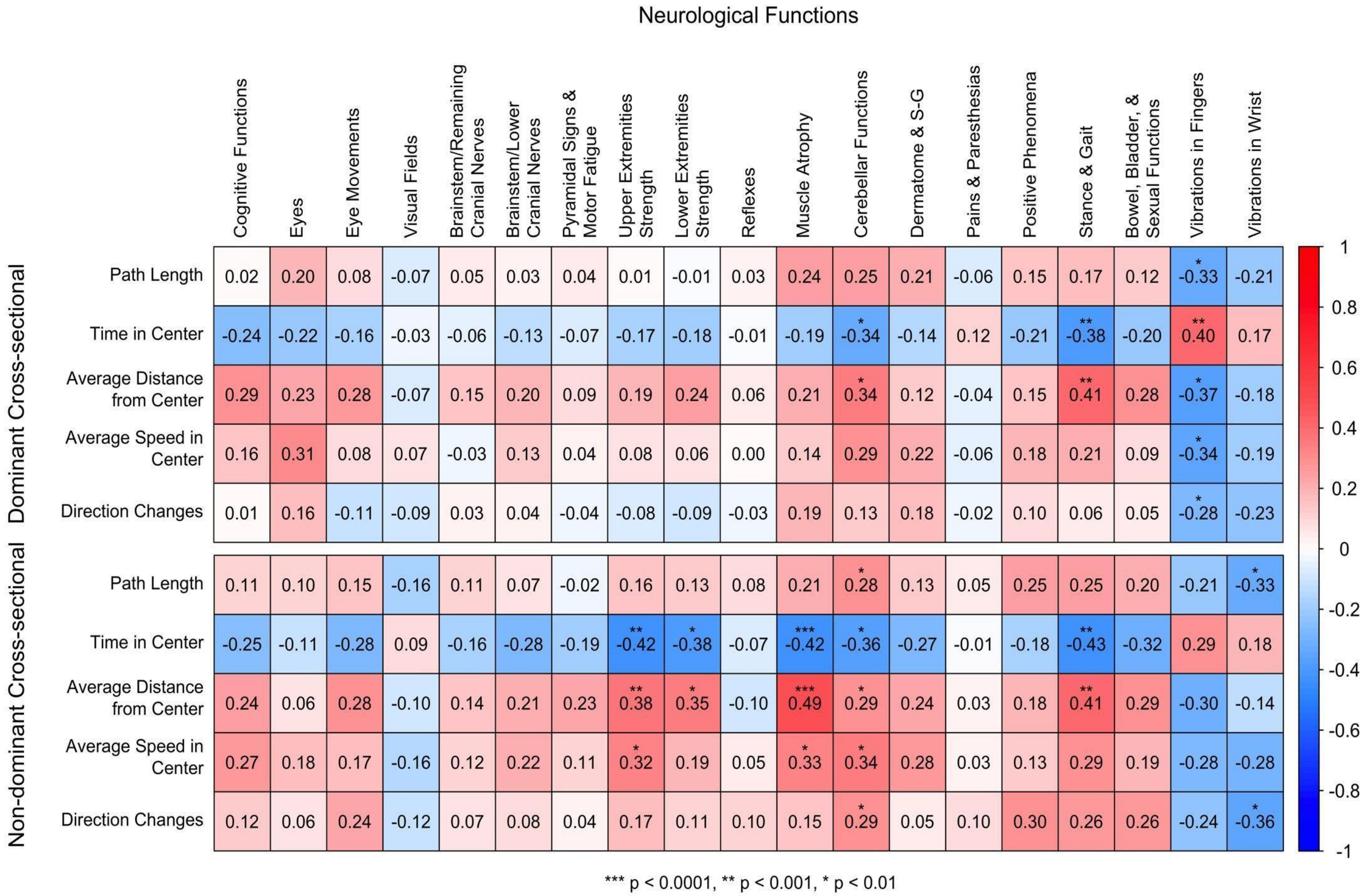

Supplementary Figure 2: (Original Cohort) Spearman correlations between the Level features and neurological functions. The following functions use the entire body score: Cognitive Functions, Eyes, Eye Movements, Visual Fields, Brainstem/Remaining Cranial Nerves, Brainstem/Lower Cranial Nerves, Positive Phenomena, Stance & Gait, and Bowel, Bladder, & Sexual functions. The following functions use the dominant/non-dominant side score: Pyramidal Signs & Motor Fatigue, Upper Extremities Strength, Lower Extremities Strength, Reflexes, Muscle Atrophy, Cerebellar Functions, Dermatone & S-G, Pains & Paresthesias, Vibration in Fingers, and Vibration in Wrist. Red colors correspond to positive correlations, and blue colors correspond to negative correlations. The p-values are adjusted column-wise using false discovery rate and are categorized as *** p < 0.0001, ** p < 0.001, * p < 0.01.

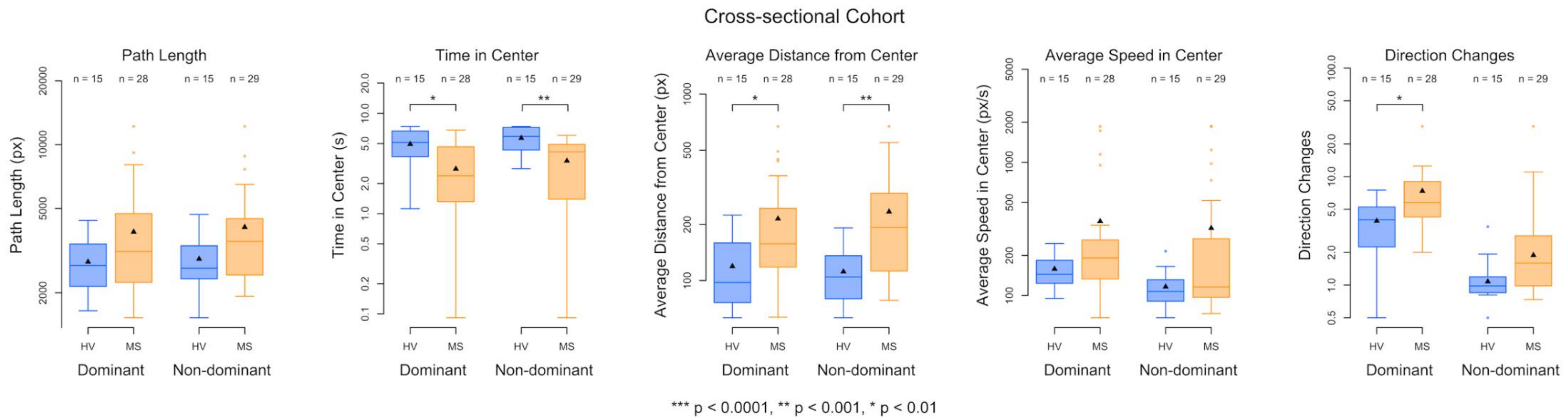

Supplementary Figure 3: (Validation Cohort) Boxplots of the Level features separated by dominant/non-dominant hand for the MS (validation) and HV cohorts. Cross-sectional data shown consists of the average of the two trials from the baseline visit. Differences between the groups were determined using a Wilcoxon rank-sum test with *** $p < 0.0001$, ** $p < 0.001$, * $p < 0.01$. Lower scores for path length, average distance from center, average speed in center, and direction changes correspond to better performance, while higher scores for time in center correspond to better performance.

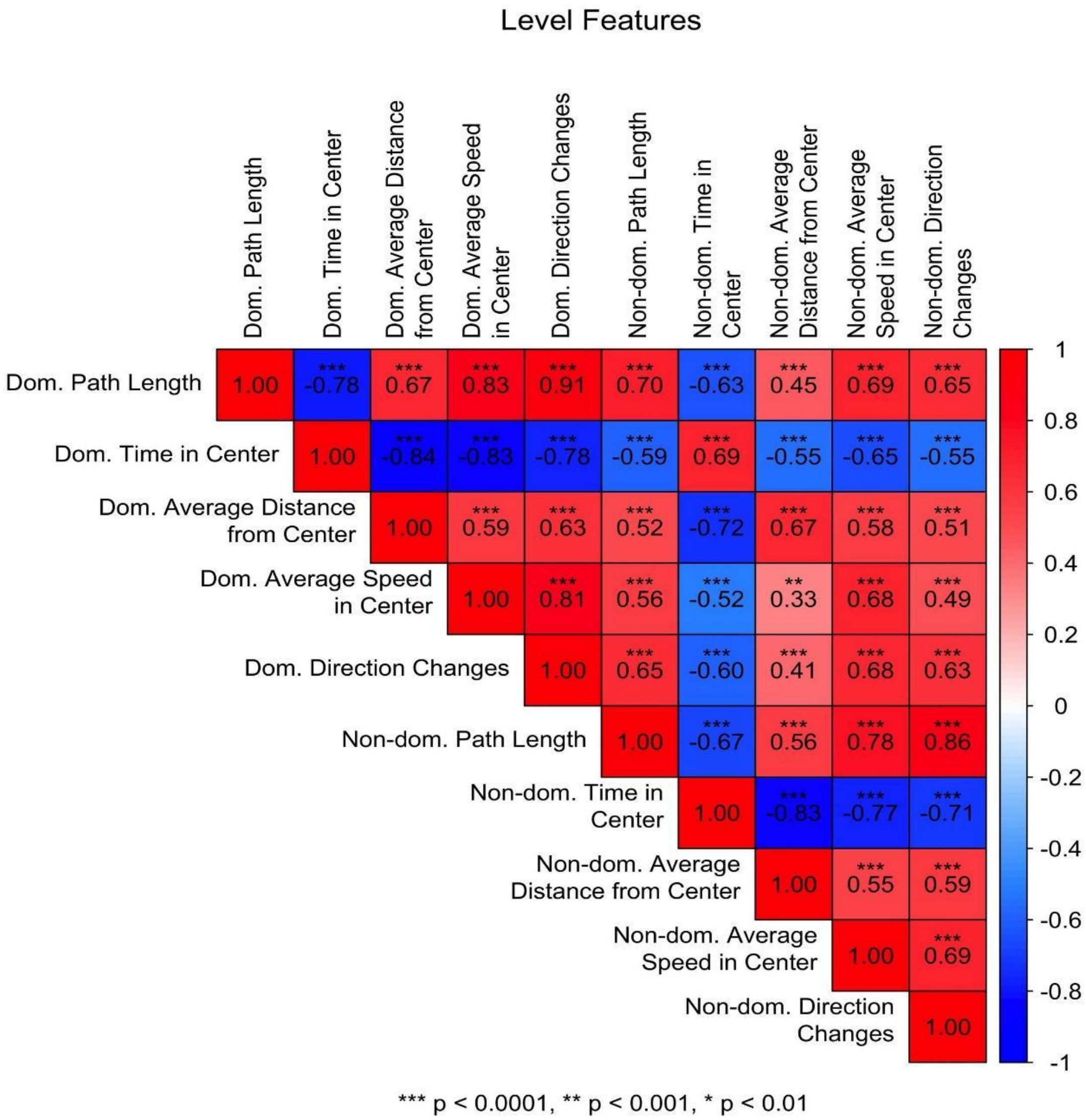

Supplementary Figure 4: (Original Cohort) Spearman correlations between Level features in the dominant and non-dominant hands. Red colors correspond to positive correlations, and blue colors correspond to negative correlations. The p-values are adjusted column-wise using false discovery rate, and are categorized as *** $p < 0.0001$, ** $p < 0.001$, * $p < 0.01$.

3. R Scripts

```
# ---------------------------------------------------------------------------
# Olivia Fan
# Created 02/25/2018
# Edited 07/02/2018
# 01 Setup
# This script reads the data, formats the timepoints, splits the data
# according to whether the hand is dominant or non-dominant, and then saves
# two new spreadsheets with dominant and non-dominant hand data.
# ---------------------------------------------------------------------------

# ---------------------------------------------------------------------------
# Read data
# ---------------------------------------------------------------------------

readData <- function(filename) {
  data <- read.csv(filename, header = TRUE, na.strings = c("", "NA"))
  return(data)
}

LH_all <- readData("/Users/oliviafan/Documents/Research/NIH 2018/Level
Test/Data/Original Spreadsheets/LevelTest_LH.csv")
RH_all <- readData("/Users/oliviafan/Documents/Research/NIH 2018/Level
Test/Data/Original Spreadsheets/LevelTest_RH.csv")
dom_hand_key <- readData("/Users/oliviafan/Documents/Research/NIH 2018/Level
Test/Data/Original Spreadsheets/Handedness.csv")
# Remove empty rows
LH_all <- LH_all[rowSums(is.na(LH_all)) < ncol(LH_all),]
RH_all <- RH_all[rowSums(is.na(RH_all)) < ncol(RH_all),]
# Remove trials with impossible times in center
LH_all <- LH_all[LH_all$Time.Spent.in.Cen...ms. < 10000,]
```

```r
RH_all <- RH_all[RH_all$Time.Spent.in.Cen...ms. < 10000,]
# Only keep test difficulty 3
LH_all <- LH_all[LH_all$Test.Dfclty == 2,]
RH_all <- RH_all[RH_all$Test.Dfclty == 2,]

# ---------------------------------------------------------------------------
# Format timepoints
# ---------------------------------------------------------------------------

# Constant
TIMEPTS_START <- 9

# Function to strip all but numeric characters away from a cell
stripPoint <- function(point) {
  return(gsub("[^0-9\\.]", " ", point))
}

# Function to split the strings at the empty spaces and remove those trailing spaces (\\s+)
cleanPoint <- function(point) {
  return(as.numeric(strsplit(point, "\\s+")[[1]][-1]))
}

# Function to format each row
formatRow <- function(row_orig) {
  row_orig <- row_orig[, colSums(is.na(row_orig)) < nrow(row_orig)] # Get rid of empty
columns
  row_clean <- mapply(function(i) {
    cell <- row_orig[1, i]
    cell <- stripPoint(cell)
    cell <- cleanPoint(cell)
    return(cell)
```

```r
  }, TIMEPTS_START:ncol(row_orig))
  row_clean <- as.vector(row_clean)
  return(row_clean)
}

# Function to format the timepoints
formatTimepoints <- function(data) {
  data_clean <- sapply(1:nrow(data), function(i) {
    row_orig <- data[i, ]
    row_orig <- row_orig[, colSums(is.na(row_orig)) < nrow(row_orig)] # Get rid of empty
columns
    row_clean <- formatRow(row_orig)
    return(row_clean)
  })
}

library(plyr)

# Format the overall data
LH_all_timepoints <- ldply(formatTimepoints(LH_all), rbind)
LH_all <- cbind(LH_all[, 1:TIMEPTS_START-1], LH_all_timepoints)
RH_all_timepoints <- plyr::ldply(formatTimepoints(RH_all), rbind)
RH_all <- cbind(RH_all[, 1:TIMEPTS_START-1], RH_all_timepoints)

# ---------------------------------------------------------------------------
# Add dominant hand to the data
# ---------------------------------------------------------------------------

# Map of PID to dominant hand
dom_hand_key <- unique(dom_hand_key)
```

```
# Function to get the dominant hand for each row in the data set
getDomHand <- function(data) {
  # Create a vector with the dominant hands for each line
  dom_hand <- c()
  lapply(1:nrow(data), function(i) {
    PID <- as.character(data[i, 2])
    dom_hand <- c(dom_hand,
as.character(dom_hand_key[dom_hand_key$PatientCode==PID, "Dominant.Hand"]))
  })
}

# Add the dominant hand to the data
LH_all_domhand <- ldply(getDomHand(LH_all), rbind)
LH_all <- data.frame(LH_all[, 1:2], LH_all_domhand,
                     LH_all[, 3:ncol(LH_all)])
RH_all_domhand<- ldply(getDomHand(RH_all), rbind)
RH_all <- data.frame(RH_all[, 1:2], RH_all_domhand,
                     RH_all[, 3:ncol(RH_all)])
LH_all <- LH_all[, -4]
RH_all <- RH_all[, -4]


# Rename
names(LH_all) <- c("Group", "PID", "Dom_Hand", "Test_Date", "App_Ver",
                   "Test_Dif", "Path_Length_(mm)", "Time_Center_(ms)",
                   "Avg_Displace_(px)")
rownames(LH_all) <- c()
names(RH_all) <- c("Group", "PID", "Dom_Hand", "Test_Date", "App_Ver",
                   "Test_Dif", "Path_Length_(mm)", "Time_Center_(ms)",
                   "Avg_Displace_(px)")
rownames(RH_all) <- c()
```

```r
# ---------------------------------------------------------------------------
# Split the data into dominant and non dominant hands
# ---------------------------------------------------------------------------

LH_dom <- subset(LH_all, LH_all$Dom_Hand == "Left")
LH_ndom <- subset(LH_all, LH_all$Dom_Hand == "Right")
RH_dom <- subset(RH_all, RH_all$Dom_Hand == "Right")
RH_ndom <- subset(RH_all, RH_all$Dom_Hand == "Left")

# ---------------------------------------------------------------------------
# Export dominant and non-dominant spreadsheets
# ---------------------------------------------------------------------------

# Dominant
write.table(LH_dom,
        file = "/Users/oliviafan/Documents/Research/NIH 2018/Level Test/Data/Edited
Spreadsheets/01_Setup_Dom_2.csv",
        sep = ",", row.names = F)
write.table(RH_dom,
        file = "/Users/oliviafan/Documents/Research/NIH 2018/Level Test/Data/Edited
Spreadsheets/01_Setup_Dom_2.csv",
        sep = ",", row.names = F, col.names = F, append = T)

# Non-dominant
write.table(LH_ndom,
        file = "/Users/oliviafan/Documents/Research/NIH 2018/Level Test/Data/Edited
Spreadsheets/01_Setup_Nondom_2.csv",
        sep = ",", row.names = FALSE)
write.table(RH_ndom,
        file = "/Users/oliviafan/Documents/Research/NIH 2018/Level Test/Data/Edited
```

```
Spreadsheets/01_Setup_Nondom_2.csv",
        sep = ",", row.names = FALSE, col.names = FALSE, append = TRUE)
```

```r
# ---------------------------------------------------------------------------
# Olivia Fan
# Created 07/20/2018
# Edited 07/23/2018
# 02 Remove Outliers
# This script removes the outliers.
# ---------------------------------------------------------------------------

# ---------------------------------------------------------------------------
# Read data
# ---------------------------------------------------------------------------

readData <- function(filename) {
  data <- read.csv(filename, header = TRUE, na.strings = c("", "NA"))
  return(data)
}
dom_all <- readData("/Users/oliviafan/Documents/Research/NIH 2018/Level Test/Data/Edited
Spreadsheets/01_Setup_Dom.csv")
ndom_all <- readData("/Users/oliviafan/Documents/Research/NIH 2018/Level
Test/Data/Edited Spreadsheets/01_Setup_Nondom.csv")

# ---------------------------------------------------------------------------
# Split the date and time
# ---------------------------------------------------------------------------

TIMEPTS_START <- 10

# Function to split the strings at the empty spaces and remove those trailing spaces (\\s+)
cleanPoint <- function(point) {
  return(strsplit(point, " ")[[1]])
}
```

```
# Function to change the date and time into two columns
splitDateTime <- function(data) {
  data1 <- data[, 1:3]
  data2 <- data[, 5:ncol(data)]
  date_time <- data[, 4]
  date_time <- data.frame(date_time)
  date_time_new <- sapply(1:nrow(date_time), function(x) {
    return(cleanPoint(as.character(date_time[x, 1])))
  })
  date_time_new <- data.frame(t(date_time_new))
  names(date_time_new) <- c("Date", "Time")
  date_time_new$Date <- as.factor(date_time_new$Date)
  data <- cbind(data1, date_time_new, data2)
}

# Modify the data sheets by splitting the date and time into separate columns
dom_all <- splitDateTime(dom_all)
ndom_all <- splitDateTime(ndom_all)

# ---------------------------------------------------------------------------
# Remove the longitudinal outliers
# ---------------------------------------------------------------------------

# Split the data by patient
dom_all <- split(dom_all, dom_all$PID)
ndom_all <- split(ndom_all, ndom_all$PID)

# Function to remove the outliers within one individual's trials based on
# path length and time in center
removeOutliersIndiv <- function(data) {
```

```
  # Reveal how many total trials
  #print(paste("Total trials:", nrow(data)))
  # Path length outliers
  data_measure_pl <- as.numeric(data[, 8])
  data_measure_pl_fence <- quantile(data_measure_pl)[4]+1.5*(quantile(data_measure_pl)[4]-
quantile(data_measure_pl)[2])
  outlier_pl_indices <- which(data_measure_pl > data_measure_pl_fence)
  # Time in center outliers
  data_measure_tc <- as.numeric(data[, 9])
  data_measure_tc_fence <- quantile(data_measure_tc)[2]-1.5*(quantile(data_measure_tc)[4]-
quantile(data_measure_tc)[2])
  outlier_tc_indices <- which(data_measure_tc < data_measure_tc_fence)
  # All outlier indices
  outlier_indices <- c(outlier_pl_indices, outlier_tc_indices)
  outlier_indices <- unique(outlier_indices)
  # Remove outliers
  if (length(outlier_indices)>0) {
    data_no_outliers <- data[-outlier_indices,]
  }
  else {
    data_no_outliers <- data
  }
  # Reveal how many outliers were excluded
  #print(paste("Removed trials:", length(outlier_indices)))
  # Return data without outliers
  return(data_no_outliers)
}

# Function to remove all the patients' outliers
removeLongOutliers <- function(data) {
  lapply(1:length(data), function(x) {
```

```
    data_no_outliers <- removeOutliersIndiv(data[[x]])
  })
}

# Reorganize the data
library(plyr)
dom_all_no_long_outliers <- removeLongOutliers(dom_all)
dom_all_no_long_outliers <- ldply(dom_all_no_long_outliers, data.frame)
ndom_all_no_long_outliers <- removeLongOutliers(ndom_all)
ndom_all_no_long_outliers <- ldply(ndom_all_no_long_outliers, data.frame)

# ---------------------------------------------------------------------------
# Remove the cross-sectional outliers
# ---------------------------------------------------------------------------

# Function to remove the first trial of a patient if path length is twice as
# long as the second, or if time in center is half as short as the second
removeOutliersDate <- function(data) {
  remove = F
  data$Date <- as.character(data$Date)
  data <- split(data, data$Date)
  data1 <- data[[1]]
  if (nrow(data1) > 1) {
    # Path length
    data_measure_pl_1 <- data1[1, 8]
    data_measure_pl_2 <- data1[2, 8]
    if (data_measure_pl_1 > 2*data_measure_pl_2) {
      remove = T
    }
    # Time in center
    data_measure_tc_1 <- data1[1, 9]
```

```
    data_measure_tc_2 <- data1[2, 9]
    if (2*data_measure_tc_1 < data_measure_tc_2) {
      remove = T
    }
    # Remove bad first trial
    if (remove) {
      data1 <- data1[-1,]
    }
    data[[1]] <- data1
  }
  # Return data without outliers
  data <- ldply(data, data.frame)
  return(data)
}

# Function to remove all the bad first trials
removeXsecOutliers <- function(data) {
  lapply(1:length(data), function(x) {
    data_new <- removeOutliersDate(data[[x]])
  })
}

# Function to divide the data by patient and by date
splitData <- function(data) {
  data$PID <- as.character(data$PID)
  data <- split(data, data$PID)
  return(data)
}

dom_all_no_outliers <- splitData(dom_all_no_long_outliers)
ndom_all_no_outliers <- splitData(ndom_all_no_long_outliers)
```

```
# Reorganize the data
dom_all_no_outliers <- removeXsecOutliers(dom_all_no_outliers)
dom_all_no_outliers <- ldply(dom_all_no_outliers, data.frame)
ndom_all_no_outliers <- removeXsecOutliers(ndom_all_no_outliers)
ndom_all_no_outliers <- ldply(ndom_all_no_outliers, data.frame)
dom_all_no_outliers <- dom_all_no_outliers[, -1]
ndom_all_no_outliers <- ndom_all_no_outliers[, -1]

# ---------------------------------------------------------------------------
# Export modified data sheets
# ---------------------------------------------------------------------------

write.table(dom_all_no_outliers,
	file="/Users/oliviafan/Documents/Research/NIH 2018/Level Test/Data/Edited
Spreadsheets/02_Dom_All.csv",
	sep=",", row.names=F)
write.table(ndom_all_no_outliers,
	file="/Users/oliviafan/Documents/Research/NIH 2018/Level Test/Data/Edited
Spreadsheets/02_Nondom_All.csv",
	sep=",", row.names=F)
```

```
# ---------------------------------------------------------------------------
# Olivia Fan
# Created 02/25/2018
# Edited 07/20/2018
# 03 Split Xsec and Long
# This script splits the dom_all and ndom_all sheets into xsec and long.
# ---------------------------------------------------------------------------

# ---------------------------------------------------------------------------
# Read data
# ---------------------------------------------------------------------------

readData <- function(filename) {
  data <- read.csv(filename, header = TRUE, na.strings = c("", "NA"))
  return(data)
}
dom_all <- readData("/Users/oliviafan/Documents/Research/NIH 2018/Level Test/Data/Edited
Spreadsheets/02_Dom_All.csv")
ndom_all <- readData("/Users/oliviafan/Documents/Research/NIH 2018/Level
Test/Data/Edited Spreadsheets/02_Nondom_All.csv")

# ---------------------------------------------------------------------------
# Split data in cross sectional and longitudinal
# ---------------------------------------------------------------------------

library(plyr)
require(lubridate)

# Function to get the cross sectional data
getXsec <- function(data) {
  PID = unique(as.character(data$PID))
```

```
  data_patients_all <- split(data, data$PID)
  data_xsec <- data.frame()
  lapply(1:length(PID), function(i) {
    data_patient <- data_patients_all[[i]]
    data_patient$Date <- as.Date(mdy(as.character(data_patient$Date)))
    data_patient <- data_patient[order(data_patient$Date),]
    data_patient <- split(data_patient, data_patient$Date)
    data_patient_xsec <- data_patient[[1]]
    data_xsec <- rbind(data_xsec, data_patient_xsec)
  })
}

# Dom cross sectional data
dom_xsec <- getXsec(dom_all)
dom_xsec <- do.call(rbind.data.frame, dom_xsec)
dom_xsec <- dom_xsec[rowSums(is.na(dom_xsec)) != ncol(dom_xsec),]
# Ndom cross sectional data
ndom_xsec <- getXsec(ndom_all)
ndom_xsec <- do.call(rbind.data.frame, ndom_xsec)
ndom_xsec <- ndom_xsec[rowSums(is.na(ndom_xsec)) != ncol(ndom_xsec),]

# Function to get the longitudinal data
getLong <- function(data) {
  data_patients_all <- split(data, data$PID)
  PID = unique(data$PID)
  data_long <- data.frame()
  lapply(1:length(PID), function(i) {
    data_patient <- data_patients_all[[i]]
    data_patient$Date <- as.Date(mdy(as.character(data_patient$Date)))
    data_patient_dates <- length(data_patient$Date)
    if (data_patient_dates >= 3) {
```

```
      data_long <- rbind(data_long, data_patient)
    }
  })
}

# Dom longitudinal data
dom_long <- getLong(dom_all)
dom_long <- do.call(rbind.data.frame, dom_long)
dom_long <- dom_long[rowSums(is.na(dom_long)) != ncol(dom_long)]
# Ndom longitudinal data
ndom_long <- getLong(ndom_all)
ndom_long <- do.call(rbind.data.frame, ndom_long)
ndom_long <- ndom_long[rowSums(is.na(ndom_long)) != ncol(ndom_long)]

# -------------------------------------------------------------------------
# Export LH_all and RH_all
# -------------------------------------------------------------------------

write.table(dom_xsec,
          file="/Users/oliviafan/Documents/Research/NIH 2018/Level Test/Data/Edited
Spreadsheets/03_Dom_Xsec.csv",
          sep=",", row.names=F)
write.table(dom_long,
          file="/Users/oliviafan/Documents/Research/NIH 2018/Level Test/Data/Edited
Spreadsheets/03_Dom_Long.csv",
          sep=",", row.names=F)
write.table(ndom_xsec,
          file="/Users/oliviafan/Documents/Research/NIH 2018/Level Test/Data/Edited
Spreadsheets/03_Nondom_Xsec.csv",
          sep=",", row.names=F)
write.table(ndom_long,
```

```
        file="/Users/oliviafan/Documents/Research/NIH 2018/Level Test/Data/Edited Spreadsheets/03_Nondom_Long.csv",
        sep=",", row.names=F)
```

```
# ---------------------------------------------------------------------------
# Olivia Fan
# Created 02/25/2018
# Edited 07/20/2018
# 04 Velocities
# This script calculates the overall velocity of the ball and the velocity of
# the ball in the center within 90 px, and adds the two to the spreadsheet.
# ---------------------------------------------------------------------------

# ---------------------------------------------------------------------------
# Read data
# ---------------------------------------------------------------------------

readData <- function(filename) {
  data <- read.csv(filename, header = TRUE, na.strings = c("", "NA"))
  data <- data[rowSums(is.na(data)) != ncol(data),] # Remove empty rows
  return(data)
}
dom_xsec <- readData("/Users/oliviafan/Documents/Research/NIH 2018/Level
Test/Data/Edited Spreadsheets/03_Dom_Xsec.csv")
dom_long <- readData("/Users/oliviafan/Documents/Research/NIH 2018/Level
Test/Data/Edited Spreadsheets/03_Dom_Long.csv")
ndom_xsec <- readData("/Users/oliviafan/Documents/Research/NIH 2018/Level
Test/Data/Edited Spreadsheets/03_Nondom_Xsec.csv")
ndom_long <- readData("/Users/oliviafan/Documents/Research/NIH 2018/Level
Test/Data/Edited Spreadsheets/03_Nondom_Long.csv")

# ---------------------------------------------------------------------------
# Add the overall avg vel and center avg vel to the data
# ---------------------------------------------------------------------------
```

```r
# Helper function to get the Euclidean distance
getEuclidDistance <- function(x1, y1, x2, y2) {
  dist <- sqrt((x1-x2)^2+(y1-y2)^2)
}

TIMEPTS_START <- 11

# Function to get the distances in one row
getDist <- function(data, i) {
  row <- data[i,]
  row <- row[, colSums(is.na(row)) < nrow(row)] # Get rid of empty columns
  row <- as.numeric(row) # Converts to a vector
  dist_vector <- sapply(1:((length(row)-TIMEPTS_START-1)/3), function(j) {
    x1 <- row[3*j+TIMEPTS_START]
    y1 <- row[3*j+TIMEPTS_START+1]
    x2 <- row[3*(j-1)+TIMEPTS_START]
    y2 <- row[3*(j-1)+TIMEPTS_START+1]
    dist <- getEuclidDistance(x1, y1, x2, y2)
    return(dist)
  })
}

# Function to get the times in one row
getTime <- function(data, i) {
  row <- data[i,]
  row <- row[, colSums(is.na(row)) < nrow(row)] # Get rid of empty columns
  row <- as.numeric(row) # Converts to a vector
  time_vector <- sapply(1:((length(row)-TIMEPTS_START-1)/3), function(j) {
    t1 <- row[3*j+TIMEPTS_START+2]
    t2 <- row[3*(j-1)+TIMEPTS_START+2]
    time <- t1-t2
```

```
    if (time > 10000000)
      return(time)
    else
      return(16000000)
  })
}

# Function to get the overall average velocities
getVelOverall <- function(data) {
  vel_overall <- sapply(1:nrow(data), function(i) {
    row <- data[i,]
    row <- row[, colSums(is.na(row)) < nrow(row)] # Get rid of empty columns
    row <- as.numeric(row) # Converts to a vector
    dist <- unlist(getDist(data, i))*1000000000
    time <- unlist(getTime(data, i))
    return(sum(dist)/sum(time))
  })
}

# Function to get whether points are in the center
getVelInclude <- function(data, i) {
  row <- data[i,]
  row <- row[, colSums(is.na(row)) < nrow(row)] # Get rid of empty columns
  row <- as.numeric(row) # Converts to a vector
  vel_include <- sapply(1:((length(row)-TIMEPTS_START-1)/3), function(j) {
    x1 <- row[3*j+TIMEPTS_START]
    y1 <- row[3*j+TIMEPTS_START+1]
    dist1 <- getEuclidDistance(x1, y1, 720, 976)
    x2 <- row[3*(j-1)+TIMEPTS_START]
    y2 <- row[3*(j-1)+TIMEPTS_START+1]
    dist2 <- getEuclidDistance(x2, y2, 720, 976)
```

```
    in_center <- dist1 < 45 & dist2 < 45
    return(in_center)
  })
}

# Function to get the average velocities within 90 px of the center (720, 976)
getVelCenter <- function(data) {
  vel_center <- sapply(1:nrow(data), function(i) {
    row <- data[i,]
    row <- row[, colSums(is.na(row)) < nrow(row)] # Get rid of empty columns
    row <- as.numeric(row) # Converts to a vector
    dist <- unlist(getDist(data, i))*1000000000
    time <- unlist(getTime(data, i))
    dist_center <- dist[getVelInclude(data, i)]
    time_center <- time[getVelInclude(data, i)]
    vel_center <- sum(dist_center)/sum(time_center)
    # Set fail code to be 3000 px/s
    if (is.na(vel_center)) {
      vel_center <- 3000
    }
    return(vel_center)
  })
}

# Add the overall velocities and velocities in center to the data sheets right before the
timepoints
dom_xsec <- data.frame(dom_xsec[, 1:TIMEPTS_START-1],
                       data.frame(getVelOverall(dom_xsec)),
                       data.frame(getVelCenter(dom_xsec)),
                       dom_xsec[, TIMEPTS_START:ncol(dom_xsec)])
dom_long <- data.frame(dom_long[, 1:TIMEPTS_START-1],
```

```
                        data.frame(getVelOverall(dom_long)),
                        data.frame(getVelCenter(dom_long)),
                        dom_long[, TIMEPTS_START:ncol(dom_long)])
ndom_xsec <- data.frame(ndom_xsec[, 1:TIMEPTS_START-1],
                         data.frame(getVelOverall(ndom_xsec)),
                         data.frame(getVelCenter(ndom_xsec)),
                         ndom_xsec[, TIMEPTS_START:ncol(ndom_xsec)])
ndom_long <- data.frame(ndom_long[, 1:TIMEPTS_START-1],
                         data.frame(getVelOverall(ndom_long)),
                         data.frame(getVelCenter(ndom_long)),
                         ndom_long[, TIMEPTS_START:ncol(ndom_long)])

# Rename headers
header_names <- c("Group", "PID", "Dom_Hand", "Date", "Time", "App_Ver",
                  "Test_Dif", "Path_Length_(mm)", "Time_Center_(ms)",
                  "Avg_Displace_(px)", "Vel_Overall_(px/sec)",
                  "Vel_Center_(px/sec)")
names(dom_xsec) <- header_names
names(dom_long) <- header_names
names(ndom_xsec) <- header_names
names(ndom_long) <- header_names

# ---------------------------------------------------------------------------
# Export data sheets with added overall velocity and velocity in center
# ---------------------------------------------------------------------------

write.table(dom_xsec,
            file="/Users/oliviafan/Documents/Research/NIH 2018/Level Test/Data/Edited
Spreadsheets/04_Dom_Xsec.csv",
            sep=",", row.names=F)
write.table(dom_long,
```

```
	file="/Users/oliviafan/Documents/Research/NIH 2018/Level Test/Data/Edited Spreadsheets/04_Dom_Long.csv",
	sep=",", row.names=F)
write.table(ndom_xsec,
	file="/Users/oliviafan/Documents/Research/NIH 2018/Level Test/Data/Edited Spreadsheets/04_Nondom_Xsec.csv",
	sep=",", row.names=F)
write.table(ndom_long,
	file="/Users/oliviafan/Documents/Research/NIH 2018/Level Test/Data/Edited Spreadsheets/04_Nondom_Long.csv",
	sep=",", row.names=F)
```

```r
# ---------------------------------------------------------------------------
# Olivia Fan
# Created 03/02/2018
# Edited 07/20/2018
# 05 Amplitude and Frequency
# This script calculates the "amplitude and "frequency" of each trial.
# Amplitude: total points where you add n points when you cross the (n+1)th
# concentric circle.
# Frequency: the number of times the tester crosses the border of the center
# area, radius of center is 45 for test dif. 3, 90 for test dif. 2, and 135
# for test dif. 1.
# ---------------------------------------------------------------------------

# ---------------------------------------------------------------------------
# Read data
# ---------------------------------------------------------------------------

readData <- function(filename) {
  data <- read.csv(filename, header = TRUE, na.strings = c("", "NA"))
  return(data)
}

dom_xsec <- readData("/Users/oliviafan/Documents/Research/NIH 2018/Level
Test/Data/Edited Spreadsheets/04_Dom_Xsec.csv")
ndom_xsec <- readData("/Users/oliviafan/Documents/Research/NIH 2018/Level
Test/Data/Edited Spreadsheets/04_Nondom_Xsec.csv")
dom_long <- readData("/Users/oliviafan/Documents/Research/NIH 2018/Level
Test/Data/Edited Spreadsheets/04_Dom_Long.csv")
ndom_long <- readData("/Users/oliviafan/Documents/Research/NIH 2018/Level
Test/Data/Edited Spreadsheets/04_Nondom_Long.csv")
```

```
# ---------------------------------------------------------------------------
# Determine the amplitude and frequency of all trials
# ---------------------------------------------------------------------------

# Helper function to get the distance from the center (720, 976)
getDistCenter <- function(x1, y1) {
  dist <- sqrt((x1-720)^2+(y1-976)^2)
}

TIMEPTS_START = 13
r = 45

# Function to get the distances from the center in one row
getDistRow <- function(data, i) {
  row <- data[i,]
  row <- as.numeric(row) # Converts to a vector
  dist_vector <- sapply(1:((length(row)-TIMEPTS_START+1)/3), function(j) {
    x1 <- row[3*(j-1)+TIMEPTS_START]
    y1 <- row[3*(j-1)+TIMEPTS_START+1]
    dist <- getDistCenter(x1, y1)
    return(dist)
  })
}

# Function to check whether two distances passed a circle (r = 45)
# If so, which circle, e.g. distance passed = 45n, find n
# If no circle passed, returns 0
getPassed <- function(dist1, dist2) {
  if (dist1 < dist2) {
    range_dist <- ceiling(dist1):dist2
  }
```

```
  else {
    range_dist <- ceiling(dist2):dist1
  }
  n = 0
  for (x in range_dist) {
    if (x %% r == 0)
      n = x/r
  }
  return(n)
}

# Function to get passed circles in a row
getPassedRow <- function(data, i) {
  row_dist <- getDistRow(data, i)
  passed_vector <- sapply(2:length(row_dist), function(j) {
    dist1 <- row_dist[j-1]
    dist2 <- row_dist[j]
    if (!is.na(dist1) & !is.na(dist2)) {
      n <- getPassed(dist1, dist2)
      return(n)
    }
    else {
      return(0)
    }
  })
}

# Function to get all of the distances from center, check if they passed a circle, if so which one
getCirclesPassed <- function(data) {
  circles_passed <- sapply(1:nrow(data), function(i) {
    return(getPassedRow(data, i))
```

```r
  })
}

# Function to get the amplitude
getAmplitude <- function(data) {
  data_circles_passed <- t(getCirclesPassed(data))
  amplitude <- rowSums(data_circles_passed) - rowSums(data_circles_passed != 0)
  return(amplitude)
}

# Function to get the frequency
getFrequency <- function(data) {
  test_dif <- data$Test_Dif
  frequency <- sapply(1:nrow(data), function(i) {
    n <- 4 - test_dif[i]
    row_circles_passed <- getPassedRow(data, i)
    freq <- sum(row_circles_passed == n)
    # Set frequency of 0 to 50, to represent a failed trial
    if (freq == 0) {
      freq <- 50
    }
    return(freq)
  })
}

# Amplitudes
dom_xsec_amp <- getAmplitude(dom_xsec)
ndom_xsec_amp <- getAmplitude(ndom_xsec)
dom_long_amp <- getAmplitude(dom_long)
ndom_long_amp <- getAmplitude(ndom_long)
```

```
# Frequencies
dom_xsec_freq <- getFrequency(dom_xsec)
ndom_xsec_freq <- getFrequency(ndom_xsec)
dom_long_freq <- getFrequency(dom_long)
ndom_long_freq <- getFrequency(ndom_long)

# Add amplitude and frequency to the data
dom_xsec <- data.frame(dom_xsec[, 1:TIMEPTS_START-1],
                       data.frame(dom_xsec_amp),
                       data.frame(dom_xsec_freq),
                       dom_xsec[, TIMEPTS_START:ncol(dom_xsec)])
ndom_xsec <- data.frame(ndom_xsec[, 1:TIMEPTS_START-1],
                        data.frame(ndom_xsec_amp),
                        data.frame(ndom_xsec_freq),
                        ndom_xsec[, TIMEPTS_START:ncol(ndom_xsec)])
dom_long <- data.frame(dom_long[, 1:TIMEPTS_START-1],
                       data.frame(dom_long_amp),
                       data.frame(dom_long_freq),
                       dom_long[, TIMEPTS_START:ncol(dom_long)])
ndom_long <- data.frame(ndom_long[, 1:TIMEPTS_START-1],
                        data.frame(ndom_long_amp),
                        data.frame(ndom_long_freq),
                        ndom_long[, TIMEPTS_START:ncol(ndom_long)])

# Rename headers
header_names <- c("Group", "PID", "Dom_Hand", "Date", "Time", "App_Ver",
                  "Test_Dif", "Path_Length_(mm)", "Time_Center_(ms)",
                  "Avg_Displace_(px)", "Vel_Overall_(px/sec)",
                  "Vel_Center_(px/sec)", "Amplitude", "Frequency")
names(dom_xsec) <- header_names
names(dom_long) <- header_names
```

```
names(ndom_xsec) <- header_names
names(ndom_long) <- header_names

# --------------------------------------------------------------------------
# Export modified data sheets
# --------------------------------------------------------------------------

write.table(dom_xsec,
	file="/Users/oliviafan/Documents/Research/NIH 2018/Level Test/Data/Edited
Spreadsheets/05_Dom_Xsec.csv",
	sep=",", row.names=F)
write.table(dom_long,
	file="/Users/oliviafan/Documents/Research/NIH 2018/Level Test/Data/Edited
Spreadsheets/05_Dom_Long.csv",
	sep=",", row.names=F)
write.table(ndom_xsec,
	file="/Users/oliviafan/Documents/Research/NIH 2018/Level Test/Data/Edited
Spreadsheets/05_Nondom_Xsec.csv",
	sep=",", row.names=F)
write.table(ndom_long,
	file="/Users/oliviafan/Documents/Research/NIH 2018/Level Test/Data/Edited
Spreadsheets/05_Nondom_Long.csv",
	sep=",", row.names=F)
```

```r
# ------------------------------------------------------------------------
# Olivia Fan
# Created 07/10/2018
# Edited 07/20/2018
# 06 Average Displacement
# This script calculates the average displacement of a trial by taking the
# distance from the center weighted by the time spent at a point.
# ------------------------------------------------------------------------

# ------------------------------------------------------------------------
# Read data
# ------------------------------------------------------------------------

readData <- function(filename) {
  data <- read.csv(filename, header = TRUE, na.strings = c("", "NA"))
  return(data)
}

dom_xsec <- readData("/Users/oliviafan/Documents/Research/NIH 2018/Level
Test/Data/Edited Spreadsheets/05_Dom_Xsec.csv")
ndom_xsec <- readData("/Users/oliviafan/Documents/Research/NIH 2018/Level
Test/Data/Edited Spreadsheets/05_Nondom_Xsec.csv")
dom_long <- readData("/Users/oliviafan/Documents/Research/NIH 2018/Level
Test/Data/Edited Spreadsheets/05_Dom_Long.csv")
ndom_long <- readData("/Users/oliviafan/Documents/Research/NIH 2018/Level
Test/Data/Edited Spreadsheets/05_Nondom_Long.csv")

# ------------------------------------------------------------------------
# Calculate the average displacement
# ------------------------------------------------------------------------
```

```
TIMEPTS_START = 15

# Helper function to get the Euclidean distance
getEuclidDistance <- function(x1, y1, x2, y2) {
  dist <- sqrt((x1-x2)^2+(y1-y2)^2)
  return(dist)
}

# Function to get the average distance from the center given two coordinates
getDistance <- function(x1, y1, x2, y2) {
  dist1 <- getEuclidDistance(x1, y1, 720, 976)
  dist2 <- getEuclidDistance(x2, y2, 720, 976)
  return((dist1+dist2)/2)
}

# Function to get the time difference given two timepoints
getTime <- function(t1, t2) {
  return(t2 - t1)
}

# Function to get the average displacement
getAvgDisplace <- function(data) {
  avg_displace <- sapply(1:nrow(data), function(i) {
    row <- data[i,]
    row <- row[, colSums(is.na(row)) < nrow(row)] # Get rid of empty columns
    x <- row[c(seq(TIMEPTS_START, length(row), 3))]
    y <- row[c(seq(TIMEPTS_START+1, length(row), 3))]
    t <- row[c(seq(TIMEPTS_START+2, length(row), 3))]
    dist <- getDistance(x[1:(length(x)-1)], y[1:(length(y)-1)], x[2:length(x)], y[2:length(y)])
    time <- getTime(t[1:(length(t)-1)], t[2:length(t)])
    return(sum(dist*time)/sum(time))
```

```
  })
}

# Replace the old average displacement column in the data sheets
dom_xsec$Avg_Displace_.px. <- getAvgDisplace(dom_xsec)
ndom_xsec$Avg_Displace_.px. <- getAvgDisplace(ndom_xsec)
dom_long$Avg_Displace_.px. <- getAvgDisplace(dom_long)
ndom_long$Avg_Displace_.px. <- getAvgDisplace(ndom_long)

# ---------------------------------------------------------------------------
# Export modified data sheets
# ---------------------------------------------------------------------------

write.table(dom_xsec,
        file="/Users/oliviafan/Documents/Research/NIH 2018/Level Test/Data/Edited Spreadsheets/06_Dom_Xsec.csv",
        sep=",", row.names=F)
write.table(dom_long,
        file="/Users/oliviafan/Documents/Research/NIH 2018/Level Test/Data/Edited Spreadsheets/06_Dom_Long.csv",
        sep=",", row.names=F)
write.table(ndom_xsec,
        file="/Users/oliviafan/Documents/Research/NIH 2018/Level Test/Data/Edited Spreadsheets/06_Nondom_Xsec.csv",
        sep=",", row.names=F)
write.table(ndom_long,
        file="/Users/oliviafan/Documents/Research/NIH 2018/Level Test/Data/Edited Spreadsheets/06_Nondom_Long.csv",
        sep=",", row.names=F)
```

```
# ---------------------------------------------------------------------------
# Olivia Fan
# Created 03/21/2018
# Edited 07/20/2018
# 07 Directional Changes
# This script calculates the number of directional changes that in a path.
# Tested: 25, 100, 200 coordinates; 30, 45 degrees; 45, 90 pixels for center
# radius; 45, 90 pixels for distance between consecutive directional changes.
# Concluded: 200 coordinates, 45 degrees, 90 pixels for center radius, and
# 45 pixels for distance between consecutive directional changes.
# ---------------------------------------------------------------------------

# ---------------------------------------------------------------------------
# Read data
# ---------------------------------------------------------------------------

readData <- function(filename) {
  data <- read.csv(filename, header = TRUE, na.strings = c("", "NA"))
  return(data)
}
dom_xsec <- readData("/Users/oliviafan/Documents/Research/NIH 2018/Level
Test/Data/Edited Spreadsheets/06_Dom_Xsec.csv")
ndom_xsec <- readData("/Users/oliviafan/Documents/Research/NIH 2018/Level
Test/Data/Edited Spreadsheets/06_Nondom_Xsec.csv")
dom_long <- readData("/Users/oliviafan/Documents/Research/NIH 2018/Level
Test/Data/Edited Spreadsheets/06_Dom_Long.csv")
ndom_long <- readData("/Users/oliviafan/Documents/Research/NIH 2018/Level
Test/Data/Edited Spreadsheets/06_Nondom_Long.csv")

# ---------------------------------------------------------------------------
# Calculate the number of directional changes
```

```r
# ----------------------------------------------------------------------------

TIMEPTS_START = 15
angle_min_change = 45
center_radius = 90

# Helper function to get the Euclidean distance
getEuclidDistance <- function(x1, y1, x2, y2) {
  dist <- sqrt((x1-x2)^2+(y1-y2)^2)
  return(dist)
}

# Function to get the angle change
getAngleDiff <- function(x1, y1, x2, y2, x3, y3) {
  # Change points to vectors
  a <- c(x2 - x1, y2 - y1)
  b <- c(x3 - x2, y3 - y2)
  # Calculate the angle change
  dot.prod <- a%*%b
  norm.a <- norm(a,type="2")
  norm.b <- norm(b,type="2")
  theta <- acos(dot.prod / (norm.a * norm.b)) / pi * 180
  as.numeric(theta)
  return(theta)
}

# Function to test whether the points are in the center
inCenter <- function(x, y) {
  distance <- sqrt((x-720)^2+(y-976)^2)
  return(distance < center_radius)
}
```

```
# Function to get the number of directional changes
getDirectChanges <- function(data) {
  direct_changes <- sapply(1:nrow(data), function(i) {
    row <- data[i, TIMEPTS_START:ncol(data)]
    row <- row[, colSums(is.na(row)) < nrow(row)] # Get rid of empty columns
    row_x <- as.numeric(row[, seq(1, ncol(row), 3)]) # Extract the x values
    row_y <- as.numeric(row[, seq(2, ncol(row), 3)]) # Extract the y values

    # Remove duplicate coordinates, because those represent staying in one spot
    row_coords <- cbind(row_x, row_y)
    row_coords <- row_coords[!duplicated(row_coords),]
    row_x <- row_coords[, 1]
    row_y <- row_coords[, 2]
    interval = max(1, round(length(row_x)/200, digits = 0))
    row_x_interval <- row_x[seq(1, length(row_x), interval)] # Keep 200 x values
    row_y_interval <- row_y[seq(1, length(row_y), interval)] # Keep 200 y values

    #plot(row_x_interval, row_y_interval, asp = 1)
    #library("plotrix")
    #lines(row_x_interval, row_y_interval)
    #draw.circle(720, 976, radius = 45, border = "gray")
    row_angles_change <- getAngleDiff(row_x_interval[1], row_y_interval[1],
                                      row_x_interval[2], row_y_interval[2],
                                      row_x_interval[3], row_y_interval[3])
    if (length(row_x_interval) > 3) {
      for (x in 2:(length(row_x_interval)-2)) {
        angle_change <- getAngleDiff(row_x_interval[x], row_y_interval[x],
                                     row_x_interval[x+1], row_y_interval[x+1],
                                     row_x_interval[x+2], row_y_interval[x+2])
        row_angles_change <- c(row_angles_change, angle_change)
```

```
  }
}
row_in_center_1 <- inCenter(row_x_interval[1:(length(row_x_interval)-2)],
                            row_y_interval[1:(length(row_y_interval)-2)])
row_in_center_2 <- inCenter(row_x_interval[2:(length(row_x_interval)-1)],
                            row_y_interval[2:(length(row_y_interval)-1)])
row_in_center_3 <- inCenter(row_x_interval[3:length(row_x_interval)],
                            row_y_interval[3:length(row_y_interval)])
row_in_center <- row_in_center_1 & row_in_center_2 & row_in_center_3
direct_change <- !row_in_center & (row_angles_change > angle_min_change)
direct_change <- c(FALSE, direct_change)
num_direct_change <- sum(direct_change)
# If two directional changes are within 45 pixels of each other, only count the first one
x_coord_dc <- row_x_interval[direct_change]
y_coord_dc <- row_y_interval[direct_change]

# Check if consecutive directional changes are within 45 px of each other
if (length(x_coord_dc) > 1) {
  distances <- getEuclidDistance(x_coord_dc[1:(length(x_coord_dc)-1)],
                                 y_coord_dc[1:(length(y_coord_dc)-1)],
                                 x_coord_dc[2:length(x_coord_dc)],
                                 y_coord_dc[2:length(y_coord_dc)])
  count <- 2
  while (count <= length(distances)) {
    if (distances[count-1] < 90) {
      x_coord_dc <- x_coord_dc[-count]
      y_coord_dc <- y_coord_dc[-count]
      distances <- getEuclidDistance(x_coord_dc[1:(length(x_coord_dc)-1)],
                                     y_coord_dc[1:(length(y_coord_dc)-1)],
                                     x_coord_dc[2:length(x_coord_dc)],
                                     y_coord_dc[2:length(y_coord_dc)])
```

```
      }
      else {
        count <- count + 1
      }
    }
  }

  num_direct_change <- length(x_coord_dc)

  #points(x_coord_dc, y_coord_dc, col = "red", pch = 20)

  #print(c(x_coord_dc, y_coord_dc))
  #return(c(x_coord_dc_new, y_coord_dc_new))

  # Use this line to calculate the directional changes
  return(num_direct_change)
 })
}

# Add the number of directional changes to the data sheets right before the timepoints
dom_xsec <- data.frame(dom_xsec[, 1:TIMEPTS_START-1],
                       data.frame(getDirectChanges(dom_xsec)),
                       dom_xsec[, TIMEPTS_START:ncol(dom_xsec)])
ndom_xsec <- data.frame(ndom_xsec[, 1:TIMEPTS_START-1],
                        data.frame(getDirectChanges(ndom_xsec)),
                        ndom_xsec[, TIMEPTS_START:ncol(ndom_xsec)])
dom_long <- data.frame(dom_long[, 1:TIMEPTS_START-1],
                       data.frame(getDirectChanges(dom_long)),
                       dom_long[, TIMEPTS_START:ncol(dom_long)])
ndom_long <- data.frame(ndom_long[, 1:TIMEPTS_START-1],
                        data.frame(getDirectChanges(ndom_long)),
```

```
                ndom_long[, TIMEPTS_START:ncol(ndom_long)])
# Rename headers
header_names <- c("Group", "PID", "Dom_Hand", "Date", "Time", "App_Ver", "Test_Dif",
        "Path_Length_(mm)", "Time_Center_(ms)", "Avg_Displace_(px)",
        "Vel_Overall_(px/sec)", "Vel_Center_(px/sec)", "Amplitude",
        "Frequency", "Directional_Changes")
names(dom_xsec) <- header_names
names(ndom_xsec) <- header_names
names(dom_long) <- header_names
names(ndom_long) <- header_names

# ---------------------------------------------------------------------------
# Export modified data sheets
# ---------------------------------------------------------------------------

write.table(dom_xsec,
      file="/Users/oliviafan/Documents/Research/NIH 2018/Level Test/Data/Edited
Spreadsheets/07_Dom_Xsec.csv",
      sep=",", row.names=F)
write.table(dom_long,
      file="/Users/oliviafan/Documents/Research/NIH 2018/Level Test/Data/Edited
Spreadsheets/07_Dom_Long.csv",
      sep=",", row.names=F)
write.table(ndom_xsec,
      file="/Users/oliviafan/Documents/Research/NIH 2018/Level Test/Data/Edited
Spreadsheets/07_Nondom_Xsec.csv",
      sep=",", row.names=F)
write.table(ndom_long,
      file="/Users/oliviafan/Documents/Research/NIH 2018/Level Test/Data/Edited
Spreadsheets/07_Nondom_Long.csv",
      sep=",", row.names=F)
```

```r
# ---------------------------------------------------------------------------
# Olivia Fan
# Created 05/20/2018
# Edited 07/10/2018
# 08a Select Best Trials
# This script selects the trial with the higher time in center from a sitting
# to be used in the analyses for a given test difficulty.
# ---------------------------------------------------------------------------

# ---------------------------------------------------------------------------
# Read data
# ---------------------------------------------------------------------------

# Function to read the data
readData <- function(filename) {
  data <- read.csv(filename, header = TRUE, na.strings = c(""))
  return(data)
}
dom_xsec <- readData("/Users/oliviafan/Documents/Research/NIH 2018/Level
Test/Data/Edited Spreadsheets/07_Dom_Xsec.csv")
ndom_xsec <- readData("/Users/oliviafan/Documents/Research/NIH 2018/Level
Test/Data/Edited Spreadsheets/07_Nondom_Xsec.csv")
dom_long <- readData("/Users/oliviafan/Documents/Research/NIH 2018/Level
Test/Data/Edited Spreadsheets/07_Dom_Long.csv")
ndom_long <- readData("/Users/oliviafan/Documents/Research/NIH 2018/Level
Test/Data/Edited Spreadsheets/07_Nondom_Long.csv")

# Ignore timepoints
TIMEPTS_START <- 16
dom_xsec <- dom_xsec[, 1:TIMEPTS_START-1]
dom_long <- dom_long[, 1:TIMEPTS_START-1]
```

```r
ndom_xsec <- ndom_xsec[, 1:TIMEPTS_START-1]
ndom_long <- ndom_long[, 1:TIMEPTS_START-1]

# ---------------------------------------------------------------------------
# Select the better trial from each sitting
# ---------------------------------------------------------------------------

library(plyr)

# Function to select the trial with the lower path length
selectBetterTrial <- function(data) {
  # Split the data by patient
  data$PID <- as.factor(as.character(data$PID))
  data <- split(data, data$PID)
  # Split the data by date
  data <- lapply(1:length(data), function(x) {
    data_patient <- data[[x]]
    data_patient$Date <- as.factor(as.character(data_patient$Date))
    data_patient <- split(data_patient, data_patient$Date)
    # Within each date, pick the better trial
    data_patient_new <- lapply(1:length(data_patient), function(y) {
      data_patient_date <- data_patient[[y]]
      data_patient_date_tc <- data_patient_date$Time_Center_.ms.
      data_patient_date_trial <- data_patient_date[which.min(data_patient_date_tc),]
    })
    data_patient <- ldply(data_patient_new, data.frame)
  })
  data <- ldply(data, data.frame)
  return(data)
}
```

```r
# Select the trial with the lower path length
dom_xsec <- selectBetterTrial(dom_xsec)
dom_long <- selectBetterTrial(dom_long)
ndom_xsec <- selectBetterTrial(ndom_xsec)
ndom_long <- selectBetterTrial(ndom_long)

# ---------------------------------------------------------------------------
# Export modified data sheets
# ---------------------------------------------------------------------------

write.table(dom_xsec,
	file="/Users/oliviafan/Documents/Research/NIH 2018/Level Test/Data/Edited Spreadsheets/08a_Dom_Xsec.csv",
	sep=",", row.names=F)
write.table(dom_long,
	file="/Users/oliviafan/Documents/Research/NIH 2018/Level Test/Data/Edited Spreadsheets/08a_Dom_Long.csv",
	sep=",", row.names=F)
write.table(ndom_xsec,
	file="/Users/oliviafan/Documents/Research/NIH 2018/Level Test/Data/Edited Spreadsheets/08a_Nondom_Xsec.csv",
	sep=",", row.names=F)
write.table(ndom_long,
	file="/Users/oliviafan/Documents/Research/NIH 2018/Level Test/Data/Edited Spreadsheets/08a_Nondom_Long.csv",
	sep=",", row.names=F)
```

```r
# ---------------------------------------------------------------------------
# Olivia Fan
# Created 05/20/2018
# Edited 05/20/2018
# 08b Average
# This script selects averages the two trials of a sitting to get one trial.
# This is an alternative to 08a, and does not come after 08a.
# ---------------------------------------------------------------------------

# ---------------------------------------------------------------------------
# Read data
# ---------------------------------------------------------------------------

# Function to read the data
readData <- function(filename) {
  data <- read.csv(filename, header = TRUE, na.strings = c("", "NA"))
  return(data)
}
dom_xsec <- readData("/Users/oliviafan/Documents/Research/NIH 2018/Level
Test/Data/Edited Spreadsheets/07_Dom_Xsec.csv")
ndom_xsec <- readData("/Users/oliviafan/Documents/Research/NIH 2018/Level
Test/Data/Edited Spreadsheets/07_Nondom_Xsec.csv")
dom_long <- readData("/Users/oliviafan/Documents/Research/NIH 2018/Level
Test/Data/Edited Spreadsheets/07_Dom_Long.csv")
ndom_long <- readData("/Users/oliviafan/Documents/Research/NIH 2018/Level
Test/Data/Edited Spreadsheets/07_Nondom_Long.csv")

# Ignore timepoints
TIMEPTS_START <- 16
dom_xsec <- dom_xsec[, 1:TIMEPTS_START-1]
dom_long <- dom_long[, 1:TIMEPTS_START-1]
```

```
ndom_xsec <- ndom_xsec[, 1:TIMEPTS_START-1]
ndom_long <- ndom_long[, 1:TIMEPTS_START-1]

# ----------------------------------------------------------------------------
# Average the two trials of each sitting
# ----------------------------------------------------------------------------

library(plyr)

# Function to average the trials
averageTrials <- function(data) {
  # Split the data by patient
  data$PID <- as.factor(as.character(data$PID))
  data <- split(data, data$PID)
  # Split the data by date
  data <- lapply(1:length(data), function(x) {
    data_patient <- data[[x]]
    data_patient$Date <- as.factor(as.character(data_patient$Date))
    data_patient <- split(data_patient, data_patient$Date)
    # Within each date, average the trials
    data_patient_new <- lapply(1:length(data_patient), function(y) {
      data_patient_date <- data_patient[[y]]
      data_patient_date1 <- data_patient_date[1, 1:7]
      data_patient_date2 <- t(colMeans(data_patient_date[, 8:15], na.rm = T))
      return(cbind(data_patient_date1, data_patient_date2))
    })
    data_patient <- ldply(data_patient_new, data.frame)
  })
  data <- ldply(data, data.frame)
  return(data)
}
```

```r
# Average the trials
dom_xsec <- averageTrials(dom_xsec)
dom_long <- averageTrials(dom_long)
ndom_xsec <- averageTrials(ndom_xsec)
ndom_long <- averageTrials(ndom_long)

# --------------------------------------------------------------------------
# Export modified data sheets
# --------------------------------------------------------------------------

write.table(dom_xsec,
        file="/Users/oliviafan/Documents/Research/NIH 2018/Level Test/Data/Edited Spreadsheets/08b_Dom_Xsec.csv",
        sep=",", row.names=F)
write.table(dom_long,
        file="/Users/oliviafan/Documents/Research/NIH 2018/Level Test/Data/Edited Spreadsheets/08b_Dom_Long.csv",
        sep=",", row.names=F)
write.table(ndom_xsec,
        file="/Users/oliviafan/Documents/Research/NIH 2018/Level Test/Data/Edited Spreadsheets/08b_Nondom_Xsec.csv",
        sep=",", row.names=F)
write.table(ndom_long,
        file="/Users/oliviafan/Documents/Research/NIH 2018/Level Test/Data/Edited Spreadsheets/08b_Nondom_Long.csv",
        sep=",", row.names=F)
```

```
# ---------------------------------------------------------------------------
# Olivia Fan
# Created 05/20/2018
# Edited 07/20/2018
# 09 Days
# This script splits the date/time column and adds a Days column. It also
# removes the average displacement column because we don't care about it, as
# the app update made it unreliable.
# ---------------------------------------------------------------------------

# ---------------------------------------------------------------------------
# Read data
# ---------------------------------------------------------------------------

library(plyr)

# Function to read the data
readData <- function(filename) {
  data <- read.csv(filename, header = TRUE, na.strings = c("", "NA"))
  return(data)
}

# Read the data
dom_xsec <- readData("/Users/oliviafan/Documents/Research/NIH 2018/Level
Test/Data/Edited Spreadsheets/08b_Dom_Xsec.csv")
dom_long <- readData("/Users/oliviafan/Documents/Research/NIH 2018/Level
Test/Data/Edited Spreadsheets/08b_Dom_Long.csv")
ndom_xsec <- readData("/Users/oliviafan/Documents/Research/NIH 2018/Level
Test/Data/Edited Spreadsheets/08b_Nondom_Xsec.csv")
ndom_long <- readData("/Users/oliviafan/Documents/Research/NIH 2018/Level
Test/Data/Edited Spreadsheets/08b_Nondom_Long.csv")
```

```r
# ---------------------------------------------------------------------------
# Add a Days column
# ---------------------------------------------------------------------------

# Function to add a Days column
addDays <- function(data) {
  data1 <- data[, 1:4]
  data2 <- data[, 5:ncol(data)]
  data$PID <- as.factor(as.character(data$PID))
  data <- split(data, data$PID)
  data_days <- lapply(1:length(data), function(x) {
    data_patient <- data[[x]]
    data_patient$Date <- as.Date(as.character(data_patient$Date))
    data_patient_start_date <- min(data_patient$Date)
    data_patient_days <- data_patient$Date - data_patient_start_date
    return(data_patient_days)
  })
  data_days <- ldply(data_days, data.frame)
  data <- cbind(data1, data_days, data2)
  names(data)[5] = "Days"
  return(data)
}

# Add the Days to the datasheets
dom_xsec <- addDays(dom_xsec)
ndom_xsec <- addDays(ndom_xsec)
dom_long <- addDays(dom_long)
ndom_long <- addDays(ndom_long)

# ---------------------------------------------------------------------------
```

```
# Export modified datasheets
# --------------------------------------------------------------------------

write.table(dom_xsec,
	file="/Users/oliviafan/Documents/Research/NIH 2018/Level Test/Data/Edited Spreadsheets/09_Dom_Xsec.csv",
	sep=",", row.names=F)
write.table(dom_long,
	file="/Users/oliviafan/Documents/Research/NIH 2018/Level Test/Data/Edited Spreadsheets/09_Dom_Long.csv",
	sep=",", row.names=F)
write.table(ndom_xsec,
	file="/Users/oliviafan/Documents/Research/NIH 2018/Level Test/Data/Edited Spreadsheets/09_Nondom_Xsec.csv",
	sep=",", row.names=F)
write.table(ndom_long,
	file="/Users/oliviafan/Documents/Research/NIH 2018/Level Test/Data/Edited Spreadsheets/09_Nondom_Long.csv",
	sep=",", row.names=F)
```

```
# ---------------------------------------------------------------------------
# Olivia Fan
# Created 07/16/2018
# Edited 07/16/2018
# Figures Explain
# This script creates Figure 2.
# ---------------------------------------------------------------------------

# ---------------------------------------------------------------------------
# Create an example trial using data
# ---------------------------------------------------------------------------

x <- c(842, 853, 865, 878, 889, 900, 902, 895, 881, 871, 865, 852,
       841, 832, 826, 821, 816, 807, 793, 788, 780, 776, 769, 764, # Timepoint 1
       763, 757, 754, 751, 748, 745, 743, 740, 736, 731, 728, 725,
       723, 721, 719, 716, 714, 712, 710, 708, 706, 704, 702, 700, # Timepoint 2
       694, 688, 682, 677, 672, 667, 662, 657, 653, 649, 645, 640,
       643, 647, 651, 655, 659, 663, 667, 670, 673, 676, 678, 680, # Timepoint 3
       681, 682, 683, 684, 685, 686, 687, 688, 689, 690, 691, 692,
       693, 696, 700, 704, 708, 711, 713, 715, 716, 717, 718, 720, 721) # Timepoint 4
y <- c(1133, 1125, 1122, 1114, 1106, 1099, 1095, 1087, 1083, 1076, 1072, 1064,
       1057, 1050, 1042, 1038, 1031, 1023, 1012, 1008, 1000, 996, 989, 985, # Timepoint 1
       983, 979, 976, 973, 970, 967, 965, 963, 961, 959, 957, 955,
       953, 952, 950, 948, 946, 944, 942, 940, 939, 938, 937, 936, # Timepoint 2
       936, 935, 934, 933, 932, 931, 931, 930, 930, 929, 929, 929,
       930, 931, 933, 935, 937, 939, 941, 943, 945, 947, 949, 951, # Timepoint 3
       953, 955, 956, 958, 959, 960, 961, 962, 963, 964, 965, 966,
       967, 968, 969, 970, 971, 972, 973, 974, 975, 975, 976, 976, 977) # Timepoint 4
# Little invisible points to be used in calculations of the measures
coords_all <- data.frame(x, y)
```

```
# Main points to be plotted
coords_simple <- coords_all[c(1, 25, 49, 73, 97),]

# To be used when drawing the gray circles in the background of the plot
radius_vector <- seq.int(90, 1080, 45)

# --------------------------------------------------------------------------
# Get the measures (path length, time in center, distance from center,
# speed approaching center, direction changes)
# --------------------------------------------------------------------------

# Helper function to get the Euclidean distance
getEuclidDistance <- function(x1, y1, x2, y2) {
  dist <- sqrt((x1-x2)^2+(y1-y2)^2)
  return(dist)
}

# Path length
pl <- c(0, getEuclidDistance(coords_all[1:(nrow(coords_all)-1), 1],
coords_all[1:(nrow(coords_all)-1), 2],
                             coords_all[2:nrow(coords_all), 1], coords_all[2:nrow(coords_all), 2]))
all_pl <- sapply(1:length(pl), function(i) {
  return(sum(pl[1:i]))
})

# Time in center
tc <- sapply(1:(nrow(coords_all)-1), function(i) {
  x1 <- coords_all[i, 1]
  y1 <- coords_all[i, 2]
  x2 <- coords_all[i+1, 1]
  y2 <- coords_all[i+1, 2]
```

```
  if (getEuclidDistance(x1, y1, 720, 976) < 45 & getEuclidDistance(x2, y2, 720, 976) < 45) {
    return(0.0416666666666666667)
  }
  else {
    return(0)
  }
})
tc <- c(0, tc)
all_tc <- sapply(1:length(tc), function(i) {
  return(sum(tc[1:i]))
})

# Distance from center
dfc <- getEuclidDistance(720, 976, coords_all[1:nrow(coords_all), 1],
coords_all[1:nrow(coords_all), 2])

# Speed in center
sc <- sapply(1:(nrow(coords_all)-1), function(i) {
  x1 <- coords_all[i, 1]
  y1 <- coords_all[i, 2]
  x2 <- coords_all[i+1, 1]
  y2 <- coords_all[i+1, 2]
  if (getEuclidDistance(x1, y1, 720, 976) < 45 & getEuclidDistance(x2, y2, 720, 976) < 45) {
    return(getEuclidDistance(x1, y1, x2, y2)/0.0416666666666666667)
  }
  else {
    return(NA)
  }
})
sc <- c(NA, sc)
```

```
# ---------------------------------------------------------------------------
# Plot the path and the measures
# ---------------------------------------------------------------------------

library(plotrix)

png("/Users/oliviafan/Documents/Research/NIH 2018/Level Test/Paper/Explanation.png",
    units="in", width=10, height=5.5, res=600)
layout(matrix(c(1, 1, 1, 1, 2, 3, 4, 5), 2, 4))

# Trial
par(mar = c(5, 5, 4, 1))
plot(c(), asp = 1, xlim = c(510, 930), ylim = c(900, 1052), xlab = "", ylab = "")
draw.circle(720, 976, radius = radius_vector, border = "lightgray")
draw.circle(720, 976, radius = 45, border = "green", col = "green")
lines(coords_all, lwd = 2)
points(coords_simple, pch = 20, cex = 1.5)
segments(902, 1095, 932, 1076, col = "dodgerblue", lwd = 2) # Label angle change 1
draw.arc(902, 1095, radius = 10, col = "dodgerblue", deg1 = 230, deg2 = 325, lwd = 2)
text(901, 1067, expression(paste(theta, " = 99", degree)), srt = 4, col = "dodgerblue", cex =
1.25)
segments(640, 929, 608, 925, col = "dodgerblue", lwd = 2) # Label angle change 2
draw.arc(640, 929, radius = 10, col = "dodgerblue", deg1 = 30, deg2 = 185, lwd = 2)
text(640, 950, expression(paste(theta, " = 140", degree)), srt = 15, col = "dodgerblue", cex =
1.25)
points(c(902, 640), c(1095, 929), pch = 20, col = "red", cex = 1.5) # Direction changes in red
legend("bottomright", pch = 20, col = c("black", "red"), pt.cex = 1.5, cex = 1.25,
       legend = c("Timepoint", "Direction Change"))
coords_lab <- data.frame(c(850, 774, 704, 676, 716),
                         c(1146, 972, 923, 967, 991))
text(coords_lab, labels = c(0:4), cex = 1.5)
```

```
title(ylab = "y coordinates", xlab = "x coordinates", line = 2.5, cex.lab = 1.25)
# Path Length
par(mar = c(3.5, 4, 5.5, 1))
plot(seq(0, 4, length.out = 97), all_pl, xlab = "", ylab = "",
    ylim = c(0, max(all_pl, na.rm = T)), type = "l", lwd = 2)
lines(seq(0.958, 1.958, length.out = 25), all_pl[24:48], col = "green", lwd = 2)
lines(seq(3.042, 4, length.out = 24), all_pl[74:97], col = "green", lwd = 2)
points(c(0:4), all_pl[c(1, 25, 49, 73, 97)], pch = 20, cex = 1.25)
points(c(1, 4), all_pl[c(25, 97)], col = "green", pch = 20, cex = 1.25)
title(ylab = "Path Length (px)", xlab = "Time (s)", line = 2.5, cex.lab = 1.25)
mtext("Path Length", side = 3, line = 0.5)
segments(1.2, 0, 1.2, 253, col = "dodgerblue", lwd = 2)
segments(1.1, 253, 1.2, 253, col = "dodgerblue", lwd = 2)
segments(1.1, 0, 1.2, 0, col = "dodgerblue", lwd = 2)
text(1.3, 126, "Path length from\nt = 0s to t = 1s", adj = 0, col = "dodgerblue", cex = 1.25)
# Distance from center
par(mar = c(5, 4, 4, 1))
plot(seq(0, 4, length.out = 97), dfc, xlab = "", ylab = "",
    ylim = c(0, 1.3*max(dfc, na.rm = T)), type = "l", lwd = 2)
polygon(c(seq(0, 4, length.out = 97), 0),  c(dfc-1, 0), col="gray87", border = NA)
lines(seq(0.958, 1.958, length.out = 25), dfc[24:48], col = "green", lwd = 2)
lines(seq(3.042, 4, length.out = 24), dfc[74:97], col = "green", lwd = 2)
points(c(0:4), dfc[c(1, 25, 49, 73, 97)], pch = 20, cex = 1.25)
points(c(1, 4), dfc[c(25, 97)], col = "green", pch = 20, cex = 1.25)
title(ylab = "Distance from Center (px)", xlab = "Time (s)", line = 2.5, cex.lab = 1.25)
text(0, 1.18*max(dfc, na.rm = T), "Average Distance from\nCenter = AUC/Total Time", col =
"dodgerblue", adj = 0, cex = 1.25)
mtext("Distance from Center", side = 3, line = 0.5)
# Time in Center
par(mar = c(3.5, 4, 5.5, 1))
plot(seq(0, 4, length.out = 97), all_tc, xlab = "", ylab = "",
```

```
     ylim = c(0, max(all_tc, na.rm = T)), type = "l", lwd = 2)
lines(seq(0.958, 1.958, length.out = 25), all_tc[24:48], col = "green", lwd = 2)
lines(seq(3.042, 4, length.out = 24), all_tc[74:97], col = "green", lwd = 2)
points(c(0:4), all_tc[c(1, 25, 49, 73, 97)], pch = 20, cex = 1.25)
points(c(1, 4), all_tc[c(25, 97)], col = "green", pch = 20, cex = 1.25)
text(c(0.5, 2.5), c(0.25, 1.25), labels = c("Not in\ncenter", "Not in\ncenter"), col =
"dodgerblue", cex = 1.25)
title(ylab = "Time in Center (s)", xlab = "Time (s)", line = 2.5, cex.lab = 1.25)
mtext("Time in Center", side = 3, line = 0.5)
# Speed in center
par(mar = c(5, 4, 4, 1))
plot(seq(0, 4, length.out = 97), sc, xlab = "", ylab = "", type = "l", col = "green",
     ylim = c(0, 1.3*max(sc, na.rm = T)), lwd = 2)
polygon(c(1, seq(1, 1.9583333333333333, length.out = 24), 1.9583333333333333),  c(0,
sc[25:48], 0), col="gray87", border = NA)
polygon(c(3.0833333333333333, seq(3.0833333333333333, 4, length.out = 23), 4),  c(0,
sc[75:97], 0), col="gray87", border = NA)
points(c(0:4), sc[c(1, 25, 49, 73, 97)], pch = 20, cex = 1.25)
points(c(1, 4), sc[c(25, 97)], col = "green", pch = 20, cex = 1.25)
title(ylab = "Speed in Center (px/s)", xlab = "Time (s)", line = 2.5, cex.lab = 1.25)
mtext("Speed in Center", side = 3, line = 0.5)
text(0, 1.18*max(sc, na.rm = T), "Average Speed in Center\n= AUC/Time in Center", adj = 0,
col = "dodgerblue", cex = 1.25)
# Label with A, B, C, D, E
mtext("    A                                                                                   B                                            C",
      side = 3, line = -3, cex = 1.25, outer = T, adj = 0, font = 2)
mtext("                                                                                         D                                            E",
      side = 3, line = -22.3, cex = 1.25, outer = T, adj = 0, font = 2)
dev.off()
```

```r
# ---------------------------------------------------------------------------
# Olivia Fan
# Created 07/10/2018
# Edited 07/10/2018
# Correlations
# This script creates figure 3 and calculates correlations of Level Test
# measures with clinical scales.
# ---------------------------------------------------------------------------

# ---------------------------------------------------------------------------
# Read data
# ---------------------------------------------------------------------------

# Level Test
dom_xsec <- read.csv("/Users/oliviafan/Documents/Research/NIH 2018/Level
Test/Data/Edited Spreadsheets/09_Dom_Xsec.csv", header=T)
ndom_xsec <- read.csv("/Users/oliviafan/Documents/Research/NIH 2018/Level
Test/Data/Edited Spreadsheets/09_Nondom_Xsec.csv",header=T)

# Neuro/Aux
neuro_aux <- read.csv("/Users/oliviafan/Documents/Research/NIH 2018/Level
Test/Data/Original Spreadsheets/Neuro_Aux.csv", header=T)
# MSFC
MSFC <- read.csv("/Users/oliviafan/Documents/Research/NIH 2018/Level Test/Data/Original
Spreadsheets/MSFC.csv", header=T)
# NeurEx
NeurEx <- read.csv("/Users/oliviafan/Documents/Research/NIH 2018/Level
Test/Data/Original Spreadsheets/NeurEx.csv", header=T)

# ---------------------------------------------------------------------------
# Function to create the correlation matrices
```

```r
# ---------------------------------------------------------------------------

# Modified from the function corrplot()
corplot2 <- function(corr,
                     method = c("circle", "square", "ellipse", "number", "shade", "color", "pie"),
                     type = c("full", "lower", "upper"), add = FALSE,
                     col = NULL, bg = "white", title = "", is.corr = TRUE,
                     diag = TRUE, outline = TRUE, mar = c(0, 0, 0, 0),
                     addgrid.col = NULL, addCoef.col = NULL, addCoefasPercent = FALSE,

                     order = c("original", "AOE", "FPC", "hclust", "alphabet"),
                     hclust.method = c("complete", "ward", "ward.D", "ward.D2", "single",
                                       "average", "mcquitty", "median", "centroid"),
                     addrect = NULL, rect.col = "black", rect.lwd = 2,

                     tl.pos = NULL, tl.cex = 1,
                     tl.col = "red", tl.offset = 0.5, tl.srt = 90,

                     cl.pos = NULL, cl.lim = NULL,
                     cl.length = NULL, cl.cex = 0.8, cl.ratio = 0.15,
                     cl.align.text = "c", cl.offset = 0.5,

                     number.cex = 1, number.font = 2, number.digits = NULL,

                     addshade = c("negative", "positive", "all"),
                     shade.lwd = 1, shade.col = "white",

                     p.mat = NULL, sig.level = 0.05,
                     insig = c("pch", "p-value", "blank", "n", "label_sig"),
                     pch = 4, pch.col = "black", pch.cex = 3,
```

```
                     plotCI = c("n", "square", "circle", "rect"),
                     lowCI.mat = NULL, uppCI.mat = NULL,
                     na.label = "?", na.label.col = "black",
                     win.asp = 1,
                     ...)
{

  # checking multi-option input parameters
  method <- match.arg(method)
  type <- match.arg(type)
  order <- match.arg(order)
  hclust.method <- match.arg(hclust.method)
  addshade <- match.arg(addshade)
  insig <- match.arg(insig)
  plotCI <- match.arg(plotCI)

  # rescale symbols within the corrplot based on win.asp parameter
  if (win.asp != 1 && !(method %in% c("circle", "square"))) {
    stop("Parameter 'win.asp' is supported only for circle and square methods.")
  }
  asp_rescale_factor <- min(1, win.asp) / max(1, win.asp)
  stopifnot(asp_rescale_factor >= 0 && asp_rescale_factor <= 1)

  if (!is.matrix(corr) && !is.data.frame(corr)) {
    stop("Need a matrix or data frame!")
  }

  # select grid color automatically if not specified
  if (is.null(addgrid.col)) {
    addgrid.col <- switch(method, color = NA, shade = NA, "grey")
  }
```

```
if (any(corr < cl.lim[1]) || any(corr > cl.lim[2])) {
  stop("color limits should cover matrix")
}

if (is.null(cl.lim)) {
  if (is.corr) {
    # if the matrix is expected to be a correlation matrix
    # it MUST be within the interval [-1,1]
    cl.lim <- c(-1,1)
  } else {
    # Issue #91
    # if not a correlation matrix and the diagonal is hidden,
    # we need to compute limits from all cells except the diagonal
    corr_tmp <- corr
    diag(corr_tmp) <- ifelse(diag, diag(corr_tmp), NA)
    cl.lim <- c(min(corr_tmp, na.rm = TRUE), max(corr_tmp, na.rm = TRUE))
  }
}

intercept <- 0
zoom <- 1

if (!is.corr) {

  c_max <- max(corr, na.rm = TRUE)
  c_min <- min(corr, na.rm = TRUE)

  # The following if-elseif-else code should exhaustively cover all 9
  # combinations of c_min and c_max variables. Each variable can be either
  # zero (0), positive (+) or negative (-).
```

```r
# c_min c_max

# 00
# -0
# +0
# --
# 0-
if (c_max <= 0) {
  intercept <- -cl.lim[2]
  zoom <- 1 / (diff(cl.lim))
}

# ++
# +-
# 0+
else if (c_min >= 0) {
  intercept <- -cl.lim[1]
  zoom <- 1 / (diff(cl.lim))
}

# -+
else {

  # expression from the original code as a sanity check
  stopifnot(c_max * c_min < 0)

  # newly derived expression which covers the single remainig case
  stopifnot(c_min < 0 && c_max > 0)

  intercept <- 0
```

```
    zoom <- 1 / max(abs(cl.lim))
  }

  # now, the zoom might still be Inf when cl.lim were both zero
  if (zoom == Inf) {
    stopifnot(cl.lim[1] == 0 && cl.lim[2] == 0) # check the assumption
    zoom <- 0
  }

  corr <- (intercept + corr) * zoom
}

cl.lim2 <- (intercept + cl.lim) * zoom
int <- intercept * zoom

if (is.corr) {
  # check the interval if expecting a correlation matrix
  # otherwise, the values can be any number
  if (min(corr, na.rm = TRUE) < -1 - .Machine$double.eps ^ .75 ||
      max(corr, na.rm = TRUE) >  1 + .Machine$double.eps ^ .75 ) {
    stop("The matrix is not in [-1, 1]!")
  }
}

if (is.null(col)) {
  col <- colorRampPalette(c("#67001F", "#B2182B", "#D6604D", "#F4A582",
                            "#FDDBC7", "#FFFFFF", "#D1E5F0", "#92C5DE",
                            "#4393C3", "#2166AC", "#053061"))(200)
}

n <- nrow(corr)
```

```
m <- ncol(corr)
min.nm <- min(n,m)
ord <- seq_len(min.nm)

if (order != "original") {
  ord <- corrMatOrder(corr, order = order, hclust.method = hclust.method)
  corr <- corr[ord, ord]
}

## set up variable names
if (is.null(rownames(corr))) {
  rownames(corr) <- seq_len(n)
}
if (is.null(colnames(corr))) {
  colnames(corr) <- seq_len(m)
}

# assigns Inf to cells in the matrix depending on the type paramter
apply_mat_filter <- function(mat) {
  x <- matrix(1:n * m, nrow = n, ncol = m)
  switch(type,
         upper = mat[row(x) > col(x)] <- Inf,
         lower = mat[row(x) < col(x)] <- Inf
  )

  if (!diag) {
    diag(mat) <- Inf
  }
  return(mat)
}
```

```r
# retrieves coordinates of cells to be rendered
getPos.Dat <- function(mat) {
  tmp <- apply_mat_filter(mat)
  Dat <- tmp[is.finite(tmp)]
  ind  <- which(is.finite(tmp), arr.ind = TRUE)
  Pos <- ind
  Pos[,1] <-  ind[,2]
  Pos[,2] <- -ind[,1] + 1 + n
  return(list(Pos, Dat))
}

# retrieves coordinates of NA cells
# we use this for rending NA cells differently
getPos.NAs <- function(mat) {
  tmp <- apply_mat_filter(mat)
  ind <- which(is.na(tmp), arr.ind = TRUE)
  Pos <- ind
  Pos[,1] <-  ind[,2]
  Pos[,2] <- -ind[,1] + 1 + n
  return(Pos)
}

Pos <- getPos.Dat(corr)[[1]]

# decide whether NA labels are going to be rendered or whether we ignore them
if (any(is.na(corr)) && is.character(na.label)) {
  PosNA <- getPos.NAs(corr)
} else {
  # explicitly set to NULL to indicate that NA labels are not going to be
  # rendered
  PosNA <- NULL
```

```
}

AllCoords <- rbind(Pos, PosNA)

# rows
n2 <- max(AllCoords[,2])
n1 <- min(AllCoords[,2])

nn <- n2 - n1

# columns
m2 <- max(AllCoords[,1])
m1 <- min(AllCoords[,1])

# Issue #19: legend color bar width 0 when using just one column matrix
# also discussed here: http://stackoverflow.com/questions/34638555/
mm <- max(1, m2 - m1)

# Issue #20: support plotmath expressions in rownames and colnames
expand_expression <- function(s) {
  ifelse(grepl("^[:=$]", s), parse(text = substring(s, 2)), s)
}

newrownames <- sapply(
  rownames(corr)[(n + 1 - n2):(n + 1 - n1)], expand_expression)

newcolnames <- sapply(
  colnames(corr)[m1:m2], expand_expression)

DAT <- getPos.Dat(corr)[[2]]
len.DAT <- length(DAT)
```

```
rm(expand_expression) # making sure the function is only used here

## assign colors
assign.color <- function(dat = DAT, color = col) {
  newcorr <- (dat + 1) / 2
  newcorr[newcorr <= 0]  <- 0
  newcorr[newcorr >= 1]  <- 1 - 1e-16

  color[floor(newcorr * length(color)) + 1] # new color returned
}

col.fill <- assign.color()

isFALSE <- function(x) identical(x, FALSE)
isTRUE <- function(x) identical(x, TRUE)

if (isFALSE(tl.pos)) {
  tl.pos <- "n"
}

if (is.null(tl.pos) || isTRUE(tl.pos)) {
  tl.pos <- switch(type, full = "lt", lower = "ld", upper = "td")
}

if (isFALSE(cl.pos)) {
  cl.pos <- "n"
}

if (is.null(cl.pos) || isTRUE(cl.pos)) {
  cl.pos <- switch(type, full = "r", lower = "b", upper = "r")
```

```
}

if (isFALSE(outline)) {
  col.border <- col.fill
} else if (isTRUE(outline)) {
  col.border <- "black"
} else if (is.character(outline)) {
  col.border <- outline
} else {
  stop("Unsupported value type for parameter outline")
}

# restore this parameter when exiting the corrplot function in any way
oldpar <- par(mar = mar, bg = "white")
on.exit(par(oldpar), add = TRUE)

## calculate label-text width approximately
if (!add) {
  plot.new()

  # Issue #10: code from Sebastien Rochette (github user @statnmap)
  xlabwidth <- max(strwidth(newrownames, cex = tl.cex))
  ylabwidth <- max(strwidth(newcolnames, cex = tl.cex))
  laboffset <- strwidth("W", cex = tl.cex) * tl.offset

  # Issue #10
  for (i in 1:50) {
    xlim <- c(
      m1 - 0.5 - laboffset -
        xlabwidth * (grepl("l", tl.pos) | grepl("d", tl.pos)),
      m2 + 0.5 + mm * cl.ratio * (cl.pos == "r") +
```

```
    xlabwidth * abs(cos(tl.srt * pi / 180)) * grepl("d", tl.pos)
) + c(-0.35, 0.15) +
  c(-1,0) * grepl("l", tl.pos) # margin between text and grid

ylim <- c(
  n1 - 0.5 - nn * cl.ratio * (cl.pos == "b") - laboffset,
  n2 + 0.5 + laboffset +
    ylabwidth * abs(sin(tl.srt * pi / 180)) * grepl("t", tl.pos)
) +
  c(-0.15, 0) +
  c(0, -1) * (type == "upper" && tl.pos != "n") + # nasty hack
  c(0,1) * grepl("d", tl.pos) # margin between text and grid

# note: the nasty hack above is related to multiple issues
# (e.g. #96, #94, #102)

plot.window(xlim, ylim, asp = 1, xaxs = "i", yaxs = "i")

x.tmp <- max(strwidth(newrownames, cex = tl.cex))
y.tmp <- max(strwidth(newcolnames, cex = tl.cex))

laboffset.tmp <- strwidth("W", cex = tl.cex) * tl.offset
if (max(x.tmp - xlabwidth,
        y.tmp - ylabwidth,
        laboffset.tmp - laboffset) < 1e-03) {
  break
}

xlabwidth <- x.tmp
ylabwidth <- y.tmp
```

```
    laboffset <- laboffset.tmp

    if (i == 50) {
      warning(c("Not been able to calculate text margin, ",
                "please try again with a clean new empty window using ",
                "{plot.new(); dev.off()} or reduce tl.cex"))
    }
  }

  if (.Platform$OS.type == "windows") {
    grDevices::windows.options(width = 7,
                               height = 7 * diff(ylim) / diff(xlim))
  }

  plot.window(xlim = xlim , ylim = ylim,
              asp = win.asp, xlab = "", ylab = "", xaxs = "i", yaxs = "i")
}

## for: add = TRUE
laboffset <- strwidth("W", cex = tl.cex) * tl.offset

## background for the cells
symbols(Pos, add = TRUE, inches = FALSE,
        rectangles = matrix(1, len.DAT, 2), bg = bg, fg = bg)

## circle
if (method == "circle" && plotCI == "n") {
  symbols(Pos, add = TRUE,  inches = FALSE,
          circles = asp_rescale_factor * 0.9 * abs(DAT) ^ 0.5 / 2,
          fg = col.border, bg = col.fill)
}
```

```r
## ellipse
if (method == "ellipse" && plotCI == "n") {
  ell.dat <- function(rho, length = 99) {
    k <- seq(0, 2 * pi, length = length)
    x <- cos(k + acos(rho) / 2) / 2
    y <- cos(k - acos(rho) / 2) / 2
    cbind(rbind(x,y), c(NA, NA))
  }

  ELL.dat <- lapply(DAT, ell.dat)
  ELL.dat2 <- 0.85 * matrix(unlist(ELL.dat), ncol = 2, byrow = TRUE)
  ELL.dat2 <- ELL.dat2  + Pos[rep(1:length(DAT),each = 100),]
  polygon(ELL.dat2, border = col.border, col = col.fill)
}

## number
if (is.null(number.digits)) {
  # TODO: this expression might be confusing
  number.digits <- switch(addCoefasPercent + 1, 2, 0)
}

stopifnot(number.digits %% 1 == 0)  # is whole number
stopifnot(number.digits >= 0)       # is non-negative number

if (method == "number" && plotCI == "n") {
  text(Pos[,1], Pos[,2], font = number.font, col = col.fill,
       labels = round((DAT - int) * ifelse(addCoefasPercent, 100, 1) / zoom,
                      number.digits),
       cex = number.cex)
}
```

```r
# Issue #55: Support for multiple characters when rendering NAs
NA_LABEL_MAX_CHARS <- 2

# renders NA cells
if (is.matrix(PosNA) && nrow(PosNA) > 0) {

  stopifnot(is.matrix(PosNA)) # sanity check

  if (na.label == "square") {
    symbols(PosNA, add = TRUE, inches = FALSE,
            squares = rep(1, nrow(PosNA)),
            bg = na.label.col, fg = na.label.col)
  } else if (nchar(na.label) %in% 1:NA_LABEL_MAX_CHARS) {
    symbols(PosNA, add = TRUE, inches = FALSE,
            squares = rep(1, nrow(PosNA)), fg = bg, bg = bg)
    text(PosNA[,1], PosNA[,2], font = number.font,
         col = na.label.col,
         labels = na.label, cex = number.cex, ...)
  } else {
    stop(paste("Maximum number of characters for NA label is:",
               NA_LABEL_MAX_CHARS))
  }
}

## pie
if (method == "pie" && plotCI == "n") {

  # Issue #18: Corrplot background circle
  symbols(Pos, add = TRUE, inches = FALSE,
          circles = rep(0.5, len.DAT) * 0.85, fg = col.border)
```

```
  pie.dat <- function(theta, length = 100) {
    k <- seq(pi / 2, pi / 2 - theta, length = 0.5 * length * abs(theta) / pi)
    x <- c(0, cos(k) / 2, 0)
    y <- c(0, sin(k) / 2, 0)
    cbind(rbind(x,y), c(NA, NA)) # pie.dat returned
  }

  PIE.dat <- lapply(DAT * 2 * pi, pie.dat)
  len.pie <- unlist(lapply(PIE.dat, length)) / 2
  PIE.dat2 <- 0.85 * matrix(unlist(PIE.dat), ncol = 2, byrow = TRUE)
  PIE.dat2 <- PIE.dat2  + Pos[rep(1:length(DAT), len.pie),]
  polygon(PIE.dat2, border = "black", col = col.fill)
}

## shade
if (method == "shade" && plotCI == "n") {
  symbols(Pos, add = TRUE, inches = FALSE, squares = rep(1, len.DAT),
          bg = col.fill, fg = addgrid.col)

  shade.dat <- function(w) {
    x <- w[1]
    y <- w[2]
    rho <- w[3]
    x1 <- x - 0.5
    x2 <- x + 0.5
    y1 <- y - 0.5
    y2 <- y + 0.5
    dat <- NA

    if ((addshade == "positive" || addshade == "all") && rho > 0) {
```

```
    dat <- cbind(c(x1, x1, x), c(y, y1, y1),
                 c(x, x2, x2), c(y2, y2 ,y))
   }

   if ((addshade == "negative" || addshade == "all") && rho < 0) {
    dat <- cbind(c(x1, x1, x), c(y, y2, y2),
                 c(x, x2, x2), c(y1, y1 ,y))
   }

   return(t(dat))
  }

  pos_corr <- rbind(cbind(Pos, DAT))
  pos_corr2 <- split(pos_corr, 1:nrow(pos_corr))

  SHADE.dat <- matrix(na.omit(unlist(lapply(pos_corr2, shade.dat))),
                    byrow = TRUE, ncol = 4)

  segments(SHADE.dat[,1], SHADE.dat[,2], SHADE.dat[,3],
           SHADE.dat[,4], col = shade.col, lwd = shade.lwd)
}

## square
if (method == "square" && plotCI == "n") {
  draw_method_square(Pos, DAT, asp_rescale_factor, col.border, col.fill)
}

## color
if (method == "color" && plotCI == "n") {
  draw_method_color(Pos, col.border, col.fill)
}
```

```r
## add grid
draw_grid(AllCoords, addgrid.col)

if (plotCI != "n") {

  if (is.null(lowCI.mat) || is.null(uppCI.mat)) {
    stop("Need lowCI.mat and uppCI.mat!")
  }

  if (order != "original") {
    lowCI.mat <- lowCI.mat[ord, ord]
    uppCI.mat <- uppCI.mat[ord, ord]
  }

  pos.lowNew  <- getPos.Dat(lowCI.mat)[[1]]
  lowNew      <- getPos.Dat(lowCI.mat)[[2]]
  pos.uppNew  <- getPos.Dat(uppCI.mat)[[1]]
  uppNew      <- getPos.Dat(uppCI.mat)[[2]]

  if (!method %in% c("circle", "square")) {
    stop("Method shoud be circle or square if drawing confidence intervals.")
  }

  k1 <- (abs(uppNew) > abs(lowNew))
  bigabs <- uppNew
  bigabs[which(!k1)] <- lowNew[!k1]
  smallabs <- lowNew
  smallabs[which(!k1)] <- uppNew[!k1]
  sig <- sign(uppNew * lowNew)
```

```
color_bigabs <- col[ceiling((bigabs + 1) * length(col) / 2)]
color_smallabs <- col[ceiling((smallabs + 1) * length(col) / 2)]

if (plotCI == "circle") {

  symbols(pos.uppNew[,1], pos.uppNew[,2],
          add = TRUE,  inches = FALSE,
          circles = 0.95 * abs(bigabs) ^ 0.5 / 2,
          bg = ifelse(sig > 0, col.fill, color_bigabs),
          fg = ifelse(sig > 0, col.fill, color_bigabs)
  )

  symbols(pos.lowNew[,1], pos.lowNew[,2],
          add = TRUE, inches = FALSE,
          circles = 0.95 * abs(smallabs) ^ 0.5 / 2,
          bg = ifelse(sig > 0, bg, color_smallabs),
          fg = ifelse(sig > 0, col.fill, color_smallabs))
}

if (plotCI == "square") {
  symbols(pos.uppNew[,1], pos.uppNew[,2],
          add = TRUE,  inches = FALSE,
          squares = abs(bigabs) ^ 0.5,
          bg = ifelse(sig > 0, col.fill, color_bigabs),
          fg = ifelse(sig > 0, col.fill, color_bigabs))

  symbols(pos.lowNew[,1], pos.lowNew[,2],
          add = TRUE, inches = FALSE,
          squares = abs(smallabs) ^ 0.5,
          bg = ifelse(sig > 0, bg, color_smallabs),
          fg = ifelse(sig > 0, col.fill, color_smallabs))
```

```
  }

  if (plotCI == "rect") {
    rect.width <- 0.25
    rect(pos.uppNew[,1] - rect.width, pos.uppNew[,2] + smallabs / 2,
         pos.uppNew[,1] + rect.width, pos.uppNew[,2] + bigabs / 2,
         col = col.fill, border = col.fill)
    segments(pos.lowNew[,1] - rect.width, pos.lowNew[,2] + DAT / 2,
             pos.lowNew[,1] + rect.width, pos.lowNew[,2] + DAT / 2,
             col = "black", lwd = 1)
    segments(pos.uppNew[,1] - rect.width, pos.uppNew[,2] + uppNew / 2,
             pos.uppNew[,1] + rect.width, pos.uppNew[,2] + uppNew / 2,
             col = "black", lwd = 1)
    segments(pos.lowNew[,1] - rect.width, pos.lowNew[,2] + lowNew / 2,
             pos.lowNew[,1] + rect.width, pos.lowNew[,2] + lowNew / 2,
             col = "black", lwd = 1)
    segments(pos.lowNew[,1] - 0.5,pos.lowNew[,2],
             pos.lowNew[,1] + 0.5, pos.lowNew[,2], col = "grey70", lty = 3)
  }
}

if (!is.null(p.mat) && insig != "n") {
  if (order != "original") {
    p.mat <- p.mat[ord, ord]
  }

  pos.pNew  <- getPos.Dat(p.mat)[[1]]
  pNew      <- getPos.Dat(p.mat)[[2]]

  if (insig == "label_sig") {
```

```r
# Unless another character is specified, mark sig with *
if (!is.character(pch))
  pch <- "*"

place_points <- function(sig.locs, point) {
  text(pos.pNew[,1][sig.locs], (pos.pNew[,2][sig.locs]+0.25),
       labels = point, col = pch.col, cex = pch.cex, lwd = 2)
}

if (length(sig.level) == 1) {
  place_points(sig.locs = which(pNew < sig.level), point = pch)

} else {
  l <- length(sig.level)
  for (i in seq_along(sig.level)) {
    iter <- l + 1 - i
    pchTmp <- paste(rep(pch, i), collapse = "")
    if (i == length(sig.level)) {
      locs <- which(pNew < sig.level[iter])
      if (length(locs)) {
        place_points(sig.locs = locs, point = pchTmp)
      }
    } else {
      locs <- which(pNew < sig.level[iter] & pNew > sig.level[iter - 1])
      if (length(locs)) {
        place_points(sig.locs = locs, point = pchTmp)
      }
    }
  }

}
```

```
  } else {

    ind.p <- which(pNew > sig.level)
    p_inSig <- length(ind.p) > 0

    if (insig == "pch" && p_inSig) {
      points(pos.pNew[,1][ind.p], pos.pNew[,2][ind.p],
             pch = pch, col = pch.col, cex = pch.cex, lwd = 2)
    }

    if (insig == "p-value" && p_inSig) {
      text(pos.pNew[,1][ind.p], pos.pNew[,2][ind.p],
           round(pNew[ind.p],2), col = pch.col)
    }

    if (insig == "blank" && p_inSig) {
      symbols(pos.pNew[,1][ind.p], pos.pNew[,2][ind.p], inches = FALSE,
              squares = rep(1, length(pos.pNew[,1][ind.p])),
              fg = addgrid.col, bg = bg, add = TRUE)
    }
  }
}

### color legend
if (cl.pos != "n") {
  colRange <- assign.color(dat = cl.lim2)
  ind1 <- which(col == colRange[1])
  ind2 <- which(col == colRange[2])
  colbar <- col[ind1:ind2]
```

```
    if (is.null(cl.length)) {
      cl.length <- ifelse(length(colbar) > 20, 11, length(colbar) + 1)
    }

    labels <- seq(cl.lim[1], cl.lim[2], length = cl.length)

    if (cl.pos == "r") {
      vertical <- TRUE
      xlim <- c(m2 + 0.5 + mm * 0.02, m2 + 0.5 + mm * cl.ratio)
      ylim <- c(n1 - 0.5, n2 + 0.5)
    }

    if (cl.pos == "b") {
      vertical <- FALSE
      xlim <- c(m1 - 0.5, m2 + 0.5)
      ylim <- c(n1 - 0.5 - nn * cl.ratio, n1 - 0.5 - nn * 0.02)
    }

    colorlegend(colbar = colbar, labels = round(labels, 2),
                offset = cl.offset, ratio.colbar = 0.3, cex = cl.cex,
                xlim = xlim, ylim = ylim, vertical = vertical,
                align = cl.align.text)
  }

  ## add variable names and title
  if (tl.pos != "n") {
    pos.xlabel <- cbind(m1:m2, n2 + 0.5 + laboffset)
    pos.ylabel <- cbind(m1 - 0.5, n2:n1)

    if (tl.pos == "td") {
      if (type != "upper") {
```

```
    stop("type should be \"upper\" if tl.pos is \"dt\".")
   }
   pos.ylabel <- cbind(m1:(m1 + nn) - 0.5, n2:n1)
  }

  if (tl.pos == "ld") {
   if (type != "lower") {
    stop("type should be \"lower\" if tl.pos is \"ld\".")
   }
   pos.xlabel <- cbind(m1:m2, n2:(n2 - mm) + 0.5 + laboffset)
  }

  if (tl.pos == "d") {
   pos.ylabel <- cbind(m1:(m1 + nn) - 0.5, n2:n1)
   pos.ylabel <- pos.ylabel[1:min(n, m),]

   symbols(pos.ylabel[,1] + 0.5, pos.ylabel[,2], add = TRUE,
           bg = bg, fg = addgrid.col,
           inches = FALSE, squares = rep(1, length(pos.ylabel[,1])))

   text(pos.ylabel[,1] + 0.5, pos.ylabel[,2], newcolnames[1:min(n, m)],
        col = tl.col, cex = tl.cex, ...)

  } else {
   text(pos.xlabel[,1], pos.xlabel[,2], newcolnames, srt = tl.srt,
        adj = ifelse(tl.srt == 0, c(0.5,0), c(0,0)),
        col = tl.col, cex = tl.cex, offset = tl.offset, ...)
   text(pos.ylabel[,1], pos.ylabel[,2], newrownames,
        col = tl.col, cex = tl.cex, pos = 2, offset = tl.offset, ...)
  }
}
```

```
  title(title, ...)

  ## add numbers
  if (!is.null(addCoef.col) && method != "number") {
    text(Pos[,1], Pos[,2],  col = addCoef.col,
         #labels = round((DAT - int) * ifelse(addCoefasPercent, 100, 1) / zoom,
         #               number.digits),
         labels = formatC(round((DAT - int) * ifelse(addCoefasPercent, 100, 1) / zoom,
                               number.digits), digits = 2, format = "f"),
         cex = number.cex, font = number.font)
  }

  ## add grid, in case of the grid is ate when "diag=FALSE"
  if (type == "full" && plotCI == "n" && !is.null(addgrid.col)) {
    rect(m1 - 0.5, n1 - 0.5, m2 + 0.5, n2 + 0.5, border = addgrid.col)
  }

  ##  draws rectangles, call function corrRect.hclust
  if (!is.null(addrect) && order == "hclust" && type == "full") {
    corrRect.hclust(corr, k = addrect, method = hclust.method,
                    col = rect.col, lwd = rect.lwd)
  }

  invisible(corr) # reordered correlation matrix
}

#' @note pure function
#' @noRd
draw_method_square <- function(coords, values, asp_rescale_factor, fg, bg) {
  symbols(coords, add = TRUE, inches = FALSE,
```

```
        squares = asp_rescale_factor * abs(values) ^ 0.5,
        bg = bg, fg = fg)
}

#' @note pure function
#' @noRd
draw_method_color <- function(coords, fg, bg) {
  symbols(coords, squares = rep(1, nrow(coords)), fg = fg, bg = bg,
        add = TRUE, inches = FALSE)
}

#' @note pure function
#' @noRd
draw_grid <- function(coords, fg) {
  symbols(coords, add = TRUE, inches = FALSE, fg = fg, bg = NA,
        rectangles = matrix(1, nrow = nrow(coords), ncol = 2))
}

# ---------------------------------------------------------------------------
# Extract and add neuro/aux data
# ---------------------------------------------------------------------------

library(plyr)

# Get the following data from neuro_aux: EDSS, SNRS, CombiWISE
getScoresNeuroAux <- function(data) {
  # Create data.frame with the scores for each line
  scores_neuro_aux <- lapply(1:nrow(data), function(i) {
    PID <- as.character(data[i, 2])
    index <- max(which(as.character(neuro_aux[, 1]) == PID))
    if (is.finite(index)) {
```

```
      cur_scores <- neuro_aux[index, c(10, 11, 5)]
      colnames(cur_scores) = c("EDSS", "SNRS", "CombiWISE")
    }
    else {
      cur_scores <- data.frame(NA, NA, NA)
      colnames(cur_scores) = c("EDSS", "SNRS", "CombiWISE")
    }
    return(cur_scores)
  })
}

# Get the following data from MSFC: SDMT correct, MSFC, 9HPT (only non-dom), 25FW,
PASAT
getScoresMSFC <- function(data, dominant) {
  colnames_all <- c("SDMT", "MSFC", "9HPT", "25FW", "PASAT")
  # Create data.frame with the scores for each line
  scores_MSFC <- lapply(1:nrow(data), function(i) {
    PID <- as.character(data[i, 2])
    dom_hand <- as.character(data[i, 3])
    index <- max(which(as.character(MSFC[, 1]) == PID))
    names(MSFC)
    if (is.finite(index)) {
      # Right hand and dominant (right hand data)
      if (dom_hand == "Right" & dominant) {
        cur_scores <- MSFC[index, c(23, 7, 16, 9, 19)]
      }
      # Right hand and non-dominant (left hand data)
      else if (dom_hand == "Right" & !dominant) {
        cur_scores <- MSFC[index, c(23, 7, 13, 9, 19)]
      }
      # Left hand and dominant (left hand data)
```

```
      else if (dom_hand == "Left" & dominant) {
        cur_scores <- MSFC[index, c(23, 7, 13, 9, 19)]
      }
      # Left hand and non-dominant (right hand data)
      else if (dom_hand == "Left" & !dominant) {
        cur_scores <- MSFC[index, c(23, 7, 16, 9, 19)]
      }
      colnames(cur_scores) <- colnames_all
    }
    else {
      cur_scores <- data.frame(NA, NA, NA, NA, NA)
      colnames(cur_scores) <- colnames_all
    }
    return(cur_scores)
  })
}

# Get the following data from neuro_aux: EDSS, SNRS, CombiWISE
getScoresNeurEx <- function(data, dominant) {
  # Create data.frame with the scores for each line
  scores_neuro_aux <- lapply(1:nrow(data), function(i) {
    PID <- as.character(data[i, 2])
    dom_hand <- as.character(data[i, 3])
    index <- max(which(as.character(NeurEx[, 1]) == PID))
    colnames_all<- c("Cognitive Functions", "Eyes", "Eye Movements", "Visual Fields",
"Brainstem/Remaining\nCranial Nerves",
                     "Brainstem/Lower\nCranial Nerves", "Pyramidal Signs &\nMotor Fatigue",
"Upper Extremities\nStrength",
                     "Lower Extremities\nStrength", "Reflexes", "Muscle Atrophy", "Cerebellar
Functions", "Dermatome & S-G",
                     "Pains & Paresthesias", "Positive Phenomena", "Stance & Gait", "Bowel,
```

```
Bladder, &\nSexual Functions",
                    "Vibrations in Fingers", "Vibrations in Wrist")
    if (is.finite(index)) {
      # Extremities
      names(NeurEx)
      # Right hand and dominant (right hand data)
      if (dom_hand == "Right" & dominant) {
        cur_scores <- NeurEx[index, c(46, 47, 48, 49, 50, 51, 39, 40, 22, 23, 34, 35, 36, 38, 60,
61, 62, 67, 73)]
      }
      # Right hand and non-dominant (left hand data)
      else if (dom_hand == "Right" & !dominant) {
        cur_scores <- NeurEx[index, c(46, 47, 48, 49, 50, 51, 30, 31, 13, 14, 25, 26, 27, 29, 60,
61, 62, 66, 72)]
      }
      # Left hand and dominant (left hand data)
      else if (dom_hand == "Left" & dominant) {
        cur_scores <- NeurEx[index, c(46, 47, 48, 49, 50, 51, 30, 31, 13, 14, 25, 26, 27, 29, 60,
61, 62, 66, 72)]
      }
      # Left hand and non-dominant (right hand data)
      else if (dom_hand == "Left" & !dominant) {
        cur_scores <- NeurEx[index, c(46, 47, 48, 49, 50, 51, 39, 40, 22, 23, 34, 35, 36, 38, 60,
61, 62, 67, 73)]
      }
      colnames(cur_scores) <- colnames_all
    }
    else {
      cur_scores <- data.frame(NA, NA, NA, NA, NA, NA, NA, NA, NA, NA, NA, NA, NA,
NA, NA, NA, NA, NA, NA)
      colnames(cur_scores) <- colnames_all
```

```r
    }
    return(cur_scores)
  })
}

# Add the neuroscores to the datasheets
dom_xsec <- cbind(dom_xsec,
                  ldply(getScoresNeuroAux(dom_xsec), rbind),
                  ldply(getScoresMSFC(dom_xsec, TRUE), rbind),
                  ldply(getScoresNeurEx(dom_xsec, TRUE), rbind))
ndom_xsec <- cbind(ndom_xsec,
                   ldply(getScoresNeuroAux(ndom_xsec), rbind),
                   ldply(getScoresMSFC(ndom_xsec, FALSE), rbind),
                   ldply(getScoresNeurEx(ndom_xsec, FALSE), rbind))

# ---------------------------------------------------------------------------
# Create correlation matrices
# ---------------------------------------------------------------------------

library(corrplot)
library(Hmisc)

# Custom color palette
library(RColorBrewer)
custom_col <- colorRampPalette(c(rgb(0, 0, 255, max = 255),
                                 rgb(51, 153, 255, max = 255),
                                 "white",
                                 rgb(255, 102, 102, max = 255),
                                 rgb(255, 0, 0, max = 255)))(100)

# Function to create the correlation matrix with general clinical scales
```

```
createCorMatrixScales <- function(data, title_text) {
  # data <- dom_xsec
  # Get the r values
  cor_data <<- cor(data[, c(9, 10, 11, 13, 16)], data[, 17:24], method = "spearman",
                   use = "complete.obs")
  rownames(cor_data) <- c("Path Length", "Time in Center", "Average Distance\nfrom
Center",
                          "Average Speed in\nCenter", "Direction Changes")
  cor_data <- signif(cor_data, 2)
  # Get the p values
  sig_data <- rcorr(as.matrix(cbind(data[, c(9, 10, 11, 13, 16)], data[, 17:24])),
                    type = "spearman")$P
  sig_data <- sig_data[1:5, 6:13]
  sig_data <<- apply(sig_data,2,function(p){p.adjust(p,method="fdr")})
  # Create the matrix
  corplot2(cor_data, method = "color", col = custom_col,
           addCoef.col = "black", number.font = 1,
           tl.col = "black", tl.cex = 1, tl.srt = 45,
           p.mat = sig_data, sig.level = c(0.0001, 0.001, 0.01), cl.cex = 1,
           insig = "label_sig", pch.cex = 1, pch.col = "black")
  # Label
  mtext(title_text, side = 3, line = 2, cex = 1.25)
}

# Function to create the correlation matrix with specific neurologic functions (NeurEx)
createCorMatrixSpecific <- function(data, title_text) {
  # Get the r values
  cor_data <<- cor(data[, c(9, 10, 11, 13, 16)], data[, c(25:43)], method = "spearman",
                   use = "complete.obs")
  rownames(cor_data) <- c("Path Length", "Time in Center", "Average Distance\nfrom
Center",
```

```
                        "Average Speed in\nCenter", "Direction Changes")
  # Get the p values
  sig_data <- rcorr(as.matrix(cbind(data[, c(9, 10, 11, 13, 16)], data[, c(25:43)])),
                    type = "spearman")$P
  sig_data <- sig_data[1:5, 6:24]
  sig_data <<- apply(sig_data,2,function(p){p.adjust(p,method="fdr")})
  # Create the matrix
  corplot2(cor_data, method = "color", col = custom_col,
           addCoef.col = "black", number.font = 1,
           tl.col = "black", tl.cex = 1, tl.srt = 45,
           p.mat = sig_data, sig.level = c(0.0001, 0.001, 0.01), cl.cex = 1,
           insig = "label_sig", pch.cex = 1, pch.col = "black")
  # Label
  mtext(title_text, side = 3, line = 2, cex = 1.25)
}

# Create the correlation matrices
png("/Users/oliviafan/Documents/Research/NIH 2018/Level Test/Data/Correlations/Clinical
Scales.png",
    units="in", width=14, height=5, res=600)
par(mfrow = c(1, 2))
createCorMatrixScales(dom_xsec, "Dominant")
createCorMatrixScales(ndom_xsec, "Non-dominant")
mtext("*** p < 0.0001, ** p < 0.001, * p < 0.01", outer = T, side = 1, line = -3)
mtext("         B", outer = T, side = 3, adj = 0, line = -2.1, font = 2, cex = 1.5625)
mtext("Non-dominant Cross-sectional", side = 2, outer = T, line = -2, cex = 1.25)
dev.off()

png("/Users/oliviafan/Documents/Research/NIH 2018/Level
Test/Data/Correlations/Neurological Functions.png",
    units="in", width=14, height=7, res=600)
```

```r
par(mfrow = c(1, 2))
createCorMatrixSpecific(dom_xsec, "Dominant")
createCorMatrixSpecific(ndom_xsec, "Non-dominant")
mtext("*** p < 0.0001, ** p < 0.001, * p < 0.01", outer = T, side = 1, line = -3)
mtext("        B", outer = T, side = 3, adj = 0, line = -2.1, font = 2, cex = 1.5625)
dev.off()


# --------------------------------------------------------------------------
# Export modified datasheets
# --------------------------------------------------------------------------

# Rescale path length to be by pixels instead of millimeters
dom_xsec$Path_Length_.mm. <- dom_xsec$Path_Length_.mm.*21.176457
ndom_xsec$Path_Length_.mm. <- ndom_xsec$Path_Length_.mm.*21.176457

# Rename
names(dom_xsec)[9] <- "Path Length (px)"
names(ndom_xsec)[9] <- "Path Length (px)"

write.table(dom_xsec,
        file="/Users/oliviafan/Documents/Research/NIH 2018/Level Test/Data/Edited
Spreadsheets/Dom_Xsec_Neuro.csv",
        sep=",", row.names=F)
write.table(ndom_xsec,
        file="/Users/oliviafan/Documents/Research/NIH 2018/Level Test/Data/Edited
Spreadsheets/Nondom_Xsec_Neuro.csv",
        sep=",", row.names=F)
```

```
# ---------------------------------------------------------------------------
# Olivia Fan
# Created 05/20/2018
# Edited 07/17/2018
# Boxplots
# This script creates figure 4 and creates boxplots.
# ---------------------------------------------------------------------------

# ---------------------------------------------------------------------------
# Read data
# ---------------------------------------------------------------------------

# Read the data
dom_xsec <- read.csv("/Users/oliviafan/Documents/Research/NIH 2018/Level
Test/Data/Edited Spreadsheets/09_Dom_Xsec.csv", header=T)
dom_long <- read.csv("/Users/oliviafan/Documents/Research/NIH 2018/Level
Test/Data/Edited Spreadsheets/09_Dom_Long.csv", header=T)
ndom_xsec <- read.csv("/Users/oliviafan/Documents/Research/NIH 2018/Level
Test/Data/Edited Spreadsheets/09_Nondom_Xsec.csv", header=T)
ndom_long <- read.csv("/Users/oliviafan/Documents/Research/NIH 2018/Level
Test/Data/Edited Spreadsheets/09_Nondom_Long.csv", header=T)

# Rescale path length to be by pixels instead of millimeters
dom_xsec$Path_Length_.mm. <- dom_xsec$Path_Length_.mm.*21.176457
ndom_xsec$Path_Length_.mm. <- ndom_xsec$Path_Length_.mm.*21.176457
dom_long$Path_Length_.mm. <- dom_long$Path_Length_.mm.*21.176457
ndom_long$Path_Length_.mm. <- ndom_long$Path_Length_.mm.*21.176457

# Change group numbers to "MS" and "HV"
dom_xsec$Group[dom_xsec$Group == 1] <- "MS"
dom_xsec$Group[dom_xsec$Group == 2] <- "HV"
```

```
ndom_xsec$Group[ndom_xsec$Group == 1] <- "MS"
ndom_xsec$Group[ndom_xsec$Group == 2] <- "HV"
dom_long$Group[dom_long$Group == 1] <- "MS"
dom_long$Group[dom_long$Group == 2] <- "HV"
ndom_long$Group[ndom_long$Group == 1] <- "MS"
ndom_long$Group[ndom_long$Group == 2] <- "HV"

# Convert time in center to seconds instead of milliseconds
dom_xsec$Time_Center_.ms. <- dom_xsec$Time_Center_.ms./1000
ndom_xsec$Time_Center_.ms. <- ndom_xsec$Time_Center_.ms./1000
dom_long$Time_Center_.ms. <- dom_long$Time_Center_.ms./1000
ndom_long$Time_Center_.ms. <- ndom_long$Time_Center_.ms./1000

# Change longitudinal to include xsec and long
dom_long <- rbind(dom_long, dom_xsec)
ndom_long <- rbind(ndom_long, ndom_xsec)
dom_long <- dom_long[!duplicated(dom_long),]
ndom_long <- ndom_long[!duplicated(ndom_long),]

# ---------------------------------------------------------------------------
# Boxplots
# ---------------------------------------------------------------------------

# Colors
blue <- c(0, 102, 255)
orange <- c(255, 153, 0)

# Function to create boxplots for cross-sectional data using a Wilcoxon
# rank-sum test to determine the W and p-value
createBoxplotXsec <- function(data, measure_col, ylabel, xlabel, add_y, rgb1, rgb2) {
  # Split the data by group
```

```
  data1 <- data[data$Group == "MS", measure_col]
  data2 <- data[data$Group == "HV", measure_col]
  # Extract the rgb values
  r1 <- rgb1[1]
  g1 <- rgb1[2]
  b1 <- rgb1[3]
  r2 <- rgb2[1]
  g2 <- rgb2[2]
  b2 <- rgb2[3]
  # Apply a Wilcoxon rank-sum test
  wtest <- wilcox.test(data1, data2)
  W.val <- wtest$statistic
  p.val <-signif(wtest$p.value, 4)
  # Determine the y limits
  ymin = 0
  ymax = max(data[, measure_col], na.rm = T)
  # Make the color palettes
  col_pal = c(rgb(red = r1, green = g1, blue = b1, max = 255), rgb(red = r2, green = g2, blue =
b2, max = 255))
  col_pal_light = c(rgb(red = r1, green = g1, blue = b1, alpha = 128, max = 255),
                    rgb(red = r2, green = g2, blue = b2, alpha = 128, max = 255))
  # Create the boxplot
  # If the measure is velocity in center, use a log scale
  if (measure_col == 13) {
    ymin = min(data[, measure_col])
    boxplot(data[, measure_col]~data$Group, log = "y", ylim = c(ymin,
(ymax/ymin)^1.28*ymin),
          pch = 20, outcol = col_pal_light, col = col_pal_light,
          border = col_pal, whisklty = 1, medlwd = 1, frame.plot = F,
          yaxt = add_y, xlab = xlabel, cex.lab = 1.5)
  }
```

```
  # Else, don't use a log scale
  else {
    boxplot(data[, measure_col]~data$Group, ylim = c(ymin, 1.28*ymax),
            pch = 20, outcol = col_pal_light, col = col_pal_light,
            border = col_pal, whisklty = 1, medlwd = 1, frame.plot = F,
            yaxt = add_y, xlab = xlabel, cex.lab = 1.5)
  }
  means <- c(mean(data[data$Group == "HV",][, measure_col], na.rm = T),
mean(data[data$Group == "MS",][, measure_col], na.rm = T))
  points(means, col = "black", pch = 17)
  # Add y axis label
  mtext(ylabel, side = 2, line = 3, cex = 1)
  # Add p-value with *s: * means p < 0.05, ** means p < 0.01, *** means p < 0.001
  if (p.val < 0.01 & p.val >= 0.001) {
    stars = "*"
  }
  else if (p.val < 0.001 & p.val >= 0.0001) {
    stars = "**"
  }
  else if (p.val < 0.0001) {
    stars = "***"
  }
  else {
    stars = ""
  }
  if (measure_col == 13) {
    text(y = (ymax/ymin)^1.25*ymin, x = c(1:2),
         labels = paste("n =", c(length(data2), length(data1))))
    text(y = (ymax/ymin)^1.15*ymin, x = 1.5,
         labels = stars, cex = 1.5)
  }
```

```
  else {
    text(y = 1.25 * ymax, x = c(1:2),
         labels = paste("n =", c(length(data2), length(data1))))
    text(y = 1.15 * ymax, x = 1.5,
         labels = stars, cex = 1.5)
  }
}

createBoxplotXsecAll <- function() {
  par(mfrow = c(1, 10))
  # Path Length
  par(mar = c(8, 5, 5, 0))
  createBoxplotXsec(dom_xsec, 9, "Path Length (px)", "Dominant", "s",
                    blue, orange)
  par(mar = c(8, 1, 5, 4))
  createBoxplotXsec(ndom_xsec, 9, "", "Non-dominant", "n",
                    blue, orange)
  # Time in Center
  par(mar = c(8, 5, 5, 0))
  createBoxplotXsec(dom_xsec, 10, "Time in Center (s)", "Dominant", "s",
                    blue, orange)
  par(mar = c(8, 1, 5, 4))
  createBoxplotXsec(ndom_xsec, 10, "", "Non-dominant", "n",
                    blue, orange)
  # Average Distance from Center
  par(mar = c(8, 5, 5, 0))
  createBoxplotXsec(dom_xsec, 11, "Average Distance from Center (px)", "Dominant", "s",
                    blue, orange)
  par(mar = c(8, 1, 5, 4))
  createBoxplotXsec(ndom_xsec, 11, "", "Non-dominant", "n",
                    blue, orange)
```

```
  # Average Speed in Center
  par(mar = c(8, 5, 5, 0))
  createBoxplotXsec(dom_xsec, 13, "Average Speed in Center (px/s)", "Dominant", "s",
                    blue, orange)
  par(mar = c(8, 1, 5, 4))
  createBoxplotXsec(ndom_xsec, 13, "", "Non-dominant", "n",
                    blue, orange)
  # Directional Changes
  par(mar = c(8, 5, 5, 0))
  createBoxplotXsec(dom_xsec, 16, "Direction Changes", "Dominant", "s",
                    blue, orange)
  par(mar = c(8, 1, 5, 4))
  createBoxplotXsec(ndom_xsec, 16, "", "Non-dominant", "n",
                    blue, orange)
  mtext("Cross-sectional Cohort", outer = T, line = -2, cex = 1.25)
  mtext("                              Path Length                                                    Time in Center
Average Distance from Center                                    Average Speed in Center
Direction Changes",
        outer = T, line = -5, cex = 1, adj = 0)
  mtext("*** p < 0.0001, ** p < 0.001, * p < 0.01", outer = T, side = 1, line = -2, cex = 1)
  mtext("  A", line = -2, outer = T, adj = 0, cex = 1.5625, font = 2)
}

# Run this line to get all boxplots at once
png("/Users/oliviafan/Documents/Research/NIH 2018/Level Test/Data/Boxplots/Cross-
sectional.png",
    units="in", width=17, height=4.5, res=600)
createBoxplotXsecAll()
dev.off()

library(plyr)
```

```r
# Function to average the trials
averageTrials <- function(data) {
  # Split the data by patient
  data$PID <- as.factor(as.character(data$PID))
  data <- split(data, data$PID)
  # Split the data by date
  data <- lapply(1:length(data), function(x) {
    data_patient <- data[[x]]
    data_patient1 <- data_patient[1, 1:7]
    data_patient2 <- t(colMeans(data_patient[, 8:16], na.rm = T))
    data_patient <- cbind(data_patient1, data_patient2)
    return(data_patient)
  })
  data <- ldply(data, data.frame)
  return(data)
}

# Function to create boxplots for longitudinal data using mixed-ANOVA
createBoxplotLong <- function(data, measure_col, ylabel, xlabel, add_y, rgb1, rgb2) {
  # Take the mean for each patient so that each patient only has one point
  data_mean <- averageTrials(data)
  # Split the data by group
  data1 <- data[data$Group == "MS", measure_col]
  data2 <- data[data$Group == "HV", measure_col]
  # Extract the rgb values
  r1 <- rgb1[1]
  g1 <- rgb1[2]
  b1 <- rgb1[3]
  r2 <- rgb2[1]
  g2 <- rgb2[2]
```

```
  b2 <- rgb2[3]
  # Packages used in mixed-ANOVA
  require(car)
  require(lme4)
  # Check for significance of interaction term first (keep as type 3)
  # Since not significant (p=0.485), drop interaction and switch to type 2
  aov_measure <- Anova(lmer(data[,
measure_col]~Days*Group+(Days|PID),data=data),type=3,test.statistic="F")
  aov_measure <- Anova(lmer(data[,
measure_col]~Days+Group+(Days|PID),data=data),type=2,test.statistic="F")
  # Get the F and p values
  F.val <- signif(aov_measure$F[2], 4)
  p.val <- signif(aov_measure$`Pr(>F)`[2], 4)
  # Determine the y limits
  ymin = 0
  ymax = max(data_mean[, measure_col], na.rm = T)
  # Make the color palettes
  col_pal = c(rgb(red = r1, green = g1, blue = b1, max = 255), rgb(red = r2, green = g2, blue =
b2, max = 255))
  col_pal_light = c(rgb(red = r1, green = g1, blue = b1, alpha = 128, max = 255),
                  rgb(red = r2, green = g2, blue = b2, alpha = 128, max = 255))
  # Create the boxplot
  # If the measure is velocity in center, use a log scale
  if (measure_col == 13) {
    ymin = min(data[, measure_col])
    boxplot(data_mean[, measure_col]~data_mean$Group, log = "y", ylim = c(ymin,
(ymax/ymin)^1.28*ymin),
            pch = 20, outcol = col_pal_light, col = col_pal_light,
            border = col_pal, whisklty = 1, medlwd = 1, frame.plot = F,
            yaxt = add_y, xlab = xlabel, cex.lab = 1.5)
  }
```

```
  # Else, don't use a log scale
  else {
    boxplot(data_mean[, measure_col]~data_mean$Group, ylim = c(ymin, 1.28*ymax),
            pch = 20, outcol = col_pal_light, col = col_pal_light,
            border = col_pal, whisklty = 1, medlwd = 1, frame.plot = F,
            yaxt = add_y, xlab = xlabel, cex.lab = 1.5)
  }
  means <- c(mean(data_mean[data_mean$Group == "HV",][, measure_col], na.rm = T),
mean(data_mean[data_mean$Group == "MS",][, measure_col], na.rm = T))
  points(means, col = "black", pch = 17)
  # Add y axis label
  mtext(ylabel, side = 2, line = 3, cex = 1)
  # Add p-value with *s: * means p < 0.05, ** means p < 0.01, *** means p < 0.001
  if (p.val < 0.01 & p.val >= 0.001) {
    stars = "*"
  }
  else if (p.val < 0.001 & p.val >= 0.0001) {
    stars = "**"
  }
  else if (p.val < 0.0001) {
    stars = "***"
  }
  else {
    stars = ""
  }
  if (measure_col == 13) {
    text(y = (ymax/ymin)^1.25*ymin, x = c(1:2),
         labels = paste("n =", c(length(data2), length(data1))))
    text(y = (ymax/ymin)^1.15*ymin, x = 1.5,
         labels = stars, cex = 1.5)
  }
```

```
  else {
    text(y = 1.25 * ymax, x = c(1:2),
         labels = paste("n =", c(length(data2), length(data1))))
    text(y = 1.15 * ymax, x = 1.5,
         labels = stars, cex = 1.5)
  }
}

createBoxplotLongAll <- function() {
  par(mfrow = c(1, 10))
  # Path Length
  par(mar = c(8, 5, 5, 0))
  createBoxplotLong(dom_long, 9, "Path Length (px)", "Dominant", "s",
                    blue, orange)
  par(mar = c(8, 1, 5, 4))
  createBoxplotLong(ndom_long, 9, "", "Non-dominant", "n",
                    blue, orange)
  # Time in Center
  par(mar = c(8, 5, 5, 0))
  createBoxplotLong(dom_long, 10, "Time in Center (s)", "Dominant", "s",
                    blue, orange)
  par(mar = c(8, 1, 5, 4))
  createBoxplotLong(ndom_long, 10, "", "Non-dominant", "n",
                    blue, orange)
  # Average Distance from Center
  par(mar = c(8, 5, 5, 0))
  createBoxplotLong(dom_long, 11, "Average Distance from Center (px)", "Dominant", "s",
                    blue, orange)
  par(mar = c(8, 1, 5, 4))
  createBoxplotLong(ndom_long, 11, "", "Non-dominant", "n",
                    blue, orange)
```

```
  # Average speed in Center
  par(mar = c(8, 5, 5, 0))
  createBoxplotLong(dom_long, 13, "Average Speed in Center (px/s)", "Dominant", "s",
                    blue, orange)
  par(mar = c(8, 1, 5, 4))
  createBoxplotLong(ndom_long, 13, "", "Non-dominant", "n",
                    blue, orange)
  # Directional Changes
  par(mar = c(8, 5, 5, 0))
  createBoxplotLong(dom_long, 16, "Direction Changes", "Dominant", "s",
                    blue, orange)
  par(mar = c(8, 1, 5, 4))
  createBoxplotLong(ndom_long, 16, "", "Non-dominant", "n",
                    blue, orange)
  mtext("Full Cohort", outer = T, line = -2, cex = 1.25)
  mtext("                    Path Length                              Time in Center
Average Distance from Center                    Average Speed in Center
Direction Changes",
        outer = T, line = -5, cex = 1, adj = 0)
  mtext("*** p < 0.0001, ** p < 0.001, * p < 0.01", outer = T, side = 1, line = -2, cex = 1)
  mtext("  B", line = -2, outer = T, adj = 0, cex = 1.5625, font = 2)
}

# Run this line to get all boxplots at once
png("/Users/oliviafan/Documents/Research/NIH 2018/Level Test/Data/Boxplots/Full.png",
    units="in", width=17, height=4.5, res=600)
createBoxplotLongAll()
dev.off()

# Blank sheet for the boxplots
png("/Users/oliviafan/Documents/Research/NIH 2018/Level Test/Data/Boxplots/Figure 3
```

```
Boxplots.png",
    units="in", width=17, height=9, res=600)
plot(c())
dev.off()
```

```r
# ---------------------------------------------------------------------------
# Olivia Fan
# Created 03/01/2018
# Edited 06/29/2018
# 06 Intra-individual Stability
# This script creates figure 5 and graphs how the path length versus time in
# center of each individual changes over time (longitudinal data only).
# ---------------------------------------------------------------------------

# ---------------------------------------------------------------------------
# Read data
# ---------------------------------------------------------------------------

readData <- function(filename) {
  data <- read.csv(filename, header = TRUE, na.strings = c("", "NA"))
  # Change patient IDs from factors to strings
  data$PID <- as.character(data$PID)
  return(data)
}

dom_long <- readData("/Users/oliviafan/Documents/Research/NIH 2018/Level
Test/Data/Edited Spreadsheets/09_Dom_Long.csv")
ndom_long <- readData("/Users/oliviafan/Documents/Research/NIH 2018/Level
Test/Data/Edited Spreadsheets/09_Nondom_Long.csv")

# Change group numbers to "MS" and "HV"
dom_long$Group[dom_long$Group == 1] <- "MS"
dom_long$Group[dom_long$Group == 2] <- "HV"
ndom_long$Group[ndom_long$Group == 1] <- "MS"
ndom_long$Group[ndom_long$Group == 2] <- "HV"
```

```r
# Convert time in center to seconds instead of milliseconds
dom_long$Time_Center_.ms. <- dom_long$Time_Center_.ms./1000
ndom_long$Time_Center_.ms. <- ndom_long$Time_Center_.ms./1000

# Rescale path length to be by pixels instead of millimeters
dom_long$Path_Length_.mm. <- dom_long$Path_Length_.mm.*21.176457
ndom_long$Path_Length_.mm. <- ndom_long$Path_Length_.mm.*21.176457

# ---------------------------------------------------------------------------
# Graph path length versus time in center of every individual
# ---------------------------------------------------------------------------

# Get every person, assign them a color (distinct among HV and MS but not between)
HV_colors <- data.frame(unique(c(dom_long[dom_long$Group == "HV", ]$PID,
ndom_long[ndom_long$Group == "HV", ]$PID)))
MS_colors <- data.frame(unique(c(dom_long[dom_long$Group == "MS", ]$PID,
ndom_long[ndom_long$Group == "MS", ]$PID)))
HV_colors <- cbind(HV_colors, 1:nrow(HV_colors))
MS_colors <- cbind(MS_colors, 1:nrow(MS_colors))
names(HV_colors) <- c("PID", "Color")
names(MS_colors) <- c("PID", "Color")

# Function to graph the path length vs. time in center for dominant/non-dominant hands
graphOverall <- function(data, title) {

  # Custom color palette
  library(RColorBrewer)
  palette(colors()[c(121, 420, 640, 499, 256, 635,
                     53, 142, 8, 30, 642, 96, 450,
                     130, 596, 258, 134, 525, 50,
                     456, 27, 636)])
```

```r
# Split into HV and MS
data_HV <- data[data$Group == "HV",]
data_MS <- data[data$Group == "MS",]

# Determine the HV medians for path length and time in center
pl_med <- median(data_HV$Path_Length)
tc_med <- median(data_HV$Time_Center)

par(mfrow = c(1, 2))

# Healthy volunteers
par(mar = c(5, 6, 4, 0))
plot(c(), c(), xlab = "", ylab = "", log = "y",
     xlim = c(0, 10), ylim = c(1050, 21200))
data_HV <- split(data_HV, data_HV$PID)
lapply (1:length(data_HV), function(x) {
  patient <- data_HV[[x]]
  # Select the color
  y <- match(patient$PID, HV_colors$PID)
  # Sort the data by date
  patient <- patient[order(patient$Days),]
  # Plot the trials of the patient
  points(patient$Time_Center, patient$Path_Length,
         type = "o", col = HV_colors[y, 2], pch = 20)
  # Graph the lines of medians for HV
  abline(h = pl_med, col = "gray")
  abline(v = tc_med, col = "gray")
  # Plot the first trials as a gray circle
  points(patient$Time_Center[1], patient$Path_Length[1], col = "gray", cex = 1.5)
})
```

```
  mtext("HV", side = 3)

  # MS patients
  par(mar = c(5, 3, 4, 3))
  plot(c(), c(), xlab = "", ylab = "", log = "y",
       xlim = c(0, 10), ylim = c(1050, 21200))
  data_MS <- split(data_MS, data_MS$PID)
  lapply (1:length(data_MS), function(x) {
    patient <- data_MS[[x]]
    # Select the color
    y <- match(patient$PID, MS_colors$PID)
    # Sort the data by date
    patient <- patient[order(patient$Days),]
    # Plot the trials of the patient
    points(patient$Time_Center, patient$Path_Length,
           type = "o", col = MS_colors[y, 2], pch = 4)
    # Graph the lines of medians for HV
    abline(h = pl_med, col = "gray")
    abline(v = tc_med, col = "gray")
    # Plot the first trials as a gray circle
    points(patient$Time_Center[1], patient$Path_Length[1], col = "gray", cex = 1.5)
  })
  mtext("MS", side = 3)

  mtext(title, outer = T, line = -2.5)
  mtext("Time in Center (s)", outer = T, side = 1, line = -2)
  mtext("Path Length (px)", outer = T, side = 2, line = -2.5)
}

# Save the dominant hand graph
png("/Users/oliviafan/Documents/Research/NIH 2018/Level Test/Data/Intra-individual
```

```
Stability/Dominant.png",
    units="in", width=7, height=4.35, res=300)
graphOverall(dom_long, "Dominant")
mtext("     A", line = -2.5, outer = T, adj = 0, cex = 1.25, font = 2)
dev.off()

# Save the non-dominant hand graph
png("/Users/oliviafan/Documents/Research/NIH 2018/Level Test/Data/Intra-individual
Stability/Non-dominant.png",
    units="in", width=7, height=4.35, res=300)
graphOverall(ndom_long, "Non-dominant")
mtext("     B", line = -2.5, outer = T, adj = 0, cex = 1.25, font = 2)
dev.off()
```

```
# ---------------------------------------------------------------------------
# Olivia Fan
# Created 08/04/2018
# Edited 08/04/2018
# GA Clean
# This script cleans the data prior to running the genetic algorithm.
# ---------------------------------------------------------------------------

# ---------------------------------------------------------------------------
# Read and clean the data
# ---------------------------------------------------------------------------

# Read the data
dom_xsec <- read.csv("/Users/oliviafan/Documents/Research/NIH 2018/Level
Test/Data/Edited Spreadsheets/Dom_Xsec_Neuro.csv", header=T)
ndom_xsec <- read.csv("/Users/oliviafan/Documents/Research/NIH 2018/Level
Test/Data/Edited Spreadsheets/Nondom_Xsec_Neuro.csv",header=T)
dom_long <- read.csv("/Users/oliviafan/Documents/Research/NIH 2018/Level
Test/Data/Edited Spreadsheets/Dom_Long_Neuro.csv", header=T)
ndom_long <- read.csv("/Users/oliviafan/Documents/Research/NIH 2018/Level
Test/Data/Edited Spreadsheets/Nondom_Long_Neuro.csv",header=T)

# Keep the PID, group, days, and Level test scores
dom_xsec <- dom_xsec[, c(1, 2, 5, 9, 10, 11, 13, 16)]
ndom_xsec <- ndom_xsec[, c(1, 2, 5, 9, 10, 11, 13, 16)]
dom_long <- dom_long[, c(1, 2, 5, 9, 10, 11, 13, 16)]
ndom_long <- ndom_long[, c(1, 2, 5, 9, 10, 11, 13, 16)]

# Append the dominant and non-dominant scores to create one dataset
long <- sapply(1:nrow(ndom_long), function(x) {
  curr_PID <- as.character(ndom_long[x, 2])
```

```
  curr_days <- as.character(ndom_long[x, 3])
  for (y in 1:nrow(dom_long)) {
    if (curr_PID == dom_long[y, 2] & curr_days == dom_long[y, 3]) {
      dom_long_data <- dom_long[y, c(2, 1, 3, 4, 5, 6, 7, 8)]
      ndom_long_data <- ndom_long[x, c(4, 5, 6, 7, 8)]
      return(cbind(dom_long_data, ndom_long_data))
    }
  }
})
long <- do.call(rbind.data.frame, long)
xsec <- sapply(1:nrow(ndom_xsec), function(x) {
  curr_PID <- as.character(ndom_xsec[x, 2])
  curr_days <- as.character(ndom_xsec[x, 3])
  for (y in 1:nrow(dom_xsec)) {
    if (curr_PID == dom_xsec[y, 2] & curr_days == dom_xsec[y, 3]) {
      dom_xsec_data <- dom_xsec[y, c(2, 1, 3, 4, 5, 6, 7, 8)]
      ndom_xsec_data <- ndom_xsec[x, c(4, 5, 6, 7, 8)]
      return(cbind(dom_xsec_data, ndom_xsec_data))
    }
  }
})
xsec <- do.call(rbind.data.frame, xsec)

# Merge longitudinal and cross-sectional data
full <- rbind(xsec, long)
full <- full[!duplicated(full),]

# ---------------------------------------------------------------------------
# Standardize the Level test measures
# ---------------------------------------------------------------------------
```

```r
# Make all the columns numeric
full <- cbind(full[, 1:3], as.data.frame(sapply(full[, 4:13], function(x) as.numeric(x))))
# Subtract the sample mean and divide by the sample standard deviation
full <- cbind(full[, 1:3], as.data.frame(sapply(full[, 4:13], function(x) scale(x))))
names(full) <- c("PID", "Group", "Days", "D_PathLength", "D_TimeCenter",
              "D_AvgDistanceCenter", "D_SpeedCenter", "D_DirectionChanges",
              "ND_PathLength", "ND_TimeCenter", "ND_AvgDistanceCenter",
              "ND_SpeedCenter", "ND_DirectionChanges")

# ---------------------------------------------------------------------------
# Split the data into 2/3 training and 1/3 validation
# ---------------------------------------------------------------------------

require(caTools)
set.seed(6)
require(dplyr)

# MS patients
MS <- full %>% filter(Group == 1)
unique_MS <- unique(MS$PID)
train_MS <- sample(unique_MS, ceiling(2/3*length(unique_MS)), replace = F)
test_MS <- setdiff(unique_MS, train_MS)

# HV patients
HV <- full %>% filter(Group == 2)
unique_HV <- unique(HV$PID)
train_HV <- sample(unique_HV, ceiling(2/3*length(unique_HV)), replace = F)
test_HV <- setdiff(unique_HV, train_HV)

# Merge training and testing IDs
train_PID <- c(as.character(train_HV), as.character(train_MS))
```

```
test_PID <- c(as.character(test_HV), as.character(test_MS))

# Filter patients into training and testing cohorts
train<-full %>% filter(PID %in% train_PID)
test<-full %>% filter(PID %in% test_PID)

# Save the cleaned data sets
save.image(file = "/Users/oliviafan/Documents/Research/NIH 2018/Level
Test/Data/GA/Dif_Clean_Env.RData")
```

```
# ---------------------------------------------------------------------------
# Olivia Fan
# Created 08/04/2018
# Edited 08/04/2018
# GA Coef, adapted from scripts written by Ann Marie
# Weideman
# This script gets the coefficients of the full and reduced models.
# ---------------------------------------------------------------------------

# ---------------------------------------------------------------------------
# Load the cleaned data
# ---------------------------------------------------------------------------

load(file = "/Users/oliviafan/Documents/Research/NIH 2018/Level
Test/Data/GA/Dif_Clean_Env.RData")

# ---------------------------------------------------------------------------
# Define the fitness models for the full model and the reduced model
# ---------------------------------------------------------------------------

# Packages used in mixed-ANOVA
require(car)
require(lme4)

model_lm <- function(input, sigma, PID, group, days) {
  # Randomly vary coefficients on values within search space
  y <- input %*% sigma
  # Linear model
  # Fit the lmer
  fit <<- Anova(lmer(y~group+days+(days|PID)), type = 2, test.statistic = "F")
  return(fit$F[1])
```

```
}

level_lm <- function(PID, group, days, measure) {
  # Fit the lmer
  fit <- Anova(lmer(measure~group+days+(days|PID)), type = 2, test.statistic = "F")
  return(fit$F[1])
}

# ---------------------------------------------------------------------------
# Command line arguments
# ---------------------------------------------------------------------------

require(batch)

# Track the simulation number
sim = NULL

parseCommandArgs()

# ---------------------------------------------------------------------------
# Develop the model based on difference between the two groups.
# ---------------------------------------------------------------------------

# Range of weights
min_full <- c(0, -2, 0, 0, 0, 0, -2, 0, 0, 0)
max_full <- min_full + 2

# Function to get the coefficients
getCoef <- function(train_sub, min_weights, max_weights, sim) {

  # Extract the standardized Level measures
```

```r
  train_numeric <- apply(train_sub[, 4:ncol(train_sub)], 2, as.numeric)

  require(GA)
  require(parallel)
  require(doParallel)
  require(future)
  require(doRNG)

  # Run the genetic algorithm
  lm_GA <- ga(type = "real-valued", fitness = model_lm, input = train_numeric,
              PID = train_sub[, 1], group = train_sub[, 2], days = train_sub[, 3],
              lower = min_weights, upper = max_weights,
              parallel = availableCores(), names = colnames(train_numeric), seed = sim,
              maxiter = 20, run = 400, crossover = gareal_blxCrossover)

  # Return the eventual best fitness value (t-score)
  return(lm_GA@solution[1,])

}

# ---------------------------------------------------------------------------
# Get the coefficients of all models
# ---------------------------------------------------------------------------

full_coef <- getCoef(train, min_full, max_full, sim)

write.csv(full_coef,
          file = paste0("/data/weidemanak/MRI/Olivia/Output/Full_Coef_", sim, ".csv"))
```

```
# ---------------------------------------------------------------------------
# Olivia Fan
# Created 08/04/2018
# Edited 08/04/2018
# GA Concat 1, adapted from scripts written by Ann Marie Weideman
# This script concatenates the results of the Full_Coef files and creates the
# plot with the weights.
# ---------------------------------------------------------------------------

# ---------------------------------------------------------------------------
# Read and concatenate the data
# ---------------------------------------------------------------------------

# Load in the environment with the cleaned data
load(file = "/Users/oliviafan/Documents/Research/NIH 2018/Level
Test/Data/GA/Dif_Clean_Env.RData")

# Get the coefficients of the full model
coef <- matrix(ncol = 10, nrow = 210)
for (x in 1:210) {
  if (file.exists(paste0("/Users/oliviafan/Documents/Research/NIH 2018/Level
Test/Data/GA/Output_Dif_Clean/Full_Coef_", x, ".csv"))) {
    curr_coef <- read.csv(paste0("/Users/oliviafan/Documents/Research/NIH 2018/Level
Test/Data/GA/Output_Dif_Clean/Full_Coef_", x, ".csv"))
    curr_coef <- as.numeric(unlist(curr_coef[, 2]))
    coef[x,] <- curr_coef
  }
}
coef <- coef[rowSums(is.na(coef)) < ncol(coef),] # Remove empty rows
coef <- coef[1:200,] # Take the first 200 rows
coef_mean <- apply(coef, 2, mean)
```

```
coef_mean

# ---------------------------------------------------------------------------
# Create the weights plot
# ---------------------------------------------------------------------------

require(plotrix)

# Function to get the 95% confidence interval
getCI <- function(data) {
  n <- length(data) # Sample size
  se <- sd(data)/sqrt(n) # Standard error
  m <- mean(data) # Mean
  cv <- qt(0.975, df = n-1) # Critical value for the t distribution
  c(m - cv * se, m + cv * se) # Return the 95% confidence interval
}

# Full weights
# Get the confidence intervals
coef_CI <- apply(coef, 2, getCI)
coef_names <- c("Dominant Path    \nLength", "Dominant Time in    \nCenter",
                "Dominant Average    \nDistance from Center", "Dominant Average    \nSpeed in
Center",
                "Dominant Direction    \nChanges", "Non-dominant Path    \nLength",
                "Non-dominant Time in    \nCenter", "Non-dominant Average    \nDistance from
Center",
                "Non-dominant Average    \nSpeed in Center", "Non-dominant Direction
\nChanges")

# Get the order to plot in
coef_order <- c(1, 6, 2, 7, 3, 8, 4, 9, 5, 10)
```

```
coef_CI <- coef_CI[1:2, coef_order]
coef_mean <- coef_mean[coef_order]
coef_names <- coef_names[coef_order]
coef <- coef[, coef_order]

# Create the picture
png("/Users/oliviafan/Documents/Research/NIH 2018/Level
Test/Data/GA/Full_Weights_CI.png", units = "in", width = 15, height = 5, res = 600)
par(mar = c(8, 4, 1, 1))
#plotCI(x = 1:length(coef_mean), y = coef_mean, ylim = c(-2, 1), xaxt = "n", yaxt = "n",
#       xlab = "", ylab = "", pch = 20, li = coef_mean, ui = coef_mean, err = "y")
# Plot the outline
plot(c(), ylim = c(-2, 1), xlim = c(1, 10), xaxt = "n", yaxt = "n", xlab = "", ylab = "")
# Plot the legend
text(y = c(-2, -1.5, -1, -0.5, 0, 0.5, 1), x = 0.45, xpd = T,
     labels = c("-2", "-1.5", "-1.0", "-0.5", "0.0", "0.5", "1.0"))
text(x = 1:10, y = c(-2.2, -2.2, -2.2, -2.2, -2.2, -2.2, -2.2, -2.2, -2.2, -2.2),
     labels = coef_names, xpd = T, srt = 45, adj = 1)
mtext("Relative Weights", side = 2, line = 2.5)
abline(h = 0, col = "lightgray")
abline(h = -2, col = "lightgray")
abline(h = -1.5, col = "lightgray")
abline(h = -1, col = "lightgray")
abline(h = -0.5, col = "lightgray")
abline(h = 0.5, col = "lightgray")
abline(h = 1, col = "lightgray")
# Plot the points
for (i in 1:10) {
  points(x = 1:10, y = coef_mean, pch = 20)
}
text(x = 1:10, y = coef_CI[2,] + 0.2, labels = round(coef_mean, 3))
```

```
dev.off()


png("/Users/oliviafan/Documents/Research/NIH 2018/Level Test/Data/GA/Temp.png", units =
"in", width = 15, height = 16, res = 600)
plot(c())
dev.off()
```
